# Combining extracellular matrix proteome and phosphoproteome of chickpea and meta‐analysis reveal novel proteoforms and evolutionary significance of clade‐specific wall‐associated events in plant

**DOI:** 10.1002/pld3.572

**Published:** 2024-03-18

**Authors:** Kanika Narula, Arunima Sinha, Pooja Choudhary, Sudip Ghosh, Eman Elagamey, Archana Sharma, Atreyee Sengupta, Niranjan Chakraborty, Subhra Chakraborty

**Affiliations:** ^1^ National Institute of Plant Genome Research New Delhi India; ^2^ Plant Pathology Research Institute Agricultural Research Center (ARC) Giza Egypt

**Keywords:** cell wall maintenance system, *Cicer arietinum*, extracellular matrix and plant matrisome, mass spectrometry, organellar proteome and phosphoproteome network

## Abstract

Extracellular matrix (ECM) plays central roles in cell architecture, innate defense and cell wall integrity (CWI) signaling. During transition to multicellularity, modular domain structures of ECM proteins and proteoforms have evolved due to continuous adaptation across taxonomic clades under different ecological niche. Although this incredible diversity has to some extent been investigated at protein level, extracellular phosphorylation events and molecular evolution of ECM proteoform families remains unexplored. We developed matrisome proteoform atlas in a grain legume, chickpea and performed meta‐analyses of 74 plant matrisomes. MS/MS analysis identified 1,424 proteins and 315 phosphoproteins involved in diverse functions. Cross‐species ECM protein network identified proteoforms associated with CWI maintenance system. Phylogenetic characterization of eighteen matrix protein families highlighted the role of taxon‐specific paralogs and orthologs. Novel information was acquired on gene expansion and loss, co‐divergence, sub functionalization and neofunctionalization during evolution. Modular networks of matrix protein families and hub proteins showed higher diversity across taxonomic clades than among organs. Furthermore, protein families differ in nonsynonymous to synonymous substitution rates. Our study pointed towards the matrix proteoform functionality, sequence divergence variation, interactions between wall remodelers and molecular evolution using a phylogenetic framework. This is the first report on comprehensive matrisome proteoform network illustrating presence of CWI signaling proteins in land plants.

## 1 INTRODUCTION

Evolutionary shift from unicellular to complex multicellular organization ensued independently in six eukaryotic groups, including plants (Grosberg & Strathmann, 2007). Molecular adaptation and diversification of tracheophytes emerged as the most significant transitions in the plant evolution during mid‐Paleozoic Era between 480 and 360 million years ago on livable Planet (Sørensen et al., 2010). Understanding the selection forces and their effects at the cellular and molecular levels is crucial to study how species have evolved over time. Molecular evolution, which varies between protein families, is controlled by selective constraints on proteins or amino acids (Miyazawa, 2011). Comparative studies at the level of amino acids investigate adaptive evolution, duplication status, gene loss, codivergence, indels, and pairwise sequence comparison by utilizing positive selection, site, and branch models and calculating the ratio of nonsynonymous to synonymous substitution rates (ω) in various lineages among taxa (de la Torre et al., 2017).

Plant multicellularity is governed by cell wall integrity (CWI) maintenance system, which plays a crucial role in the molecular evolution of several plant lineages. Extracellular matrix (ECM) is the key organelle for plant biomass and acts as a carbon reservoir (Cannon et al., 2008; Gilbert, 2010; Hoffmann et al., 2021). Principles of ECM designs are known to have evolved over the last billion years, providing competitive advantage for species survival (Dehors et al., 2019; Sarkar et al., 2009; Vaahtera et al., 2019). A complex network of cross‐linked carbohydrate‐proteins in ECM acts as a bridge between cells and cell types and governs cell morphogenesis, division, and development (Cannon et al., 2008). Based on the evidence of architectural characteristics, composition, and functionality, this network responds to cellular, biophysical, and biochemical stimuli by providing tensile strength, mechanical anisotropy, and load‐bearing compounds (Codjoe et al., 2022). ECM, most likely the largest biological system, houses dynamic and complex scaffolds of proteoforms arising due to alternative splicing or editing of a single gene or post‐translational modifications, which determine its multifunctional nature (Canut et al., 2016; Jamet et al., 2006). Matrix proteins are ubiquitous, rich in one or two aminoacids, and contain repetitive sequence domains and relatively abundant in land plants and green algae (Cassab, 1998). Several evidences suggest that proteins associated with wall biosynthesis and remodeling are differentially regulated in wall biosynthesis mutants (Seifert & Blaukopf, 2010; Verbančič et al., 2018). Also, cellulose synthesis inhibition, interference with arabinogalactan proteins (AGPs), and exposure to pectin fragments are known to affect wall architecture (Brutus et al., 2010; Guan & Nothnagel, 2004; Liu et al., 2015; Manfield et al., 2004; Moscatiello et al., 2006). Thus, polysaccharide network serves as a scaffold for structural proteins, which constitute ~10–15% of plant ECM and are involved in transduction and integration of growth signals for cell–cell communication (Zhang et al., 2021).

Wall surveillance systems maintain wall integrity, perceive signal components, and trigger sensor proteins to transduce signals to the cell interior (Cabrera et al., 2010; Rui & Dinneny, 2020). Phosphorylation, a reversible post‐translational modification, is a key regulator of intra‐cellular biological processes, including protein folding (conformation), localization, complex formation, and degradation (Friso & van Wijk, 2015). In yeast, CWI signaling is governed by phosphorylation and dephosphorylation of proteins during hypo‐osmotic shock, chitin antagonists, oxidative stress, and high and low pH perturbations (Bermejo et al., 2008; Hamann & Denness, 2011; Marín et al., 2009; Martín et al., 2000). Genetic defects affecting polysaccharide biosynthesis are known to control cellulose matrix ratio and CWI signaling in arabidopsis (Coleman et al., 2009; Desprez et al., 2002; Tsang et al., 2011). Furthermore, in arabidopsis and maize, extracellular phosphorylation has been proposed for xyloglucanase, lectin receptor‐like kinase, peroxidases, and chitinase during wall regeneration (Chivasa et al., 2005; Guerra‐Guimarães et al., 2016; Ndimba et al., 2003). Most of our knowledge on wall integrity maintenance is confined to species‐specific ECM proteoforms, but the mechanism is not well defined across different plant lineages. Despite cataloging of matrix proteins in few plant species, the in‐depth ECM proteoform analysis to understand signal and structural attributes of wall organization and its relation to evolutionary changes across plant kingdom has not been a subject of investigation till date. Moreover, characterization of the ECM phosphoproteome and matrix phosphoprotein networks in plants is still in its infancy.

Cross‐species network analysis of proteoforms facilitates our understanding of synergistic and antagonistic regulation and underpins evolutionary significance and species adaptation by linking molecular targets to integrity signals in complex traits (Podder et al., 2021). Interactions among these proteoforms may provide novel insight into conserved and species‐specific translational mechanisms and regulatory circuitries. Further, the analysis plays a decisive role in identifying functional modules, intra/intermodular hubs, determining selection pressure, gene expansion, and candidate biomarker of biological, agronomic, and evolutionary processes. However, interactions involving extracellular proteins are poorly represented in existing databases. In this instance, we developed and comprehensively examined ECM proteoforms atlas of agronomically important crop legume chickpea and identified 1,424 matrix proteins and 315 phosphoproteins to gain comprehensive understanding of CWI maintenance system. Using our approach, we for the first time extensively identified matrix specific S/T/Y phosphorylation events in plants. To explore further, meta‐analysis and network mapping of 74 matrisomes from diverse angiosperm families were performed to identify commonality or divergence of matrix proteins. Data highlight that matrix proteins expand by duplications, followed by sequence divergence, subfunctionalization and neofunctionalization of paralogs and orthologs. An analysis of 16 organ/cell cultures across 35 families provides evidence of partition of protein functions, or acquisition of new functions in different organs. Taxonomic relationships of 18 matrix proteins within the major families of plant kingdom will provide prevailing view of molecular phylogeny. We further aimed to test differences in the rate of molecular evolution among ECM protein families in different lineages and to understand the possible causes driving such variations. This study for the first time generates plant matrisome and builds cross‐species, organ specific and diverse protein families interaction networks to understand ECM dynamics within a phylogenetic framework.

## 2 MATERIALS AND METHODS

### 2.1 Plant material and growth condition

Chickpea (*Cicer arietinum* L. var WR‐315) seeds were surface sterilized using 2% sodium hypochlorite for 2 min, rinsed in sterile water and germinated. Seedlings were grown for 25 days in agro‐peat and vermiculite (2:1, w/w) at 25 ± 2°C, 50% ± 5% relative humidity under 16 h photoperiod (270 μmol m^−2^ s^−1^ light intensity). Seedlings were harvested forenoon with lights remained switched on. The harvested seedlings comprising of shoot and root were stored at −80°C after quick‐freezing in liquid nitrogen.

### 2.2 ECM isolation and ECM protein extraction

ECM fraction was isolated and purified as described by Elagamey et al. (2020). Briefly, 15 g tissue was ground in liquid nitrogen with .3% (w/w) polyvinylpolypyrollidone (PVPP). Powder was homogenized in 5 mM K_3_PO_4_, pH 6.0, 5 mM DTT, 1 mM PMSF for 2 min, and centrifuged at 1,000*g* for 5 min at 4°C. Pellet was washed 10 times with water. ECM fraction was suspended in three volumes (w/v) of extraction buffer (200 mM CaCl_2_, 5 mM DTT, 1 mM PMSF, .3% (w/w) PVPP) on shaker for 2 h, centrifuged (10,000*g*) for 10 min at 4°C, filtered, concentrated, and dialyzed overnight against 1,000 volumes of deionized water.

### 2.3 Scanning electron microscopy

Scanning electron microscopy (SEM) was performed on collar region of chickpea seedlings as described in Manimaran et al. (2015) with few modifications. In brief, samples were fixed in 2.5% gluteraldehyde in .1 M phosphate buffer (pH 7.4) for 24 h at 4°C, dehydrated in series of graded alcohols and dried in vacuum desiccator. Analysis was conducted and images were taken according to the instruction manual of EVO LS 10.

### 2.4 Purity assessment by transmission electron microscopy and organelle specific enzyme assay

Transmission electron microscopy (TEM) was performed as described in Cannon et al., 2008 with few modifications. An aliquot of isolated chickpea ECM fraction was fixed in 2% (v/v) paraformaldehyde/2.5% (v/v) glutaraldehyde for 24 h at 4 °C and washed with .1 M phosphate buffer (pH 7.4). Fixed fraction was treated with 1% osmium tetroxide and dehydrated sequentially in acetone and embedded in epoxy resin. Ultrathin section was stained with uranyl acetate and lead citrate for 15 min and examined by TEM (Morgagni 268D).

Glucose‐6 phoshate dehydrogenase (EC1.1.1.49), catalase, and vanadate‐inhibited H^+^ ATPase activities were determined in triplicates as described in Simcox et al. (1977) and Elagamey et al. (2017). In brief, glucose‐6 phoshate dehydrogenase activity was determined in 10 μg each of ECM protein extract and crude protein extract by spectrophotometer. Assay reaction involved 10 mM MgCl_2_, .1%, triton X‐100, .17 mM NADP^+^, .33 mM glucose 6‐phosphate, 20 mM TES (pH 7.5), and reduction of NADP^+^ was measured at 340 nm. For catalase assay, 10 μg of extracted ECM proteins was incubated in 70 mM potassium phosphate buffer (pH 7.5) with and without 25 mM H_2_O_2_. Decrease in absorbance was determined at 240 nm for 5 min, and change in absorbance was plotted against time.

Vanadate‐sensitive H^+^ ATPase activity was determined as described by Shimazaki and Kondo (1987). In brief, 20 μg ECM protein, plasma membrane, and crude extract was incubated in 120 μl of reaction mixture (50 mM Tris‐MES [pH 6.8], 2 mM Tris‐ATP, 5 mM MgSO_4_, 50 mM KC1, and l mM EDTA) in presence and absence of .1 mM orthovanadate. Before boiling, .05% Triton X‐100 was added. To initiate the reaction, ATP was added at 37°C for 30 min, and release of inorganic phosphate was determined. Enzyme activity was calculated as described in Okumura and Kinoshita (2016).

### 2.5 1‐DE, 2‐DE, image analysis, and in‐gel trypsin digestion

ECM proteins were diluted in 100 mM Tris‐Cl (pH 8.5), 20% (v/v) glycerol, 4% (w/v) SDS, 20 mM DTT, and 1 mM PMSF, boiled for 5 min and centrifuged at 10,000*g* for 10 min at 4°C. Pellets were washed twice with 80% acetone, air‐dried, and resuspended in 8 M urea, 2 M thiourea, 4% (w/v) CHAPS, 20 mM DTT, .5% (v/v) Pharmalyte, and .05% (w/v) bromophenol at 25°C. Protein concentration was determined by Bradford assay. ECM proteins (150 μg) along with low range unstained marker (Bio‐Rad) were separated on a 1‐DE. Stacking gel was prepared in 4% acrylamide/bisacrylamide (40%/2%), and resolving gel of 12.5% acrylamide/bisacrylamide (40%/2%) was subjected to constant voltage of 50 V for 30 min and then 70 V. Proteins were stained with CBB Staining G‐250. Gel images were scanned with Bio‐Rad FluorS system (GE, Healthcare, Japan).

For 2‐DE, ECM protein (1,000 μg) was loaded onto 24 cm IEF strips (pH 4–7) by an in‐gel rehydration. Isoelectric focusing (IEF) was carried out on Ettan IPGphor II (GE Healthcare, Piscataway, N) at 20°C for total accumulated voltage of 80,000 V‐h. Focused strips were reduced with 1% (w/v) DTT in 6 M urea, 50 mM Tris–HCl, pH 8.8, 30% (v/v) glycerol, and 2% (w/v) SDS, followed by alkylation with 2.5% (w/v) iodoacetamide in same buffer for 30 min. Strips were loaded on 12.5% polyacrylamide gels, and SDS‐PAGE was performed at constant current of 60 mA. To detect the ECM phosphoproteins, 2‐D gels were treated with fixation solution (50% methanol and 10% glacial acetic acid) overnight and washed with deionized water five times for 30 min. Gels were stained with Pro‐Q Diamond phosphoprotein gel stain solution for 2 h on shaker protected from light and destained using 20% acetonitrile and sodium acetate (pH 4), three times each for 30 min. Stained gels were scanned using Typhoon scanner 9210 with emission wavelength 580 nm. After acquiring the image, same gels were then stained with silver stain plus kit (Bio‐Rad, United States) to detect ECM proteins (Figure S21a,b).

Silver stained 2‐DE gel were scanned with Bio‐Rad FluorS system, processed and analyzed with PDQuest software (v7.2.0) (Agrawal et al., 2013). Matchset representing “standard image” of three replicates was created, low quality spots (<30 quality score) were removed, and correlation coefficient was maintained to at least .8 between replicates gels. Molecular weight and *p*I were assigned from MrpI parameter in software. In‐gel digestion of protein bands or spots was performed using trypsin (Sigma, United States) according to standard techniques (Elagamey et al., 2017).

### 2.6 Peptide preparation and strong cation‐exchange chromatography fractionation

ECM protein (2 mg) was also subjected to in‐solution trypsin digestion. In brief, ECM proteins were reduced by 5 mM DTT in 50 mM Tris_HCl, pH 8.0, and incubated for 30 min at 37°C. Sample was kept at room temperature prior to the carbamidomethylation by 20 mM iodoacetamide (Sigma‐Aldrich) and then alkylated for 30 min at room temperature in darkness. The reaction was then quenched with DTT for 30 min. To facilitate proteolysis with trypsin, the sample was diluted fourfold with 100 mM NH_4_HCO_3_ to keep the final urea concentration of 2 M. Trypsin was added at an enzyme/protein ratio of 1/50 and proteolysis continued for 16 h incubated at 37°C overnight. To inactivate trypsin, formic acid was added to the sample to adjust pH 3. Sample was speed vac to dryness. Prior to LC‐MS/MS, desalting of the sample was performed with an Top tip POROS R2 (Glygen corp) as per manual instruction. Peptides were eluted with 60% acetonitrile (ACN), .1% trifluoroacetic acid (TFA), and dried in vacuo. Strong cation‐exchange chromatography (SCX) was performed on a 1‐ml ICAT cation exchange column (ABsciex). The precleared peptide mixture was loaded onto a 100 μl injection loop and separated with a linear gradient from buffer A (30% ACN, 500 mM ammonium formate, pH 2.5) to buffer B (30% ACN, 8 mM ammonium formate, pH 3.0) at a flow rate of .5 ml/min. Fractions (fractions 1–6) corresponding to 30, 60, 100, 150, 200, and 350 mM and .5 ml were collected and frozen at −70°C until further analysis.

### 2.7 Metal oxide (TiO_2_) and immobilized metal (Ga^3+^) affinity chromatography‐based phosphopeptide and phosphoprotein enrichment of ECM proteins

Before phosphopeptide enrichment, ECM proteins were either directly subjected to in‐solution digestion or were resolved on 1‐DE as described above and in‐gel digested. Both the approach peptides were dried in vacuo and dissolved in binding buffer (80% ACN .1% TFA). The samples were aspirated in pre‐equilibrated TiO_2_ columns (NuTip, TiO_2_ Glygen Corporation), for 1 h at 4°C, and washed sequentially with binding buffer and washing buffer (60%ACN/.1% TFA). Finally, the phosphopeptides were eluted with 30 μl elution buffer (50 mM ammonium bicarbonate pH 11.5). TiO_2_ fractions were combined and lyophilized to ~50 μl and acidified in .1% TFA for further LC‐MS/MS analysis.

IMAC procedure was performed as described earlier (Tang et al., 2008) with few modifications. Six‐milligram ECM proteins were diluted with wash buffer (8 M urea, 50 mM sodium acetate [pH 4.0], .25% CHAPS) to 1 mg/ml and were dissolved in IMAC buffer (8 M urea, 2 M thiourea, 2% CHAPS). Proteins were then incubated with gallium charged chelating sepharose fast flow beads (GE Healthcare) packed into homemade micro columns for 3 h at 4°C (10 mg protein per milliliter bed volume). Beads were washed with 20 column volumes of wash buffer, and phosphoproteins were eluted using three column volumes of elution buffer (8 M urea, 50 mM Tris acetate [pH 7.4], .1 M EDTA, .1 M EGTA, .25% CHAPS). The eluted proteins were precipitated with five volumes of methanol and resolved into 1‐DE. In‐gel digestion of phosphoprotein bands was performed using trypsin (Sigma, United States) according to standard techniques (Elagamey et al., 2017). Also, IMAC enriched phosphoproteins were desalted and subjected to in‐solution trypsin digestion as described above followed by LC‐MS/MS analysis.

### 2.8 LC‐MS/MS analysis, database search, and motif analysis

The pooled and dried peptides were reconstituted in .1% formic acid to ~.5 μg/μl, loaded on the trap column (3 cm × 200 μm i.d.), desalted, and separated in linear gradient of water/ACN/.1% formic acid (v/v) (A, 95/5/.1%; B, 5/95/.1%) on a ChromXP C18 (3 μm, 100 Å 75 μm ID_15 cm) analytical column at a flow rate of 300 nl/min. Peptides were eluted in gradient of 10–40% ACN (.1% formic acid) over 80 min. Data acquisition was performed with a TripleTOF 5600 System (AB SCIEX) coupled to an Eksigent NanoLC‐Ultra 2D plus system using an ion spray voltage of 2.2 kV, curtain gas of 20 PSI, nebulizer gas of 6 PSI, and an interface heater temperature of 150°C in an information dependent acquisition (IDA) mode with a TOF/MS survey scan (350–1,250 m/z) and accumulation time of 250 ms. A maximum of 10 precursor ions per cycle were selected for fragmentation, and each MS/MS spectrum was accumulated for 100 ms (100–1,500 m/z) with a total cycle time of ~2.3 s. Only the parent ions with a charge state from +2 to +5 were included in the MS/MS fragmentation.

Raw data files (.wiff) were converted into .mgf file format by ProteinPilot™ software (version 4.2 AB SCIEX, United State). Protein identification was performed using the Mascot search engine v.2.3.02 (Matrix Science, United Kingdom) against a Viridiplantae (75,925,788 sequences and 27,045,014,025 residues). BlastP was performed against chickpea 7,563,811 sequences for the identification mapped to organism other than chickpea. The database search parameters were carbamidomethyl (C) as fixed modification, oxidation (M), and phosphorylation (STY) as variable modification. Mixed Glu‐C and trypsin specificity (KR/P, E) was adopted, and up to two missed cleavage sites were allowed. The mass tolerance for the precursor ion was set to 50 ppm. The MS/MS tolerance was .1 Da for TripleTOF 5600. Details of MS/MS analysis have been provided in Tables S1 and S2. Ascore algorithm was used to evaluate the localization probability of phosphorylation sites. We considered only those proteins whose molecular weight search score was above the significant threshold level determined by Mascot *p* < .05 and False Discovery Rate (FDR) < .01 (Table S2). Protein functions were assigned using Pfam or InterPro (Tables S7 and S8). Proteins and phosphoproteins identified with single peptide and respective MS/MS spectra are provided in Tables S9 and S10. Functional annotation of the proteins was conducted using Blast2GO (https://www.blast2go.com/) against the NCBI non‐redundant protein database. The KEGG (http://www.genome.jp/kegg/) and COG databases (http://www.ncbi.nlm.nih.gov/COG/) were used to classify the identified proteins. Sub‐cellular localization, signal peptides, no retention signal, and transmembrane helices prediction were performed using WoLFPSORT (https://wolfpsort.hgc.jp), mSubP (http://bioinfo.usu.edu/Plant-mSubP/), SignalP5.0 (http://www.cbs.dtu.dk/services/SignalP/), TMHMM (http://www.cbs.dtu.dk/services/TMHMM/), and literature search.

Phosphopeptide sequences for phosphorylation sites were pre‐aligned, and their lengths were adjusted to ±6 amino acids from the central position and submitted to the Motif‐X algorithm (http://motif-x.med.harvard.edu) as foreground, and viridiplantae was submitted as background. The occurrence threshold was set at a minimum of four peptides. *p* value of <10^−6^ was considered significant.

### 2.9 Meta‐analysis of data sets, network construction, and in silico analysis

The data analysis was based on 74 available ECM proteomes downloaded from WallProtDB (https://peroxibase.scsv.ups‐tlse.fr/WallProtDB/). Additional data sets were obtained from literature search to improve the coverage (Tables S3 and S4). For meta‐analyses, redundant sequences were eliminated from data sets. The most probable sequence was identified based on existing knowledge, UniProtkb (https://www.uniprot.org), RAP‐DB (rapdb.dna.sffrc.go.jp), and phytozome (http://jgi.doe.gov). Sequences for each data set were then sorted based on the plant ECM protein classification and converted to fasta format as described by Jamet et al. (2006). Plant matrisome atlas adopted and assembled experimentally ECM proteins from different organs and species across kingdom. Further, the complete proteome of 74 organisms was downloaded from UniProt (https://www.uniprot.org/help/proteomes_manual) and clubbed with *C. arietinum* proteome. Combined pooled dataset were subjected to OrthoVenn software for unbiased clustering of proteins. Clusters containing ECM proteins were aligned using MAFFT and then trimmed using Trimal. Phylogenetic tree was generated using maximum likelihood RAXXML. Data were manually curated using Archaeopteryx. Tree was extracted in newick format and visualized in ITOL (https://itol.embl.de/upload.cgi).

Network was built based on the meta‐analysis in association with known protein–protein interaction data from Database of Interacting Proteins (DIP) (https://www.ncbi.nlm.nih.gov/pubmed/11752321). Protein–protein interactions (PPI) were searched against geneMANNIA, BAR, STRING, mentha, Interoporc, IntAct, ‐DIP, APID, MINT, and BIND PPI databases, and the in silico generated IRP network was visualized with Cytoscape version 3.2 (http://cytoscape.org) using Prefuse Force Directed Layout with .01 alpha value cut‐off. All self‐correlations and duplicate protein entries were removed to maintain the data non‐redundancy. We used the following strategy: homology search of ECM protein sequences was performed using BLAST against the SwissProt database. Top SwissProt BLAST hit was assigned to each sequence. The table of SWISS PROT IDs of diverse taxonomic species, organs, and protein families was submitted to Cytoscape. Cytoprophet Maximum Likelihood Approach algorithm was used to generate a probabilistic model of network falling under specific category rejecting others on the basis of lack of interaction data or low probability score (Morcos et al., 2008). Further, we submitted SwissProt IDs to cytoprophet plugin in Cytoscape, which computes the probability of two proteins to potentially interact with each other with DIP as its reference. Further, we have built chickpea proteoform network based on the available data. Modules were computed on the PPI network using the cytoscape MCODE plugin (https://www.ncbi.nlm.nih.gov/pmc/articles/PMC149346/). GO ontology mapping and literature search were performed on datasets. GO enrichment analysis was conducted with the AgriGO Web‐based tool (http://bioinfo.cau.edu.cn/agriGO/analysis.php) using the LegumeIPv2.

### 2.10 Evolutionary analysis of plant kingdom and ECM protein families

NCBI taxonomic ID of plant species, genus, families, clade, or kingdom was retrieved in phyloT (https://phylot.biobyte.de/index.cgi). Phylogenetic tree saved as phylip tree was built from NCBI taxonomic ID in ETE Toolkit Phylogenetic tree (newick) viewer (http://etetoolkit.org/treeview/). Tree was extracted in newick format and visualized in ITOL (https://itol.embl.de/upload.cgi).

Maximum likelihood trees were generated using the amino acid sequences of the protein domains with 1,000 bootstrap replicates in the MEGA software (Tamura et al., 2007). For protein sequences containing multiple domains of the same type, the domains were separated, and all domain sequences were included in the analysis. Multiple sequence alignments corresponding to conserved motifs regions were determined by Clustal X with a gap open and the gap extension penalties of 10 and .1, respectively.

### 2.11 Expansion of ECM protein families, estimation of sequence divergence rate and absolute rates of silent‐site divergence

Available plant ECM proteomes were compared against hidden Markov models (HMMs) of protein families (Wilson et al., 2009). The Pearson's correlation coefficient between samples was calculated and plotted using the “corrplot” package in R for different functional classes of ECM protein family. An All versus All BLASTp search was performed on the combined FASTA file of available ECM proteomes. Heat maps were generated on R with scripts based on the heatmap.2 function as available in the gplots Bioconductor package.

Next, all ECM proteomes including legumes such as *C. arietinum*, *Glycine max*, *Medicago sativa*, *Medicago truncatula*, *Phaseolus lunatus*, *Pisum sativum*, and *Vigna unguiculata* were compared against HMMs of protein families in the SUPERFAMILY database (http://supfam.cs.bris.ac.uk/SUPERFAMILY/howto_use_models.html) using scripts from database and the hmmscan binary from HMMER3 (default e‐value .01) (Eddy, 1995; Wilson et al., 2009). For each SUPERFAMILY present in *C. arietinum*, a Z score was calculated as (number of SUPERFAMILY genes related to ECM in *C. arietinum−*mean number of SUPERFAMILY genes related to ECM in all ECM proteomes)/SD of SUPERFAMILY genes in ECM proteomes. Z‐score values were obtained using the criteria such as *C. arietinum* against all other ECM proteomes and *C. arietinum* against all other proteomes except other Legumes. Z‐score value above 2 with at least three members in *C. arietinum* for a given SUPERFAMILY is considered to be expanded.

CDS sequences of the 6,318 ECM proteins classified into 18 ECM families were obtained and codon alignment was performed using codoncode (https://www.codoncode.com/aligner/sequencealignment.htm#Multiple_sequence_alignment). Alignments were refined using trimAlv1.4.rev15, and members containing more than 30% gaps or ambiguous sites were not kept for further analyses (Capella‐Gutiérrez et al., 2009). The codon alignments were then subjected to Ka/Ks calculation. A number of synonymous substitutions per site (dS), nonsynonymous substitutions per site (dN), and nonsynonymous/synonymous rate ratio (ω or dN/dS) were pairwise estimated for ECM protein family members using the maximum likelihood method. We discarded genes with ω > 10, dS values lower than .01, as these values may result in inaccurate estimates of ω, and also genes with dS or dN > 3, which suggest saturation of substitutions (de la Torre et al., 2017). We also calculated dS, dN, and ω for each terminal branch of the phylogeny using the model of Miyamoto and Fitch in Ka/Ks calculator (Wang et al., 2010).

Absolute rates of silent‐site divergence were estimated for each taxonomic species having 18 ECM protein family separately using the formula μ = dS/2 T, where μ is the synonymous divergence rate per site per year, dS is the mean of synonymous substitutions per site, and T is the time of divergence in years assuming the molecular clock hypothesis (Gaut et al., 2011; Kimura & Ohta, 1971). Synonymous substitutions (dS) for ECM protein family genes were averaged for species in each of the taxonomic families. Divergence times (T) were based on fossil records available in timetree (http://www.timetree.org/). Whenever possible, we selected the median time of divergence between the species in each taxonomic group.

### 2.12 Reconciliation analysis

Evolution of the gene family between the gene trees and rooted species tree was analyzed using reconciliation program, Notung 2.9. The sequences were aligned using MAFFT and trimmed with TrimAL. The phylogenetic tree has been created using PhyML with default parameters. Nodes of the species tree have been collapsed to represent only the following taxonomic ranks: species, genus, family, order, class, phylum, and kingdom. Rearrangements in the gene trees were performed using an edge weight threshold of 98 (ultrafast bootstrap value). The reconciliation was then performed using the DTL (Duplication‐Transfer‐Loss) model with duplication, transfer, and co‐divergence costs of 1.5, 1, and 0, according to Wisecaver et al. (2014). To calculate number of transition (Ti) and transversion (Tv), 18 ECM protein family members were aligned separately using clustalW in MEGA 6. We determined the number of substitutions, silent, nonsilent mutation in each transition, and transversion category in which sequence with maximum similarity was used as masters (Belfield et al., 2012; https://www.hiv.lanl.gov/content/sequence/HIGHLIGHT/highlighter_top.html?sample_input=1&choice=mismatches&base=aa).

## 3 RESULTS

### 3.1 ECM‐associated evolutionary changes and translational and post‐translational events

**FIGURE 1:**
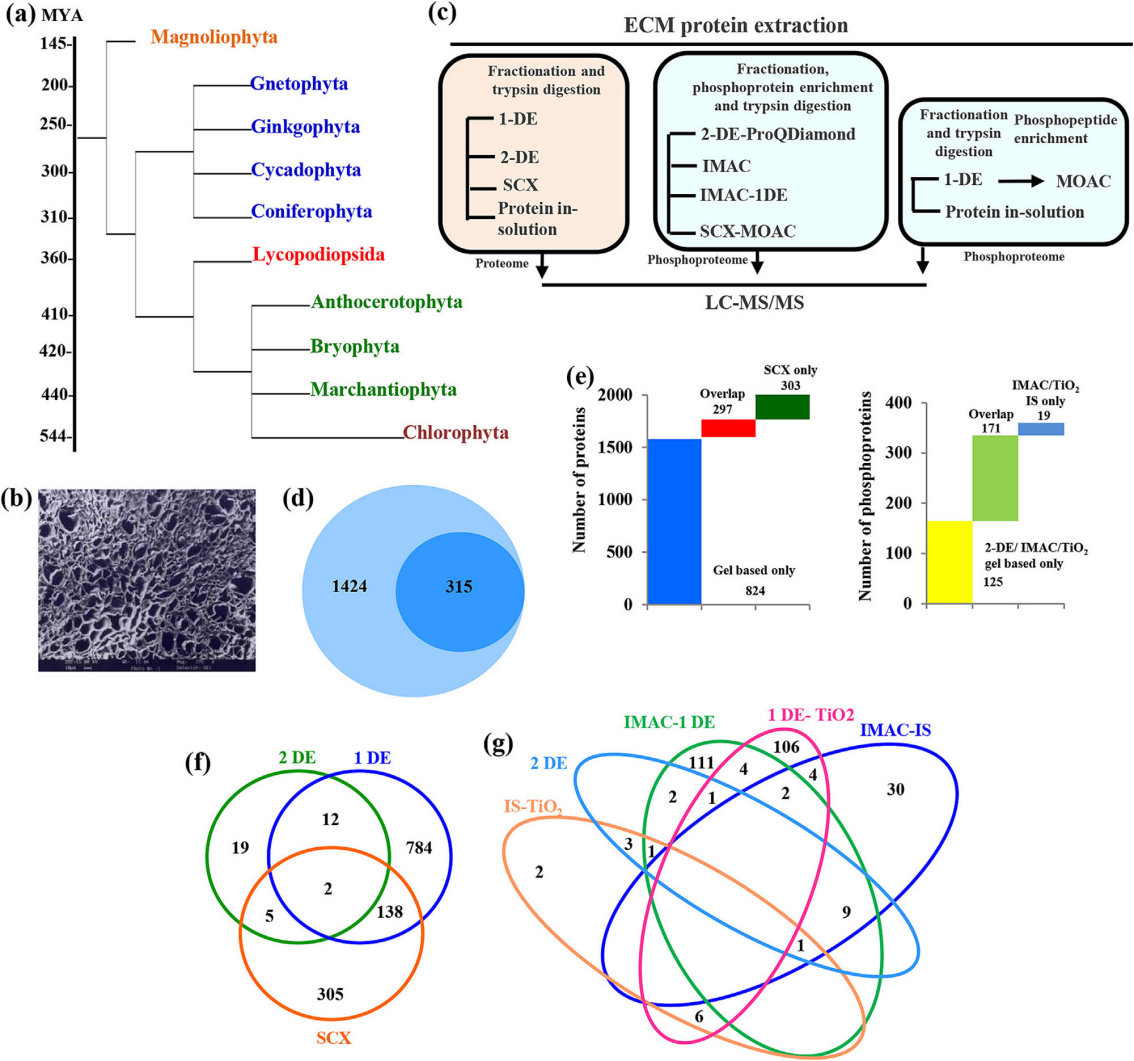
Analysis of plant ECM. (a) Phylogenetic analysis of land plants and ECM‐associated evolutionary changes. Tree is derived using a maximum likelihood approach. (b) Scanning electron microscopy of ECM. (c) Experimental workflow for the identification of chickpea ECM proteoforms. (d) Circle‐in‐circle diagram of unenriched and enriched ECM proteins. (e) Distribution of number of ECM proteins and phosphoproteins based on proteomic approaches. (f) Venn diagram showing number of ECM proteins identified using 1‐DE, 2‐DE, and SCX fractionation. (g) Comparison of methods for phosphoprotein enrichment. Venn diagram showing number of ECM phosphoproteins identified using in‐solution TiO2, 2‐DE, IMAC‐1DE, 1‐DE‐TiO2 enrichment, and in‐solution‐IMAC enrichment.

Phylogenetic analysis revealed that emergence of land plants that exhibited simple leafless growth axes (telomes) appeared to be dated back to 470 million years (MYA) ago, and monophyletic groups of tracheophytes were diversified to Magnoliophyta at around 145 MYA (Figure 1a). Based on plant phylogenetic position, ECMs are variable in their structure, composition, and developmental stage. ECM of Magnoliophyta contributed to its multi‐environment adaptation during million years of evolution. Fabaceae is a cosmopolitan family in Eudicotyledon adapted to land. In this context, ECM proteoforms in Fabaceae family member belonging to Rosid clade, namely, *C. arietinum* (chickpea), is of evolutionary as well as biological significance. SEM revealed Type I ECM in chickpea, which is composed of a microfibrillar carbohydrate‐protein meshwork (Figure 1b). To identify diverse matrix proteoform families, ECM fraction was isolated by mechanical disruption, proteins were extracted, and purity analysis of ECM fraction was performed using TEM (Bhushan et al., 2006). Micrographs showed that ECM fraction was free from other organelles and had no intact cells. Organelle specific marker enzyme assays revealed that ECM protein extract neither showed significant cytoplasmic glucose 6 phosphate dehydrogenase and peroxisomal catalase activity nor had plasma membrane associated vanadate‐sensitive H^+^ ATPase activity (Figure S1). Thus, it can be concluded that ECM proteins did not show cytosolic, peroxisome, and plasma membrane protein contamination.

To further substantiate the ECM proteome of chickpea, we performed comprehensive cataloging and comparison of ECM protein families by 1‐DE, 2‐DE, and SCX fractionation followed by mass spectrometry (Figures 1c,d and S2a). In total, 1,121 ECM proteins were identified by 1‐DE and 2‐DE (Figure 1e). However, certain comigrating proteins and highly abundant protein obscured identification of low‐abundant strongly bound matrix proteins eluted at low pH. To overcome this, SCX fractionation was performed coupled to mass spectrometry. Utilizing all three techniques, 1,221,324 MS/MS spectra were acquired and assigned to 1,424 ECM proteins (Figure 1d). Among them, 138 cell wall proteins (CWPs) overlap between SCX fractionation and 1‐DE, whereas 12 proteins were shared between 1‐DE and 2‐DE. Moreover, 784, 19, and 305 proteins were exclusively identified from 1‐DE, 2‐DE, and SCX‐MS/MS, respectively (Figure 1f; Table S1). These data served as the foundation for our investigation of ECM protein diversification across other taxonomic species.

To obtain a first global ECM phosphoproteome of chickpea, we next used phosphoprotein/peptides enrichment coupled with mass spectrometry in a combinatorial manner to analyze the phosphorylation status of matrix proteins (Figures 1c and S2a). First, we detected ECM phosphoproteins using 2‐DE stained with Pro‐Q Diamond dye and identified 14 phosphoproteins lacking phosphorylation motifs and sites. Thus, second approach was used to resolve ECM proteins on 1‐DE, trypsin digested, and pooled peptides were subjected to TiO_2_ phosphopeptide enrichment, and 123 phosphoproteins were identified using this approach (Figure 1g; Table S2). To further increase coverage, ECM proteins were in‐solution digested and peptides were separated by SCX fractionation followed by TiO_2_‐ phosphopeptide enrichment and LC‐MS/MS analysis. To catalog more matrix phosphoproteins, in‐house prepared gallium‐based IMAC phosphoprotein enrichment technique was adopted and enriched phosphoproteins were subjected to 1‐DE followed by LC‐MS/MS. In total, 134 phosphorylated matrix proteins were identified in this approach. Further, 58 phosphosites were identified using phosphoenrichment and in‐solution trypsin digestion followed by LC‐MS/MS analysis (Figure 1g; Table S2).

To understand biological functions of identified ECM proteoforms, we performed Blast2GO analyses by mapping and annotation of GO terms into two categories (Figure S2b,c). In biological process category, proteoforms were enriched for metabolic and cellular processes and response to stress corresponding to the pleiotropic nature of ECM. According to molecular function category, the predominant ECM function was binding, including hydrolase activity, ion binding, and oxidoreductase activity. A set of proteins found to be associated with CWI maintenance system was segregated into five categories: (a) cell wall biogenesis, architecture, development, and remodeling; (b) redox homeostasis and transport proteins; (c) signaling and transcription regulation; (d) protein folding; and (e) innate defense related proteoforms. These simple or large multidomain protein groups might function as remodelers, anchors, scaffold, and defense regulators. In addition, proteins of unknown functions were also detected in this study (Figure S2d,e).

### 3.2 Assembly, annotation, and organ level resolution of plant matrisome across lineages

To define the global plant matrisome, we screened available 74 ECM proteomes of angiosperms, gymnosperm, pteridophytes, bryophytes, and algae (Table S3). Thus, matrisome is defined as the collection of ECM‐associated proteins from available ECM proteomes. We curated ECM protein lists based on structural or functional features that include ECM‐affiliated proteins, sensors, regulators, and remodelers. In silico analysis of matrisome relied on examination of protein database UniProt, TAIR, GIGA DB, wallprotDB, and InterPro. Plant matrisome having 6,833 ECM proteins were assembled into nine functional classes that included structural proteins (1.4%), signaling (1.2%), proteins with interacting domains (7.9%; LRR domains and enzyme inhibitors), proteins related to lipid metabolism (3.5%), proteins acting on wall polysaccharides (26%; hydrolases, esterases, lyases, chitinase, and expansins), proteases (9.1%; proteinase, amidases, and peptidase), oxidoreductase (15%; peroxidases, oxidases, and berberine‐bridge enzymes), proteins of miscellaneous (7%) and unknown (29%) functions. Largest class of unknown function was further categorized into CW‐known unknown (22%), domain of unknown function (DUF; 1.6%), and protein of obscure function (POF; 5.4%; Table S4).

We further exploited plant matrisome atlas at organ level from Phanerogamia and Cryptogamia that includes roots (1,178 CWPs); leaves (1,527 CWPs); stems (2,093 CWPs); suspension cultures (1,032 CWPs); protoplasts (24 CWPs); etiolated hypocotyls and seedlings (707 CWPs); fruit pericarp, inflorescence, cuticle, berries, and seeds (2,009 CWPs); and whole seedlings (2,592 CWPs). About 5,532 matrix proteins were reported from 24 Dicotyledonae families, comprising 19 Eudicots and 5 Asterides majorly from taxonomic order Fabales, Brassicales, Solanales, Malphigales, Malvales, and Sapindales. Plant matrisome atlas encompasses 1,352 CWPs from four families and three taxonomic orders of Monocotyledonae; key contributor was Poales. Six and 22 CWPs were enlisted from a Gymnospermae *Pseudotsuga menziesii* of Pinaceae family and bryophyte *Physcomitrella patens* belonging to Funariaceae, respectively. Also, two Chlorophyta algae ECM proteins were identified from *Closterium peracerosum*.

### 3.3 Systematic prioritization of CWI proteins in organ and taxonomic clade network and their diversification across plant lineages

**FIGURE 2:**
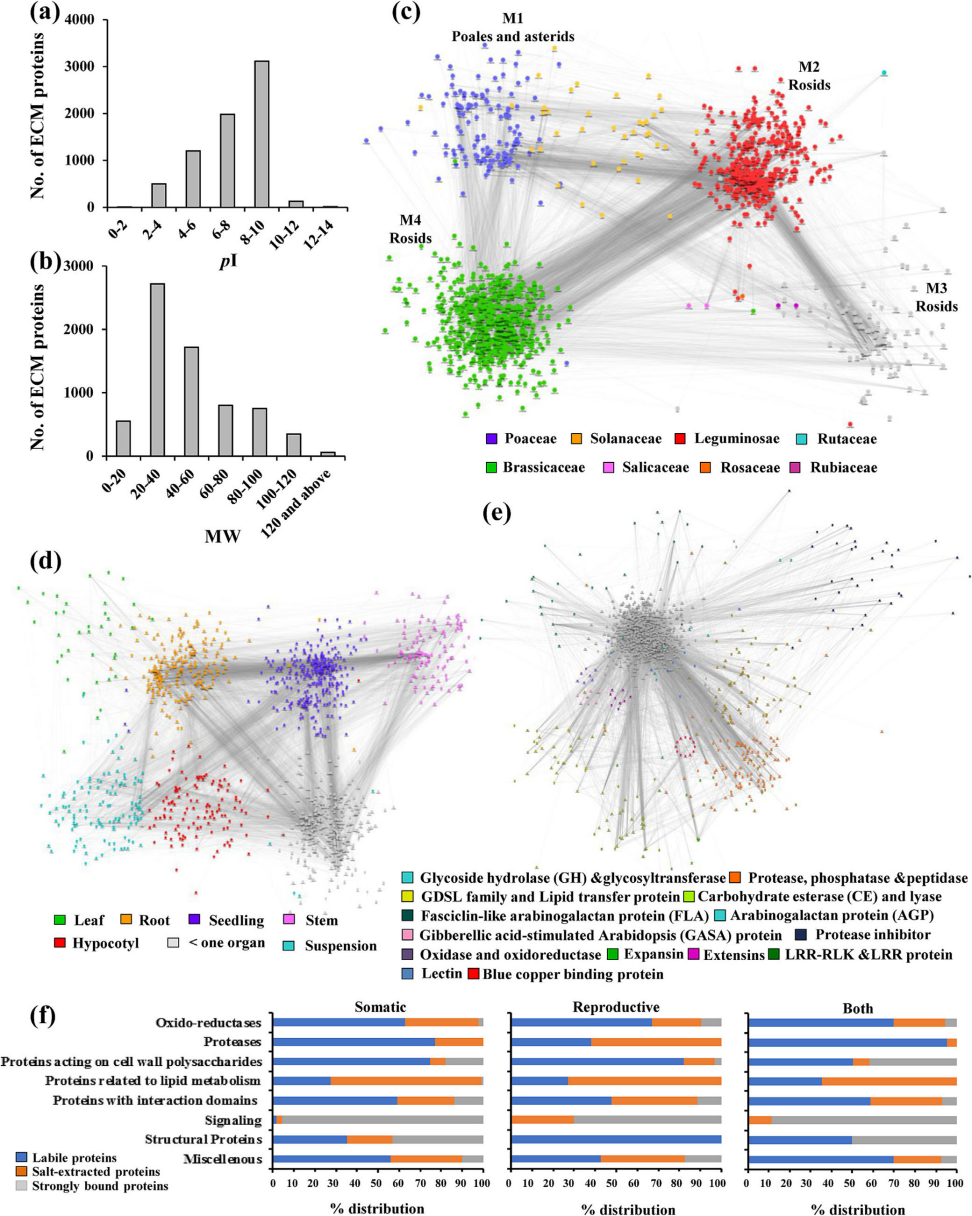
Development of plant matrisome. (a) pI. (b) Molecular weight (MW) of ECM proteins. Network analysis of matrix proteins. (c) Taxonomic family‐based matrisome network of eight families. The four modules represent one family. Gray lines represent conservation between two modules from different families. (d) Network relationship between matrix integrity proteins from different organ/cell suspension. Node represents organ associated ECM protein based on top BLAST hit against SwissProt, and edge denotes probability of two (nodes) potentially interacting based on cytoprophet algorithm. Comparative assessments of plant matrisome proteins across organs. (e) ECM protein families integrative network analysis showing functional protein modules. Nodes and edges represent ECM proteins and correlation between proteins, respectively. (f) Distribution of identified matrix proteins from somatic organ, reproductive organ, and from both.

We then assessed commonalities and differences between matrix proteins from different organs in a species or families. Three types of matrix proteoforms can be distinguished in plant matrisome based on their interactions with ECM components. Acidic labile proteins having little or no interactions (655 CWPs), basic salt‐extracted weakly bound proteins (3,182 CWPs), and strongly bound proteins (3,115 CWPs) (Figure 2a; Table S4). Wall integrity components were found to be varied in all three categories. Molecular weight (MW) analysis depicted that 6,401 CWPs were of MW between 20 and 156 kDa related to mechanic and semantic properties of ECM (Figure 2b; Table S4). These matrix proteins were probable remodelers, anchors, and architectural proteins that might be related to wall integrity of different plant families. Data suggested that matrix integrity proteoforms exhibited similarity among Phanerogamia and Crypogamia. Taxonomic family‐based matrisome network comprising eight families, including Monocotyledonae and Dicotyledonae, highlights the role of interaction mechanisms and selection pressures for adaptation in matrix proteins among lineages. Data depicted that module1 (M1) comprising matrix proteins of Poaceae (Poales) and Solanaceae (Asterids) interacted with Rosid clade in Modules 2 and 3, confirming the polyphyletic origin of taxonomic families and probable subfunctionalization and neofunctionalization of wall integrity component (Figure 2c). Matrix proteins mainly existed in hubs, which included carbohydrate degrading enzymes, proteases and structural proteins. Commonality in segregated hubs is multidomain and complex structural attributes of proteoforms and diversity existed in aminoacid sequences, and physicochemical properties (Figure 2a–c).

Next, we focused on relationship between matrix integrity proteins reported from different organ/cell suspension with adequate sample size in plant matrisome (Figure 2d). Some hub proteins defined in different organs modules showed overlap due to their biophysiological similarity (Figure 2d). Matrix integrity proteins that exhibited common themes across organs included organic acid biosynthetic processing, elongation, and translation regulation and multiple lipids metabolic process. Besides commonality, there were specific themes for each organ in diverse lineages. Hubs related to stem and leaves showed overlap of noncannonical ECM‐associated metabolic proteins and cell–cell communication proteins. However, leaf‐associated distinct matrix integrity proteins involve components of developmental signaling, hormonal response, and adaxial‐abaxial polarity determinants. Stem displaying eustelic or atactostelic organization had distinct ECM integrity proteins related to cell fate decisions, development, vasculature differentiation, and stress response. Interestingly, roots exhibited features, including gravitrotic response, water‐mineral transport, and anchorage. Distinct matrix integrity protein related to root development, perception, ion transport, and patho‐symbiotic interactions was reported across lineages (Figure 2d; Table S4). Matrix integrity proteins identified from cell suspension pointed that oxido‐reductases were predominant because of mechanical and oxidative stress. An investigation of hubs related to cotyledon, a first aerial organ to differentiate depicted that leaf and cotyledons share similarity in matrix integrity proteins related to organ expansion and storage, diversity existed in development related proteins, among lineages (Table S4).

Following this, we developed ECM protein family network from eight families, which were identified in the matrisome of somatic organ, reproductive organ, and both. Glycoside hydrolase and glycosyltransferase family showed maximum interaction having 239 nodes, followed by protease, phosphatase, and peptidase family (132 nodes), GDSL family and lipid transfer protein (68 nodes), protease inhibitor (28 nodes), fasciclin‐like AGP (20 nodes), carbohydrate esterase and lyase, (18nodes), AGP (12 nodes), and gibberellic acid‐stimulated arabidopsis protein (10 nodes), respectively (Figure 2e). Oxidoreductases in ECM might regulate transition from somatic to reproductive phase. Oxidoreductase isoforms were reported in both somatic (525 CWPs) and reproductive (314 CWPs) organs and network‐based interaction analysis revealed that the module was centered around berberine‐bridge oxido‐reductase, multicopper oxidase, blue copper binding protein, and laccase confirming the importance of redox regulation in phase transitions and wall integrity. It is observed that in somatic organs labile proteases (322 CWPs of total 432 CWPs) were predominant compared to salt‐extracted proteases (52 CWPs of total 89 CWPs) from reproductive organs (Figure 2f). Network analysis pointed that protease module mainly encompasses eight major proteases, namely, asp/ser/cys protease, ser carboxypeptidase, peptidase M20, C26, S28, and M28, suggesting that they contribute to rapid turnover of matrix protein at varied p*I* in same or diverse plant families. Interestingly, matrix integrity proteins acting on wall polysaccharides are represented in all organs. However, strongly bound proteins (242 CWPs) were identified from somatic tissues probably for providing mechanical strength by crosslinking carbohydrate moieties. In silico interaction analysis showed that isoforms of glycosyl hydrolase (GH), carbohydrate esterase (CE), polysaccharide lyase (PL), glycosyl transferase (GT), expansin, and PNGase A interact among themselves and to proteins related to lipid metabolism, facilitating ECM‐cell membrane continuum to regulate communication between cell components in diverse lineages. Network analysis also suggested that GDSL family and lipid transfer protein (LTP) interacted with pectinmethylesterase inhibitor (PMEI), protease inhibitor, LRR protein, and lectin, pointing that macromolecular assembly of carbohydrate, lipid, and protein in ECM regulate wall integrity and multicellularity of plants. Presence of salt‐extracted and strongly bound matrix integrity signaling proteins, particularly fasciclin‐like arabinogalactan protein (FLA) in both somatic and reproductive organ implied that ligand and ligand binding features were predominant across lineages. Interaction between CWI signaling components, including LRR‐RLK, LRR‐extensin (LRX), extensins, proline‐rich protein (PRP), COBRA‐like protein, and glycine‐rich protein (GRP) was also observed in the analysis.

**FIGURE 3:**
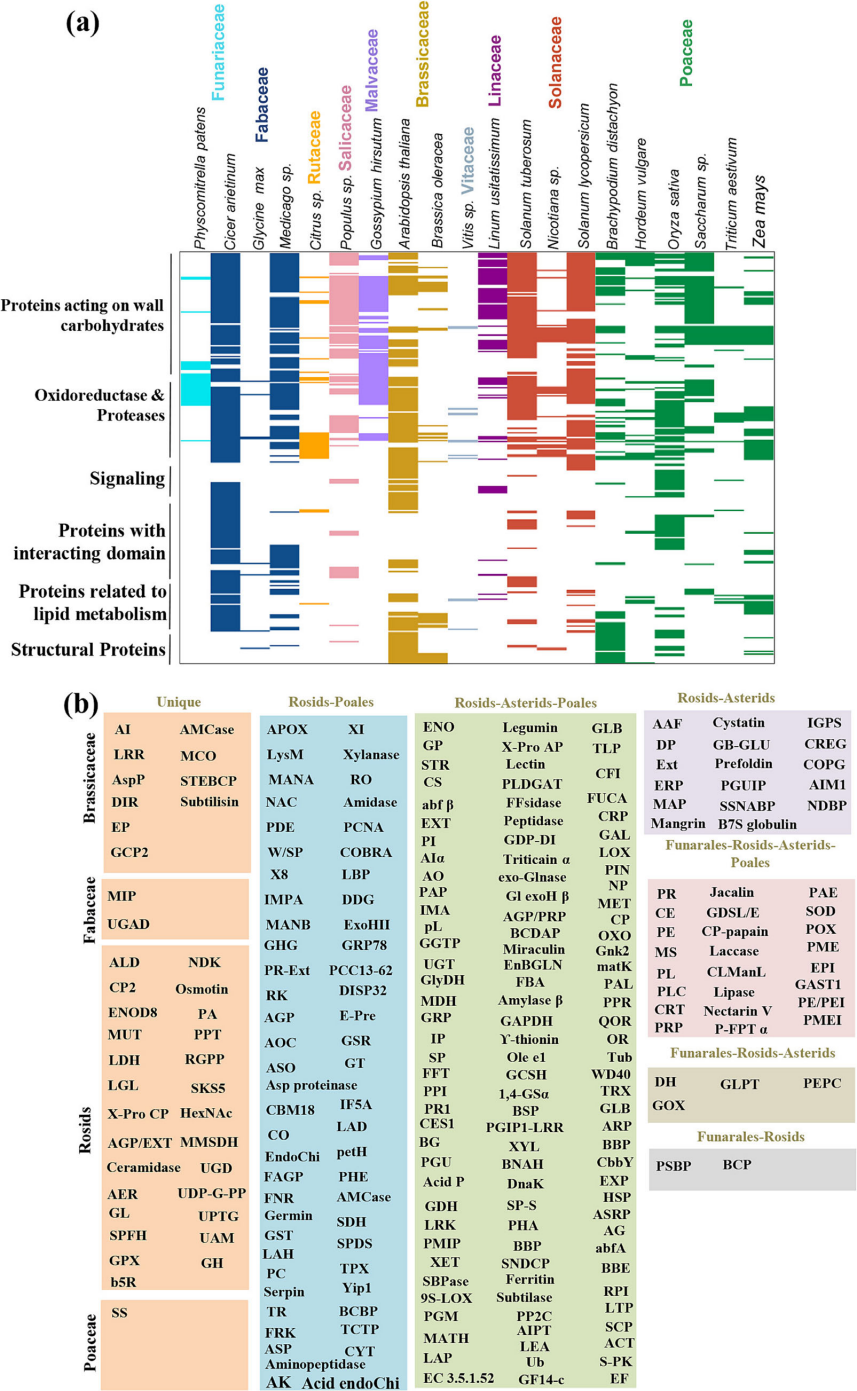
Conservation of the different functional classes of plant matrisomal protein described in meta‐analysis. (a) Conservation is shown as the presence/absence of a horizontal line in 20 different species belonging to Funariaceae, Fabaceae, Rutaceae, Salicaceae, Malvaceae, Brassicaceae, Vitaceae, Linaceae, Solanaceae, and Poaceae. (b) Unique and shared plant matrisomal protein across different taxonomic families and orders based on the meta‐analysis of available ECM proteomes.

Furthermore, in‐depth clustering of matrix integrity proteins among diverse taxonomic families revealed that few of carbohydrate modifying enzymes acting on glucose and arabinose such as UDP‐glucose:protein transglucosylase, UDP‐arabinopyranose mutase, and glycosyl transferase family were specific to Brassicaceae, Fabaceae, Rosids, and Poaceae. However, carbohydrate modifying enzymes acting on glucose, xylose, xylulose, galactose, ribose, and arabinose such as arabinofuranosidase beta, Glucan Exohydrolase beta, 1 4‐glucan‐protein synthase alpha, xylanase, polygalacturonase‐inhibiting protein, and Ribose‐5‐phosphate isomerase were common in Funarales, Rosids, Asterids, and Poales consistent with the diversity of wall composition in plant kingdom (Figure 3a,b; Table S4).

### 3.4 Relationship between evolution of plant kingdom, matrix diversity, and annotation of orthologous clusters across plant families at the proteome scale

**FIGURE 4:**
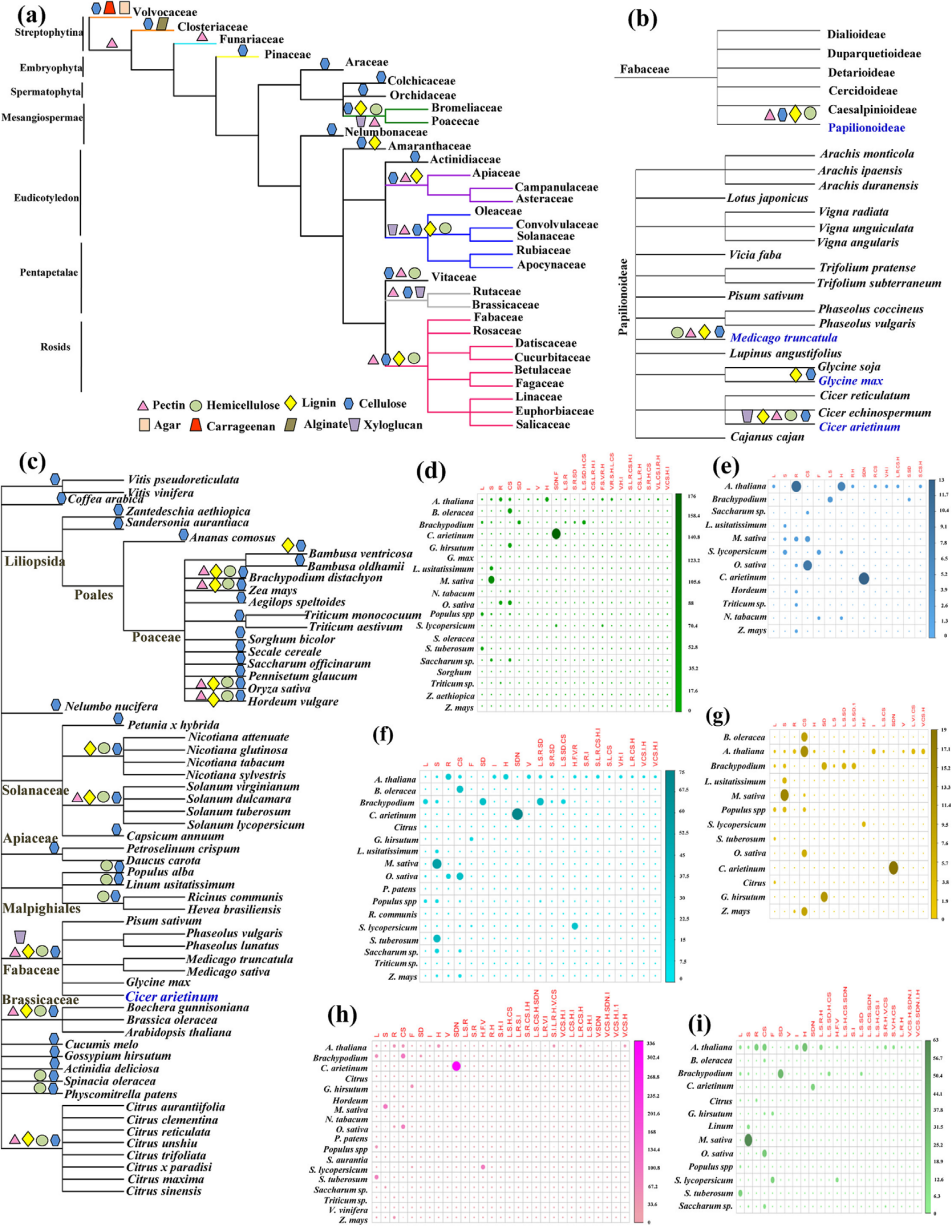
Plant phylogeny highlighting wall components. (a) Taxonomic family tree showing proteins‐polysaccharides biosynthetic machinery. Tree is derived from maximum likelihood approach. (b) Phylogenetic changes in matrix composition mapped onto Fabaceae family. Phylogenetic relationship among 21 Papilionoideae members. (c) Phylogeny of species based on wall composition using matrix proteins showing one‐to‐one orthology among species. Branch lengths are drawn to scale. Expansion of plant ECM protein families. (d) Oxidoreductase, (e) structural protein, (f) protease, (g) signaling, (h) proteins acting on wall carbohydrates, and (i) protein with interacting domain. Row represents species and column represents organs. Organ names are in red, and ECM protein families are in different colored corrplot. Color intensity and size of circles are proportional to number of protein in family. Abbreviation: CS, cell suspension; F, fruit; H, hypocotyl; I, inflorescence; L, leaf; L‐S‐R(V), vegetative; R, root; S, stem; SD, seed; SDN, seedling.

We next investigated the relationship between differences in wall composition and evolution of lineages. Nine matrix protein groups were examined by manual and computational screening to understand their distribution pattern and evolutionary significance in different organs and families. It was observed that many classes of matrix proteins‐polysaccharides and their biosynthetic machinery appear to be highly diverse and vary across clades, families and species, and its expansion resulted in large multigenic families associated with CWI (Figure 4). As expected, cellulose and pectin represent the ubiquitous ECM components of the plant lineages. Carrageenan, agar, and alginate were found to be exclusively present in Streptophytina. In both Streptophytina and Embryophyta, pectin‐rich portions of wall have a role in cell–cell attachment and may share some characteristics of cell–cell communication favoring multicellularity (Figure 4a). Matrisome screening of Papilionoideae members in Fabaceae family showed that only three species (*M. truncatula*, *G. max*, and *C. arietinum*) are explored at the proteome level, whereas genome of 21 members is available till date. Phylogenetic analysis of 21 Papilionoideae members and mapping of matrix protein‐polysaccharide biosynthetic pathway components revealed that wall composition diversity was probably due to protein diversification, mutations, and duplications followed by selection (Figure 4b). Next, we analyze phylogenetic relationship between plant species belonging to Rosids, Asterids and Poales with available matrix proteome and found that cellulose biosynthesis in ECM appears to be ubiquitous among members. Till date, very few members of cellulose metabolism (four proteins) were identified in many of the plant ECM proteome, indicating the low coverage of plant matrisome (Figure 4c). Wall integrity remodelers showed wide‐spread distribution in matrisome, including 564 oxidoreductases from somatic and reproductive organs of Rosids, Asterids, and Poales (Figure 4d). Seventy‐two strongly bound structural proteins, 138 signaling proteins, and 80% of proteins related to lipid metabolism were cataloged from somatic organ/cell suspension probably act as integrity sensors or signal transducers. With experimental coverage of 23%, matrix proteins acting on wall carbohydrates were found to be the most abundant, and 787 matrix proteases were distributed across 17 species in at least one organ/suspension. Protein with interacting domain is another important functional class in the matrisome having 356 ECM proteins, 31% of which was from reproductive organs and might involve in plant development across lineages (Figures 4e–i and S3). Miscellaneous ECM proteins that are non‐canonical represent 6% of matrisome, including glycolytic enzymes, transcription factors, germin, and dirigent protein identified from varied organs. Finally, matrisome included CW‐known unknown proteins (28%) with known functionality in biological process but lacking experimental evidence demonstrating function in cell wall biology was distributed in 54 species from Rosids, Asterids, and Poales. In‐depth investigation of matrisome revealed 98 domains of unknown functions (DUFs) in stem or cell suspension culture of Phanerogamia (18 and 80 from Monocotyledonae and Dicotyledonae, respectively), which is highest till date. Further, 205 proteins of obscure function (POF) lacking recognized domains were identified in both Phanerogamia and crypogamia (Figure S3). The presence of cell wall‐unknown protein repertoire is not the most parsimonious hypothesis and should be subjected to periodic revision in light of future discoveries.

**FIGURE 5:**
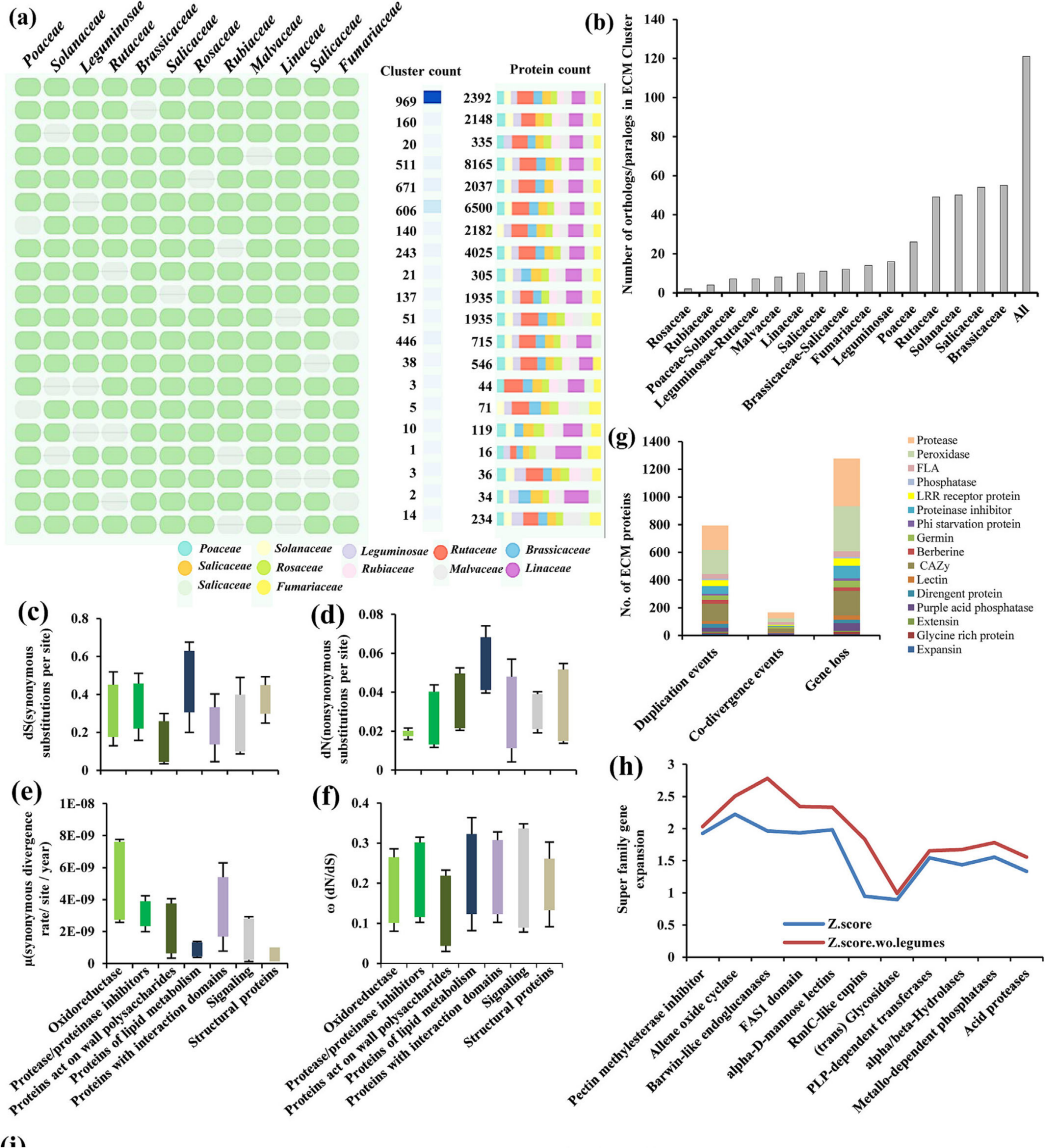
Comparison of orthologous cluster of ECM protein families. (a) Orthologous clustering analysis to identify putative orthology and paralogy. Cell graph depicted occurrence pattern of shared orthologous groups, namely, Poaceae, Solanaceae, Leguminosae, Rutaceae, Brassicaceae, Salicaceae, Rosaceae, Rubiaceae, Malvaceae, Linaceae, Salicaceae, and Fumariaceae. Pattern to the left indicates species in the clusters, each row represents an ortholog cluster group, and each column indicates a species. A green cell indicates the presence of a cluster group in the corresponding species, and a gray bar denotes the absence of a cluster group in that species. Cluster count represents number of clusters shared between species, and protein count denotes number of protein members in shared clusters. (b) Number of the ortholog and in‐paralog clusters. Analysis of the sequence divergence variation. (c) Boxplots showing differences in number of synonymous substitutions (dS). (d) Nonsynonymous substitutions (dN). (e) Absolute rate of silent site divergence (μ). (f) Substitution rate ratio (ω), among ECM protein families. (g) Reconciliation analysis. (h) Gene expansion. (i) Determination of missense mutation and number of transition and transversion in sequences of ECM proteins.

In order to annotate orthologous clusters across diverse clades of plant kingdom, the proteomes of 72 organisms, including *C. arietinum*, were downloaded from UniProt. OrthoVenn was used for orthologous clustering to identify putative orthology and in‐paralogy relations from clusters of ECM proteins. Sequence similarities between all input protein sequences were calculated with an e‐value cut‐off of 1e^−5^. The analysis showed that 3,124 orthologous clusters were formed based on the protein sequences from the 12 families (Figure 5a). Of the identified clusters, 840 clusters were related to ECM proteins. The analysis showed that 121 clusters were shared by all 12 families, suggesting their conservation in the lineage after speciation. Additionally, it showed that 16 clusters were specific to Leguminoceae, including *C. arietinum* and regarded as in‐paralog clusters. The presence of the in‐paralog clusters suggested that there might be a lineage specific protein expansion in Leguminoceae (Figure 5b). Based on annotation of these clusters, some of these lineage specific clusters could be potentially involved in important biological processes related to ECM function such as response to stimuli, defense response, nitrogen compound metabolic processes, and wall signaling. These observations were consistent with the evolutionary analysis of plant matrisome of 72 organisms. Multiple sequence alignment (MSA) and motif analysis indicate that the ECM cluster had ECM organization and signaling associated proteins (Figure S4). Phylogenetic tree showed that in ECM cluster, Brassicaceae contained more paralogous proteins, suggesting lineage‐specific expansion of ECM protein (Figure S4). Further, ECM proteins from the same species had higher similarity to one another than proteins between different species, suggesting that duplication events after speciation resulted in closely related paralogous genes.

### 3.5 Analysis of the sequence divergence variation, patterns of gene gains, and loss through gene duplications, gene expansion, and inference of proportion of sites under selection

In order to identify targets of positive selection, we selected all 18 ECM protein families from matrisome and calculated dN:dS ratio to assess the evolutionary divergence of each protein family (Yang & Bielawski, 2000). dN: dS > 1 pointed towards the selective advantage to amino acid substitutions in a protein. Of the total 6,833 matrisome‐associated proteins from different families, 862 members showed a strong positive selection. We also concluded that all 18 ECM protein families had a higher proportion of positive and neutral sites followed by conserved sites and showed significant differences in the rates of sequence divergence across lineages (Table S5). The dS and dN of ECM proteins were lower in gymnosperms and algae than in angiosperms. Interestingly, the ratios of non‐synonymous/synonymous substitutions (ω) were comparatively higher in berberine, Carbohydrate‐Active enZymes (CAZy), extensin, fascilin AGP, LRR proteins, peroxidase, Phi starvation protein, proteinase inhibitor, purple acid phosphatase, and lectin compared to other ECM family proteins. We next build the dN/dS trees and determined the variation in selective pressures (ω) among stem branches in the different ECM protein families across lineages. It is hypothesized that terminal branches of tress had different omegas. Omega ratios for proteins acting on wall polysaccharides were higher than other ECM family proteins (Table S6; Figures S5–S9). When compared with pairwise estimations of omega for wall integrity protein, consistent results were obtained across lineages. However, no significant difference was observed in dN between angiosperms, algae, and gymnosperms for ECM protein families when compared with branch estimations for selection pressure.

The most significant differences were found in the absolute rates of silent‐site divergence (μ), with berberine, lectin, protease, proteinase inhibitors, extensin, and glycine rich protein rates being, on average, four or five times higher than that observed in Class 3 fascilin protein members. Differences in ω and μ were also significant among ECM protein families (Figure 5c–f).

Mapping of gene family trees on a species tree was performed within 18 ECM protein families. Gene duplication, loss, and co‐divergence events were inferred by reconciliation analysis with the species tree using the DTL (Stolzer et al., 2012). Several orthologs and paralogs due to duplication events were deduced from the phylogenetic tree with strong bootstrap support. Reconciliation analysis reported that proteases (176 duplication events and 344 gene loss), peroxidases (173 duplication events and 324 gene loss), and CAZy proteins (125 duplication events and 177 gene loss) have been marked by a remarkable number of gene duplication and gene loss events (Figure 5g). In addition to gene family gains and losses, gene family expansions also contribute to the evolution of species. Next, we compared the proteomes of 72 matrisome against the gene families from the SUPERFAMILY database to identify gene family expansions in ECM protein families. We found specific expansion events in Asterides, Rosids, and Poales. Z‐score value above 2 was observed in ECM protein families of *C. arietinum* and score increased further when the analysis was performed with all proteomes except legumes in SUPERFAMILY database confirming gene expansion of ECM protein families (Figure 5h). Further, it was reported that substitution rates of transitions are higher than transversions to conserve biochemical properties of the original amino acid (Lyons & Lauring, 2017). Our study also corroborated this finding and we observed higher number of transition events in ECM protein families (Figures 5i and S10–S16).

We further demonstrate a fitness difference in transition and transversion mutations using four deep mutational scanning data sets of influenza virus and HIV, which provided adequate statistical power. We find that three of the most commonly cited radical/conservative amino acid categories are predictive of fitness, supporting their utility in studies of positive selection and codon usage bias. We conclude that selection is a major contributor to the transition:transversion substitution bias in viruses and that this effect is only partially explained by the greater likelihood of transversion mutations to cause radical as opposed to conservative amino acid changes.

### 3.6 Evolutionary landscape and interaction analysis of wall remodelers and enzyme machinery

To study evolution and interaction of wall remodelers, we first analyzed matrisome for presences of carbohydrate degrading enzymes (CDEs), hydrolases, and pectin modifying enzymes (PMEs) in different clades across plant lineages (Figure S17a,b). We provided evidence that a complete set of enzymes involved in wall carbohydrate biogenesis was present in Embryophyta and β‐(1,4)‐glucan synthase isoforms were detected in *Physcomitrella*. CDE/modifying protein aminoacid sequences from matrisome were used to perform BLAST searches and identified 179 CAZy. We found two evolutionarily related clusters having 29 and 41 sequences of GH sharing common origin, implying that first GH gene duplication occurred before its predominance in land plants. Similarly, CAZy and PMEs were detected in major lineages exhibiting duplication events of the pro‐domain due to selective pressure during evolution. To unravel interactions among CDE/modifying proteins, we applied co‐occurrence network analysis to resolve wall reorganization interaction networks, which encompass 207 nodes having role in wall architecture remodeling, hydration, modification, and acidification. The most interactive protein was GH with galactosidase and pectinesterase, confirming the fact that carbohydrate modification is linked with wall integrity and organization.

**FIGURE 6:**
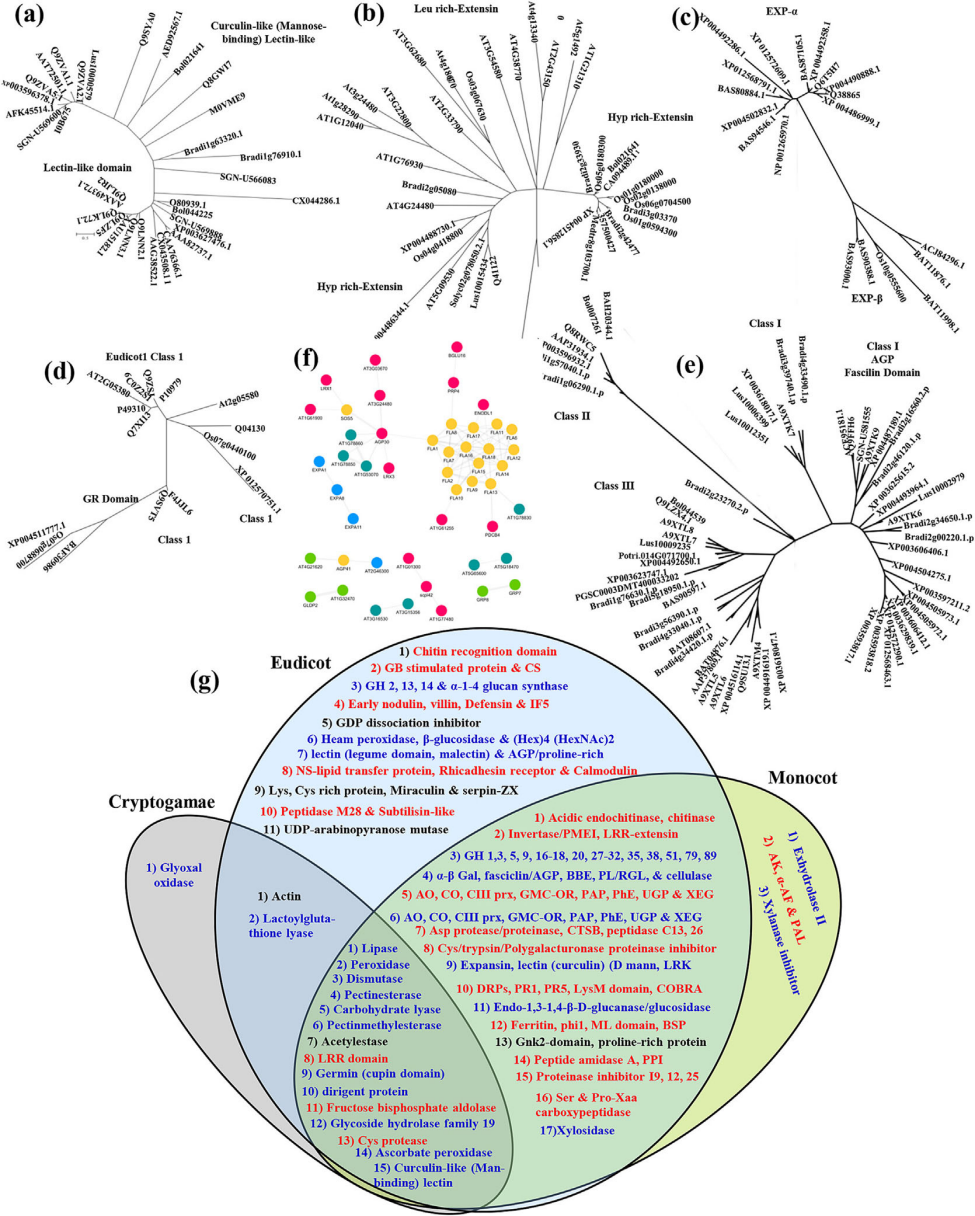
Defining evolution and interaction of wall integrity signal components in plant matrisome. Evolutionary relationship of (a) lectin. (b) Extensin. (c) Expansin. (d) Glycine rich protein. (e) Fascilin arabinogalactan proteins. (f) Network analysis depicting correlation among identified wall integrity signal components. Each node represents ECM protein based on top BLAST hit against SwissProt and an edge denotes a probability of two proteins (nodes) interacting based on the cytoprophet algorithm. Nodes and edges represent ECM proteins and correlation between proteins, respectively. (g) Venn diagram of the proteins identified from the metaanalysis of 72 ECM proteomes. Proteins in red, blue, and black are related to innate defense, ECM biogenesis and architecture, and signaling, respectively. Abbreviations: AGP, arabinogalactan proteins; AK, adenosine kinase; AO, amino oxidase; BBE, berberine branched enzyme; BSP, basic secretory protein; CS, Chalcone synthase; CO, cyclase oxidase; GH, glucosyl hydrolase; GB, gibberellins; GH, glycoside hydrolase; LRK, lectin receptor kinase; PAL, phenylalanine ammonia‐lyase; PAP, purple acid phosphatase; PE, pectinesterase; PhE, phosphoeaterase; PL, pectinlyase; PPI, peptidyl‐prolyl cis‐trans isomerase; prx, peroxidase; UGP, UDP‐glucose pyrophosphorylase; XEG, xyloglucan endotransglucosylase/hydrolase; α‐AF, α‐arabinofuranosidase; α‐Gal, alpha‐galactosidase; α‐Mann, alpha mannosidase; β‐Glc, beta‐glucosidase.

Comparative phylogenetic analysis and MSA of another wall remodeler, lectin cataloged in matrisomes of 10 angiosperms belonging to Brassicaceae, Fabaceae, Solanaceae, Rutaceae, Linaceae, and Poaceae demonstrated multi‐domain rearrangement of lectin protein during evolution. Curculin‐like (Mannose‐binding) lectin families evolved to be a separate clade in phylogeny, while others shared a closer evolutionary history (Figure 6a). Further, evolutionary relationship of expansins from diverse species of same clade demonstrated its segregation into exp‐α and β. Exp‐α was majorly identified from roots, while exp‐β mainly found in seedlings or stem, indicating that CWI is maintained by diverse wall machinery in different organs. Next, extensin with evolutionary conserved functional motifs was grouped into three clades, encompassing LRR extensin, Eudicot Hyp‐rich extensin, and Monocot hyp‐rich extensin. Data depicted that eudicot extensin epitomize specificity in reproductive and somatic organs. The study also pointed that tracheophytes encode extensins with repeated SP3‐4(SP3‐4SP3‐4) motifs; however, moss lacks these motifs relying majorly on Hyp‐arabinosylation (Figure 6b,c). Class 1 GRPs were clustered in taxonomic family specific manner containing a signal peptide followed by (GGX)n repeats. We further investigated evolutionary relationships of 44 FLAs from Rosids, Asterids, and Poales matrisome and identified four FLA clusters, namely, Class I, I domain, II and III. Class I was majorly clustered in phylogenetic tree and Q8L9T8/B0I035484/Potri005g167800 might have independent evolutionary trajectory compared with others clades (Figure 6d,e). Network modules revealed that remodelers interacted more significantly among same multigene family members than with different family, indicating that wall remodelers govern CWI signaling independent of one another (Figure 6f).

We also inspected evolutionary changes in wall enzyme machinery comprised of acid phosphatase (PAPs) involved in metal ligation. Matrisome and reports till date suggested that plant PAPs can be distinguished into small (35 kDa) and large (55 kDa) PAPs. PAPs were assembled into three phylogenetic clades, namely, Clade 1 predominated with small PAPs, Clade 2 related to Eudicot disulfide bridge large PAPs, and Clade 3 specific to Fabaceae disulfide bridge large PAPs, indicating that PAPs of Fabaceae evolve independently of other Eudicot PAPs. Evolutionary changes of wall‐associated oxidoreductase, berberine proteins (BBE) of Tracheophytes identified types I, II, and IV and not defined clade based on differences in active site. BBE with Type I active site first appeared in Eudicots. With evolution, active site type I was replaced by type IV and undefined BBEs were grouped with type 2 in same clade. Another matrix protein, germin, exhibits conserved sequence and structural similarities across kingdom. Germins of diverse Tracheophytes were grouped into four phylogenetic clades, namely, saccharum germin, germin 1, germin 3, and potato germin. Results indicated that germin evolved in plant families by segmental and tandem duplication and existed before Eudicot and Monocot diverges, while some expanded independently in species‐specific manner. Phylogenetic analysis of 248 PRXs revealed that segmental duplication events mainly contribute to its expansion across lineages. To address interaction among wall‐associated enzymes, we developed interaction network and observed that most of the oxidoreductases and hydrolases family proteins interact independent of one another (Figure S18a–e).

Clade‐specific evolution of peptidase inhibitors was observed in Tracheophytes, including Kunitz miraculin inhibitor, trypsin inhibitor, Kunitz inhibitors, and Solanum‐specific protease inhibitors. Analysis also indicated that proteases/peptidases are more conserved as compared to peptidase inhibitors in plant lineages. Subtilisin, aspartic protease, serine aminopeptidase, and prolylcarboxypeptidase were clustered in different clades, pointing towards independent evolution of peptidases/proteases in ECM. Network analysis revealed that proteinase inhibitors interconnect protease clusters, suggesting wide substrate spectrum of proteinase inhibitors. However, module containing proteases interact among themselves more closely than with protease/proteinase inhibitors family (Figure S19a–c). Finally, we analyzed the dirigent, phi, and LRR proteins that showed species‐specific diversification and evolution across clades (Figure S20a–d).

### 3.7 Functional characterization of ECM proteins across cryptogamia, Eudicot, and Monocot

The core ECM proteome profile was relatively different across the plant kingdom. However, consistency appeared in the functionality of ECM proteoforms (Figure 6g). As for Eudicots and Monocots, ECM biogenesis and architectural proteins such as peroxidases, glycosyl hydrolase, fasciclin AGPs, expansin, and lectin were the most abundant proteins, comprising 16.4% of ECM proteins. These proteins are known to be involved in wall carbohydrate rearrangement, degradation, or modification (Dehors et al., 2019). Despite this consistency, significant differences were noticed in proteins related to innate defense and signaling. Different isoforms of Asp protease/proteinase, catepsin B, peptidase C13, 26, Cys/trypsin/Polygalacturonase proteinase inhibitor, disease resistance proteins, PR1, PR5, LysM domain, COBRA protein, Ser, and Pro‐Xaa carboxypeptidase were identified, pointing towards the different molecular mechanism involved in response to external stimuli (Figure 6g).

Although the majority of the ECM proteins identified were shared between the angiosperms, we also found a number of proteins unique to each. Exhydrolase II, adenosine kinase, and α‐arabinofuranosidase were uniquely identified in Monocot ECM proteomes, whereas proteins with chitin recognition domain, GB stimulated protein, and chalcone synthase known to be involved in chitin mediated immunity were uniquely identified in Eudicots. Proteins that were significantly involved in antioxidant defense included haem peroxidase, β‐glucosidase, and (Hex)4 (HexNAc)2‐folate binding protein. Eudicot ECM also exhibited a greater abundance of proteins linked to signaling such as, Lys, Cys rich protein, Miraculin & serpin‐ZX, GDP dissociation inhibitor, and UDP‐arabinopyranose mutase. A number of oxidases, peroxidase, esterases, and several members of the heat shock protein family HSP70 were also in greater abundance in Eudicots, indicating that common molecular mechanism was involved in innate defense and antioxidant defense in different species of Eudicots. Interestingly, several proteoforms were found to be common among Cryptogamia, Eudicot and Monocots. The proteoforms, included isoforms of lipase, peroxidase, dismutase, pectinesterase, pectinmethylesterase, and glycoside hydrolase family 19, found to be involved in recognition, wall carbohydrate rearrangement, and degradation (Bradley et al., 2022; Guerra‐Guimarães et al., 2016; Jolie et al., 2010). Few proteoforms such as LRR domain proteins, Cys protease, and ascorbate peroxidase were associated with innate defense. The results showed that common structural/functional relationship existed in ECM of plant kingdom.

### 3.8 Matrix proteoform dynamics identifies novel phosphosignatures in wall‐associated events

**FIGURE 7:**
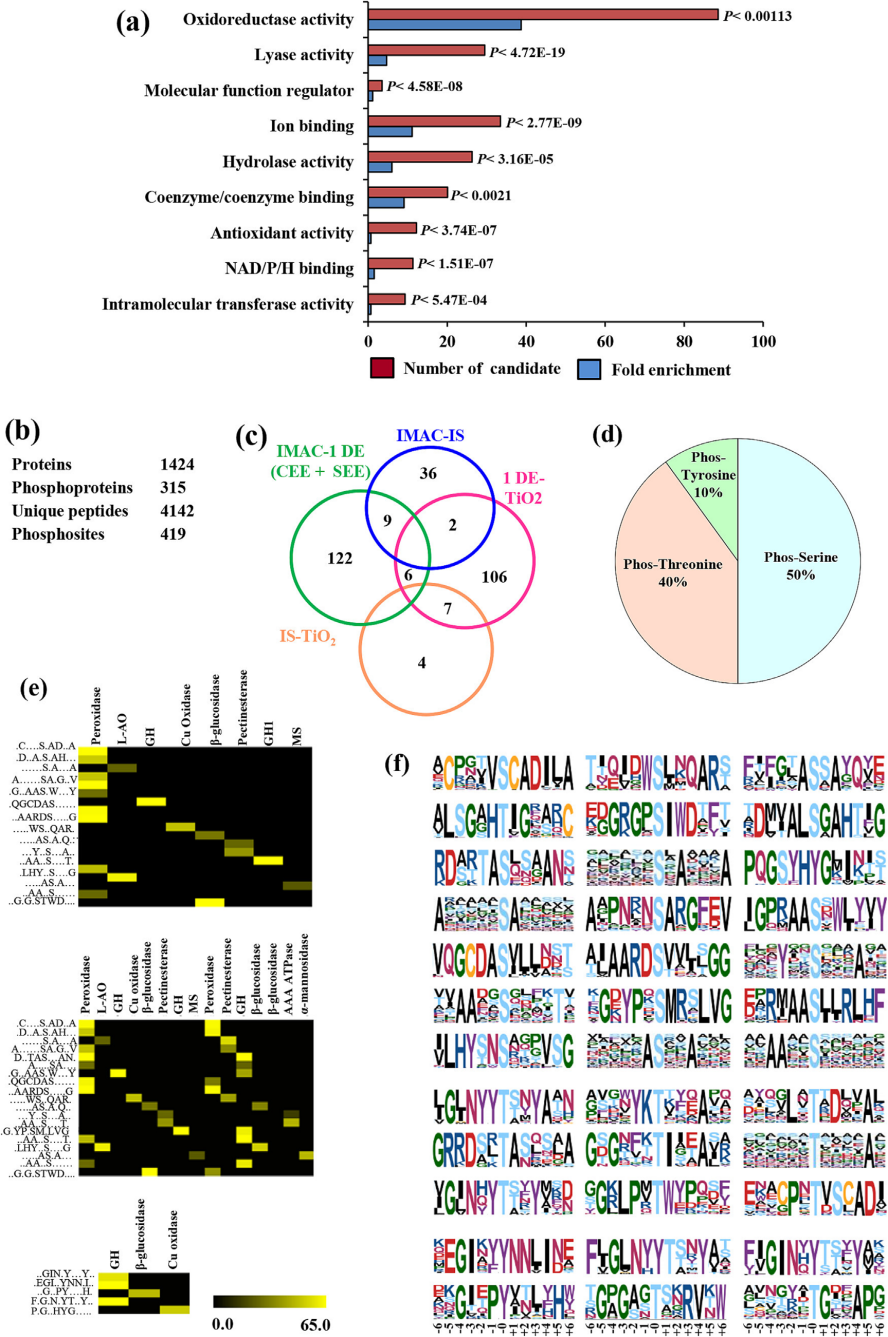
Analysis of chickpea ECM proteome. (a) Go enrichment analysis of phosphoprotein. Go‐enriched annotation of candidate phosphoproteome was compared with entire chickpea proteome. Each category listed is significantly enriched with *p* < .01. (b) Statistic on number of identified phosphopeptides. (c) Venn diagram showing overlap of identified. Phosphoproteins from different enrichment techniques. (d) Distribution of phosphorylated ser, thr, and tyr residues in the data sets. (e) Dynamic change in phosphorylation of sites matching the ser, thr, and tyr centered motif is color‐coded yellow. (f) Significantly overrepresented phosphorylation motifs were identified with Motif X and clustered.

To identify novel phosphosignatures that are activated during wall‐associated events, chickpea matrix phosphoproteins were analyzed using gene ontology (GO) annotations. Three‐hundred fifteen unique candidate phosphoproteins were examined for enrichment in biological process compared with the GO annotations of the proteins in the LegumeIPv2 (Figure 7a). Of the 21 known oxidoreductase‐containing wall proteins, 8 (38%) were identified using phosphoenrichment. For the other GO‐enriched components, about 2–10% were identified, representing NAD/P/H binding, antioxidant activity, coenzyme/coenzyme binding, ion binding, and molecular function regulator. For biological process, the candidate list enriched in oxidoreductases, transferases, and lyases were majorly involved in wall integrity, organization and biogenesis (*p* < .0001).

We identified 419 phosphorylation sites on 315 phosphoproteins that contained known and novel phosphosignatures (Figure 7b). Next, we estimated proportions of pS, pT and pY sites among phosphoproteins and searched for the occurrence of predominant phosphomotifs using Motif X. Phosphopeptide identifications from 43,124 MS/MS spectra revealed high proportion of serine and threonine phosphorylation sites (90%) and small number of phosphorylated tyrosine residues (10%) in chickpea matrisome atlas (Figure 7c,d). Further, we identified 17 significantly enriched phosphoserine, 13 phosphothreonine, and 5 phosphotyrosine motifs. We also examined phosphorylation motifs that might be involved in CWI signaling by clustering the motifs solely on the basis of change in S/T/Y phosphorylation status. Results showed that wall components, namely, matrix glycosyl hydrolase, beta‐glucosidase, and Cu‐oxidase, had all three S/T/Y phosphorylation motif and represent separate regulatory entities with distinct patterns of phosphorylation regulation (Figure 7e,f).

**FIGURE 8:**
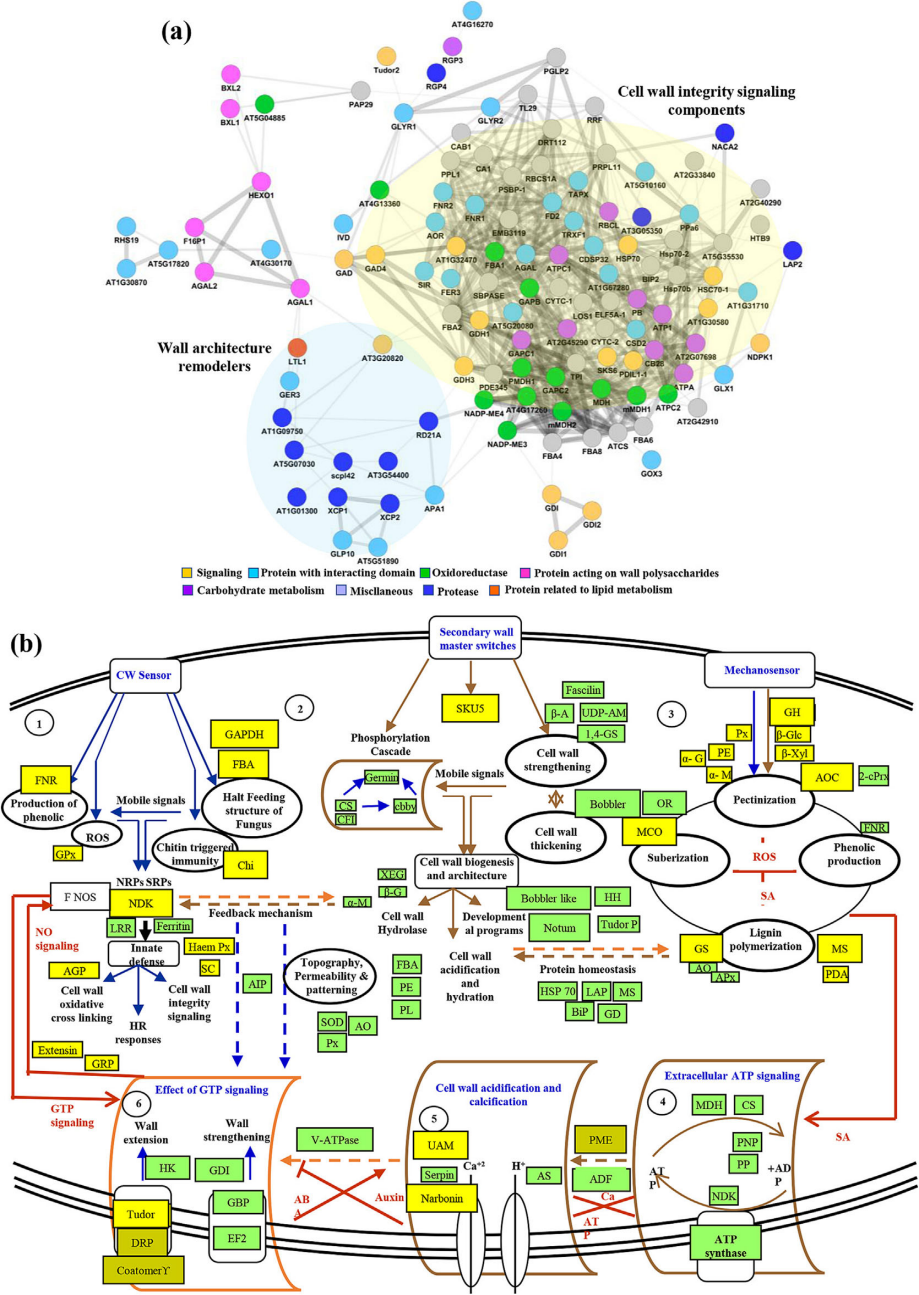
Functional correlation network and pathway analysis of identified proteoforms. (a) ECM proteoforms were subjected to partial Pearson correlation analysis visualized in Cytoscape. Node colors represent functional categories *p* < .01. (b) Model pathway representing wall integrity signaling. Identified proteins are in green boxes and phosphoproteins in yellow boxes. Abbreviations: AOC, Allene oxide cyclase; GD, glutamate dehydrogenase; GH, glucosyl hydrolase; GPx, glutathione peroxidase; MS, methionine synthase; PE, pectinesterase; α‐G, alpha‐galactosidase; α‐M, alpha mannosidase; β‐Glc, beta‐glucosidase; β‐Xyl, beta xylosidase.

Further, we performed combined protein–protein interaction network analysis of matrix protein and phosphoproteins (Figure 8a). Network encompasses 430 identified proteoforms that include 223 proteins, 107 phosphoproteins, and 99 common proteins. Proteoforms associated with wall sensor, biomechanical properties, and tensile strength had 132 proteoforms (62 proteins, 30 phosphoproteins, and 40 common). Most interactive protein was alpha‐1,4‐glucan‐protein synthase. Wall remodeling related module encompasses 14 proteoforms, and the most interactive protein was pectinesterase having role in wall hydration.

## 4 DISCUSSION

Comprehensive matrisome and meta‐analysis data presented here significantly expand our knowledge about wall‐associated events and evolutionary perspective of matrix proteins in diverse organs across plant lineages. Ecological niches and microenvironment within the organ/organism provide an excellent paradigm to investigate matrix proteoform dynamics. We used an agriculturally important legume, chickpea because of wide‐spread distribution of Fabaceae family, and its evolutionary significance in relation to other taxonomic clades, which were compared in the meta‐analysis. ECM exhibited pivotal role in epochal evolutionary event of tracheophyte, and its heterogeneity and sensing mechanism provides clues to proteoforms evolution that regulates its composition (Fangel et al., 2012; Kenrick & Crane, 1997). Matricellular proteins are the prime determinants of wall‐associated processes, lineage verdicts, and organ differentiation mediated by receptors, sensors, and ligands (Jamet et al., 2006; Humphrey & Bonetta, 2007). We found that receptor‐like kinases (RLK) involved in CWI sensing along with wall‐associated kinase, purple acid phosphatase, lectin, and GPI‐anchored‐cleavage proteins might sense, adhere, and modify matrix organization. We also set out to identify ECM sensory and structural components that include LRR‐extensins, topless protein, bobbler‐like tudor domain, and mannosyl‐endo‐β‐mannosidase having signaling or regulatory function during wall development (Diet et al., 2006; Schröder et al., 2009). Further, wall remodeling mediated by brassinosteroids, ethylene, jasmonate, gibberellin (GB), and auxin activates members of transcription factors through RLKs (Keyes et al., 1990; Polko et al., 2012; Sun, 2010; Tian et al., 2018). As reported earlier, our chickpea ECM proteome also suggested that brassinosteroid‐induced pectinlyase might alter pectin methyl esterification patterns regulating ECM sensory attributes (Shin et al., 2021). Wall assembly by endocytosis was evident in mutant emb30/gnom and double mutant of ADL1A and ADL1E dynamins with thickened walls by pectins internalization (Kang et al., 2003; Manfield et al., 2004). This supports our finding that matrix‐associated pectinesterase, pectinesterase inhibitors, narbonin, and pectin methylesterase might trigger mechanical responses.

Macroevolution of somatic and reproductive organs relies on expansion of protein families, interacting partners and related network components (Kersting et al., 2015). Different organs require an appropriate sensing mechanism to maintain balance between wall biosynthesis and remodeling (Baumberger et al., 2001; Ringli, 2005; Rui & Dinneny, 2020; Tenhaken, 2014). We pointed that structural ECM proteins, such as extensin, fasciclin arabinogalactan, and armadillo might influence organ formation and cell semantic properties. Further studies by Caño‐Delgado et al. (2003) and others demonstrated that loss‐of‐function and gain‐of‐function of polysaccharide biogenesis related matrix proteins plays a role in modulating wall properties. We observed that cellulose synthase, β‐1,4 glucan synthase, β‐amylase, chalcone synthase, β‐glucosidase, laccase, β‐1,3 glucanase, peroxidases, chitinase, and chalcone flavanone isomerase that regulate wall polysaccharide biogenesis are conserved across different plant lineages. This prompted us to address a key question related to molecular evolution of plant ECM proteins and to understand whether the observed transition and transversion substitutions are due to selection disfavoring transversions. We found that transversions are less pronounced than transitions in ECM protein families. Thus, our study provides support for the selective hypothesis. Presence of plethora of carbohydrate degrading enzymes in plant matrisome from varied organs and clades is not surprising, but the interaction analysis and evolutionary significance of such members remain elusive. This study documented that some CDE orthologs might have evolved from a common ancestor through speciation and retained an original functionality. However, a few CDE paralogs shared a common ancestral gene that duplicated within the genome and might evolve new functions.

As evidenced by a lower number of synonymous substitutions (dS) and lower rates of silent‐site divergence, molecular evolution of ECM protein families occurred at slower rates in gymnosperm and algae than in angiosperm. This pattern of molecular evolutionary rate is consistent according to the life forms when assembling the species, from gymnosperms to angiosperms herbs and shrubs and trees (Benton et al., 2022). In our study, we detected a higher proportion of sites with positive selection in pairwise estimate analysis of all ECM protein families, berberine, CAZy, extensin, fascilin AGP, LRR proteins, peroxidase, Phi starvation protein, proteinase inhibitor, purple acid phoshatase, and lectin in particular, suggesting an important role of selection in the evolution of ECM proteome. Earlier reports have suggested that in angiosperms, mutations are proportionally strongly deleterious, and mildly deleterious mutations are relatively conserved among angiosperm lineages (Conover & Wendel, 2022; Gossmann et al., 2010). In contrast to the findings, our analysis and report by de la Torre et al. (2017) showed high proportion of sites under mild purifying selection in ECM protein families of angiosperm lineages. We observed that both dS and dN showed variation in ECM protein families across lineages.

Here, we also developed putative matrix proteoforms organ and taxonomic family network and provided insights into the mechanisms of wall‐associated events. Our dataset identified possible novel mediators of CWI responses in different plant lineage, such as iron accumulator ferritin, nitrogen assimilators glutamate dehydrogenase, carboxylase, reductase, and glutamine synthetase. We also found wall remodeling and carbon reallocation proteins related to galactose, arabinose, glucose, and fructose recycling. The list of candidate matrix proteins considerably expands the spectrum of biological functions regulated by wall‐associated processes. Identification of several matrix proteins associated with cutin deposition, including cutinase, GDSL esterase/lipase, phosphoesterase, and HOTHEAD protein suggests an important and hitherto underestimated role of CWI signaling in wall restructuring. We propose that this CWI signaling components should be prioritized for follow‐up experiments, both to validate predicted signaling components and to evaluate specific biological functions. For instance, it will be interesting to determine which (if any) of the oxidant‐mediated adhesion matrix protein have direct protein–protein interactions with the wall permeability and signaling related proteins, such as fibronectin like domain containing protein, GRPs, calreticulin, and expansin and to test our organ and taxonomic family network predictions about specific aspects of CWI signaling. Our results add another layer to the complex and fine regulation of wall reorganization. Several regulators of wall mechanical stimulation that leads to increase in Ca^2+^ levels, such as sensor (e.g., calmodulin), buffer (e.g., calreticulin), or Ca^2+^‐stabilized proteins (e.g., thermolysin, EF‐hand proteins, and EF2) were identified. Other potential CWI remodelers are involved in pH dependent protein turnover, wall maintenance, or disassembly and display lineage‐specific distribution. We provide evidence that divergence of plant matrix proteins includes subfunctionalization and neofunctionalization and concerted evolution as a result of gene duplication, gene gain, and loss that results in matrix protein family expansion and divergence. This molecular phenomenon along with known similarities in wall architecture implies that the basic architecture of plant ECM is ancestral to the divergence of the Monocot and Eudicot.

As detailed above, our results, particularly support and extend previously reported results for matrix protein functionality, interaction, and evolution underpinning the high quality of our data. We also investigated wall‐associated processes by defining matrix phosphoproteome dynamics in chickpea. We could confidently detect phosphorylation sites and motifs on wall integrity related matrix proteoforms. We for the first time were also able to identify several additional proteins that to our knowledge have not yet been associated with wall‐associated processes in plant. Given the complexity of the system and the dimensions of our global phosphoproteome dataset, we applied exploratory computational tools to connect wall‐associated events with matrix functionality. Based on our findings, we were able to generate new hypotheses for CWI pathway component interactions and matrix functionality (Figure 8b). Our study also contributes to the unexplored field of molecular evolution of ECM protein families in Tracheophytes by investigating the differences in rates of molecular evolution using a phylogenetic framework. Taken together, our data provide one of the most comprehensive plant matrisome studies across different taxonomic clades that identify CWI components and represent a unique resource to investigate new areas in plant matrix biology.

## AUTHOR CONTRIBUTIONS

Subhra Chakraborty conceived the experiments; Subhra Chakraborty, Kanika Narula, Arunima Sinha, and Pooja Choudhary designed the experiments; Kanika Narula, Arunima Sinha, Pooja Choudhary, Eman Elagamey, Archana Sharma, and Atreyee Sengupta performed the experiments; Kanika Narula, Arunima Sinha, Pooja Choudhary, Sudip Ghosh, Eman Elagamey, Niranjan Chakraborty, and Subhra Chakraborty analyzed the data; Subhra Chakraborty and Kanika Narula wrote the paper. All authors read and approved the final manuscript.

## CONFLICT OF INTEREST STATEMENT

The authors declare no conflict of interest.

## PEER REVIEW

The peer review history for this article is available in the Supporting Information for this article.

## Supporting information


**Table S1.** List of chickpea ECM protein identified by LC–MS/MS analysis. aSample name abbreviated as IGU, in‐gel unenriched and SCX, strong cation exchange followed by concentration of salt used for fractionation. The numbers were designated as CaWEP, where Ca indicates the organism ( *Cicer arietinum* ), W denotes chickpea variety WR‐315, E represent ECM fraction and P denotes ECM protein. b denotes Protein identification number as in Uniprot. c represent Number of peptides. mSubP;http://bioinfo.usu.edu/Plant-mSubP/. WoLFPSORT; https://wolfpsort.hgc.jp/

**Table S2.** The list of phosphorylated proteins, peptides and sites detected in ECM fraction of chickpea var. WR‐315. a Sample name abbreviated as IMAC, immobilized metal affinity chromatography and IS, in‐solution digested proteins. b denotes Protein identification number as in Genbank. c signifies Number of peptides. mSubP;http://bioinfo.usu.edu/Plant-mSubP/. WoLFPSORT; https://wolfpsort.hgc.jp/

**Table S3.** Compilation of available ECM proteome by literature search. References are enlisted in Supporting Information References.

**Table S4.** List of ECM proteins reported in available ECM proteomes as mentioned in Table S3.

**Table S5.** Pairwise estimate of ds, dn and its ratios in (a) berberine, (b) direngent, (c), CaZY, (d) expansin, (e) extensin, (f) fascilin arabinogalactan, (g) germin, (h) glycine rich protein, (i) lectin, (j) LRR, (k) peroxidase, (l) Phi starvation protein, (m) phosphatase, (n) protease, (o) proteinase inhibitors, (p) purple acid phosphatase. Sd: The number of observed synonymous substitutions; Sn: The number of observed non‐synonymous substitutions; S: The number of potential synonymous substitutions (the average for the two compared sequences); N: The number of potential non‐synonymous substitutions (the average for the two compared sequences); ps: The proportion of observed synonymous substitutions: Sd/S; pn: The proportion of observed non‐synonymous substitutions: Sn/N; ds: The Jukes‐Cantor correction for multiple hits of ps; dn: The Jukes‐Cantor correction for multiple hits of pn; ds/dn: The ratio of synonymous to non‐synonymous substitutions

**Table S6.** Branch estimate of ds, dn and its ratios in (a) berberine, (b) direngent, (c) CaZY, (d) expansin, (e) extensin, (f) fascilin arabinogalactan, (g) germin, (h) glycine rich protein, (i) lectin, (j) LRR, (k) peroxidase, (l) Phi starvation protein, (m) phosphatase, (n) protease, (o) proteinase inhibitors, (p) purple acid phosphatase

**Table S7.** Domain analysis of chickpea ( *Cicer arietinum* c.v. WR‐315) ECM proteome with unknown function. aSample origin based on technique used. The first letters (Ca) signify the source plant, Cicerarietinum, followed by WEP denotes WR 315 Extracellular Matrix Proteome. a denotes Sample origin based on technique used. The first letters (Ca) signify the source plant, *Cicer arietinum* , followed by WEP denotes WR 315 Extracellular Matrix Proteome. b shows gene identification number as in Uniprot. c denotes InterPro domain accession number. d depicts For each of the proteins identified as “unknown functions” in Table S1 the InterPro site was queried for domains in SMART, Panther, and Pfam databases to identify functional domains of each protein. N/F, not found; N/A, not applicable.

**Table S8.** Domain analysis of chickpea ( *Cicer arietinum* c.v. WR‐315) ECM phosphoproteins with unknown function. a denotes Sample origin based on technique used. The first letters (Ca) signify the source plant, *Cicer arietinum* , followed by WEP denotes WR 315 Extracellular Matrix phosphoproteome. b shows gene identification number as in Uniprot. c denotes InterPro domain accession number. d depicts For each of the phosphoproteins identified as “unknown functions” in Table S2 the InterPro site was queried for domains in SMART, Panther, and Pfam databases to identify functional domains of each protein. N/F, not found; N/A, not applicable.

**Table S9.** Fragment spectra of the proteins identified with a single peptide.

**Table S10.** Fragment spectra of the phosphoproteins identified with a single peptide.

**Figure S1.** Purity assessment of isolated ECM protein from chickpea seedlings. (a). Transmission electron micrographs of purified ECM. (b) Glucose 6 phosphate dehydrogenase activity Bars indicate activity in crude and ECM protein extract. (c) Catalase assay in ECM protein and crude fractions (d) Vanadate sensitive H^+^ ATPase activity in ECM protein, crude extract and plasma membrane protein.
**Figure S2.** Detailed workflow of (a) Comparative proteomics and phosphoproteomics study. ECM proteins were extracted from chickpea seedlings. Number of seedlings is indicated by n. Non‐redundant set of proteins was catalogued according to gene ontology using Blast2GO program for (b) Biological process and (c) molecular function. Pie chart showing functional categorization (d), ECM protein and (e) ECM phosphoprotein.
**Figure S3.** Expansion of plant ECM protein families. (a) CW‐known unknown proteins from phanerogamia and crypogamia B proteins related to lipid metabolism. Each row represents a taxonomic species and each column represents a organ/combination of organs showing expansion. Names of organs are given in red, whereas categories of ECM protein families are in different colored corrplot. The colour intensity and size of the circles are proportional to the number of protein falling under the given protein family. (b) Proteins related to lipid metabolism. Abbreviation: L, leaf; S, stem; R, root; CS, cell suspension; H, hypocotyl; F, fruit; I, inflorescence; SD, seed; SDN, seedling; L‐S‐R(V), vegetative.
**Figure S4.** Phylogenetic tree analysis of orthologous and paralogous ECM cluster proteins.
**Figure S5.** dN/dS (ka/Ks) tree of (a) Berberine; (b) CaZY; (c) Class 1 fascilin; (d) Class 2 fascilin; (e), Class 3 fascilin; (f), Glycine rich protein.
**Figure S6.** dN/dS (Ka/Ks) tree of (a), Direngent; (b) Germin; (c) Expansin; (d) Phi starvation protein; (e) Extensin; (f) Phosphatase.
**Figure S7.** dN/dS (ka/Ks) tree of (a) Lectin; (b), Proteinase inhibitor (c) LRR protein; (d) Purple acid phosphatase; (e) Fascilin.
**Figure S8.** dN/dS (ka/Ks) tree of Peroxidase
**Figure S9.** dN/dS (ka/Ks) tree of Protease.
**Figure S10.** Analysis of synonymous, non‐synonymous and indels in ECM proteins (a) Berberine. (b) Direngent protein. (c) CaZY. (d) Expansin. (e) Extensin. (f) Fascilin. (g) Germin. (h) Glycine rich protein. (i) Lectin. (j) LRR protein. (k) Peroxidase. (l) Phi starvation protein. (m) Phosphatase. (n) Protease. (o) Proteinase inhibitor. (p) Purple acid phosphatase.
**Figure S11.** Mismatch and transition‐transversion alignment of (a) Berberine, (b) Direngent protein and (c) CaZY.
**Figure S12.** Mismatch and transition‐transversion alignment of (a) Expansin, (b) Extensin and (c) Fascilin arabinogalactan protein.
**Figure S13.** Mismatch and transition‐transversion alignment of (a) Germin, (b) Glycine rich, (c) Lectin, and (d) Phi starvation protein.
**Figure S14.** Mismatch and transition‐transversion alignment of peroxidase.
**Figure S15.** Mismatch and transition‐transversion alignment of (a) phosphatase, (b) protease.
**Figure S16.** Mismatch and transition‐transversion alignment of (a) proteinase inhibitors, (b) purple acid phosphatase, (c) LRR.
**Figure S17.** Evolution and interaction of wall enzyme machinery based on plant matrisome. Evolutionary relationship of identified (a) wall enzyme machinery. (b) Network analysis depicting correlation among identified wall enzymes. Each node represents a given ECM protein associated based on top BLAST hit against SwissProt and an edge denotes a probability of two given proteins (nodes) potentially interacting based on the cytoprophet algorithm. Nodes and edges represent ECM proteins and correlation between proteins, respectively. Abbreviation: CDE, carbohydrade degrading enzyme.
**Figure S18.** Evolution and interaction of wall organization component based on plant matrisome. Evolutionary relationship of identified (a) Peroxidase, (b) Purple acid phosphatase, (c) Berberine, (d) Germin, (e) Network analysis depicting correlation among identified wall enzymes. Nodes and edges represent ECM proteins and correlation between proteins, respectively.
**Figure S19.** Evolutionary relationship of identified (a) protease, (b) protease inhibitor, (c) Network analysis depicting correlation among identified wall protein homeostasis machinery. Nodes and edges represent ECM proteins and correlation between proteins, respectively.
**Figure S20.** Evolutionary relationship of identified (a) Phi, (b) LRR, (c) Direngent protein, (d) Network analysis depicting correlation among identified miscellaneous protein. Nodes and edges represent ECM proteins and correlation between proteins, respectively.
**Figure S21.** 2‐DE analysis and spot detection. (a) ECM proteins and (b) ECM phosphoproteins were zoomed onto 24 cm IPG strip (pH 4–7) and second dimension was performed on 12.5% (w/v) SDS‐PAGE.

**Data S1.** Peer Review.

**Data S2** Supporting Information

## Data Availability

The data described in the manuscript are enlisted in Tables S1 and S2.

## References

[pld3572-bib-0001] Agrawal, L. , Narula, K. , Basu, S. , Shekhar, S. , Ghosh, S. , Datta, A. , Chakraborty, N. , & Chakraborty, S. (2013). Comparative proteomics reveals a role for seed storage protein, AmA1 in cellular growth, development and nutrient accumulation. Journal of Proteome Research, 12, 4904–4930. 10.1021/pr4007987 24024778

[pld3572-bib-0002] Baumberger, N. , Ringli, C. , & Keller, B. (2001). The chimeric leucine‐rich repeat/extensin cell wall protein LRX1 is required for root hair morphogenesis in *Arabidopsis thaliana* . Genes & Development, 15, 1128–1139. 10.1101/gad.200201 11331608 PMC312681

[pld3572-bib-0003] Belfield, E. J. , Gan, X. , Mithani, A. , Brown, C. , Jiang, C. , Franklin, K. , Alvey, E. , Wibowo, A. , Jung, M. , Bailey, K. , Kalwani, S. , Ragoussis, J. , Mott, R. , & Harberd, N. P. (2012). Genome‐wide analysis of mutations in mutant lineages selected following fast‐neutron irradiation mutagenesis of Arabidopsis thaliana. Genome Research, 22, 1306–1315. 10.1101/gr.131474.111 22499668 PMC3396371

[pld3572-bib-0004] Benton, M. J. , Wilf, P. , & Sauquet, H. (2022). The angiosperm terrestrial revolution and the origins of modern biodiversity. New Phytologist, 233, 2017–2035. 10.1111/nph.17822 34699613

[pld3572-bib-0005] Bermejo, C. , Rodríguez, E. , García, R. , Rodríguez‐Peña, J. M. , Rodríguez de la Concepción, M. L. , Rivas, C. , Arias, P. , Nombela, C. , Posas, F. , & Arroyo, J. (2008). The sequential activation of the yeast HOG and SLT2 pathways is required for cell survival to cell wall stress. Molecular Biology of the Cell, 19, 1113–1124. 10.1091/mbc.e07-08-0742 18184748 PMC2262984

[pld3572-bib-0006] Bhushan, D. , Pandey, A. , Chattopadhyay, A. , Choudhary, M. K. , Chakraborty, S. , Datta, A. , & Chakraborty, N. (2006). Extracellular matrix proteome of chickpea (*Cicer arietinum* L.) illustrates pathway abundance, novel protein functions and evolutionary perspect. Journal of Proteome Research, 5, 1711–1720. 10.1021/pr060116f 16823979

[pld3572-bib-0007] Bradley, E. L. , Ökmen, B. , Doehlemann, G. , Henrissat, B. , Bradshaw, R. E. , & Mesarich, C. H. (2022). Secreted glycoside hydrolase proteins as effectors and invasion patterns of plant‐associated fungi and oomycetes. Frontiers in Plant Science, 13, 853106. 10.3389/fpls.2022.853106 35360318 PMC8960721

[pld3572-bib-0008] Brutus, A. , Sicilia, F. , Macone, A. , Cervone, F. , & de Lorenzo, G. (2010). A domain swap approach reveals a role of the plant wall‐associated kinase 1 (WAK1) as a receptor of oligogalacturonides. Proceedings of the National Academy of Sciences, USA, 107, 9452–9457. 10.1073/pnas.1000675107 PMC2889104 20439716

[pld3572-bib-0009] Cabrera, J. C. , Boland, A. , Cambier, P. , Frettinger, P. , & van Cutsem, P. (2010). Chitosan oligosaccharides modulate the supramolecular conformation and the biological activity of oligogalacturonides in Arabidopsis. Glycobiology, 20, 775–786. 10.1093/glycob/cwq034 20200051

[pld3572-bib-0010] Cannon, M. C. , Terneus, K. , Hall, Q. , Tan, L. , Wang, Y. , Wegenhart, B. L. , Chen, L. , Lamport, D. T. , Chen, Y. , & Kieliszewski, M. J. (2008). Self‐assembly of the plant cell wall requires an extensin scaffold. Proceedings of the National Academy of Sciences, USA, 12, 2226–2231. 10.1073/pnas.0711980105 PMC2538902 18256186

[pld3572-bib-0011] Caño‐Delgado, A. , Penfield, S. , Smith, C. , Catley, M. , & Bevan, M. (2003). Reduced cellulose synthesis invokes lignification and defense responses in Arabidopsis thaliana. The Plant Journal, 34, 351–362. 10.1046/j.1365-313X.2003.01729.x 12713541

[pld3572-bib-0012] Canut, H. , Albenne, C. , & Jamet, E. (2016). Post‐translational modifications of plant cell wall proteins and peptides: A survey from a proteomics point of view. Biochimica et Biophysica Acta, 1864, 983–990. 10.1016/j.bbapap.2016.02.022 26945515

[pld3572-bib-0013] Capella‐Gutiérrez, S. , Silla‐Martínez, J. M. , & Gabaldón, T. (2009). trimAl: A tool for automated alignment trimming in large‐scale phylogenetic analyses. Bioinformatics, 25, 1972–1973. 10.1093/bioinformatics/btp348 19505945 PMC2712344

[pld3572-bib-0014] Cassab, G. I. (1998). Plant cell wall proteins. Annual Review of Plant Physiology and Plant Molecular Biology, 49, 281–309. 10.1146/annurev.arplant.49.1.281 15012236

[pld3572-bib-0015] Chivasa, S. , Simon, W. J. , Yu, X. L. , Yalpani, N. , & Slabas, A. R. (2005). Pathogen elicitor‐induced changes in the maize extracellular matrix proteome. Proteomics, 5, 4894–4904. 10.1002/pmic.200500047 16281185

[pld3572-bib-0016] Codjoe, J. M. , Miller, K. , & Haswell, E. S. (2022). Plant cell mechanobiology: Greater than the sum of its parts. Plant Cell, 34, 129–145. 10.1093/plcell/koab230 34524447 PMC8773992

[pld3572-bib-0017] Coleman, H. D. , Yan, J. , & Mansfield, S. D. (2009). Sucrose synthase affects carbon partitioning to increase cellulose production and altered cell wall ultrastructure. Proceedings of the National Academy of Sciences, USA, 106, 13118–13123. 10.1073/pnas.0900188106 PMC2722352 19625620

[pld3572-bib-0018] Conover, J. L. , & Wendel, J. F. (2022). Deleterious mutations accumulate faster in allopolyploid than diploid cotton (Gossypium) and unequally between subgenomes. Molecular Biology and Evolution, 39, msac024. 10.1093/molbev/msac024 35099532 PMC8841602

[pld3572-bib-0019] de la Torre, A. R. , Li, Z. , van de Peer, Y. , & Ingvarsson, P. K. (2017). Contrasting rates of molecular evolution and patterns of selection among gymnosperms and flowering plants. Molecular Biology and Evolution, 34, 1363–1377. 10.1093/molbev/msx069 28333233 PMC5435085

[pld3572-bib-0020] Dehors, J. , Mareck, A. , Kiefer‐Meyer, M. C. , Menu‐Bouaouiche, L. , Lehner, A. , & Mollet, J. C. (2019). Evolution of cell wall polymers in tip‐growing land plant gametophytes: Composition, distribution, functional aspects and their remodeling. Frontiers in Plant Science, 10, 441. 10.3389/fpls.2019.00441 31057570 PMC6482432

[pld3572-bib-0021] Desprez, T. , Vernhettes, S. , Fagard, M. , Refregier, G. , Desnos, T. , Aletti, E. , et al. (2002). Resistance against herbicide isoxaben and cellulose deficiency caused by distinct mutations in same cellulose synthase isoform CESA6. Plant Physiology, 128, 482–490. 10.1104/pp.010822 11842152 PMC148911

[pld3572-bib-0022] Diet, A. , Link, B. , Seifert, G. J. , Schellenberg, B. , Wagner, U. , Pauly, M. , Reiter, W. D. , & Ringli, C. (2006). The Arabidopsis root hair cell wall formation mutant lrx1 is suppressed by mutations in the RHM1 gene encoding a UDP‐L‐rhamnose synthase. Plant Cell, 18, 1630–1641. 10.1105/tpc.105.038653 16766693 PMC1488927

[pld3572-bib-0023] Eddy, S. R. (1995). Multiple alignment using hidden Markov models. Proceedings International Conference on Intelligent Systems for Molecular Biology, 3, 114–120. 7584426

[pld3572-bib-0024] Elagamey, E. , Narula, K. , Chakraborty, N. , & Chakraborty, S. (2020). Extracellular matrix proteome: Isolation of ECM proteins for proteomics studies. In K. Gupta (Ed.), Nitrogen metabolism in plants. Methods in molecular biology (Vol. 2057). Humana. 10.1007/978-1-4939-9790-9_14 31595478

[pld3572-bib-0025] Elagamey, E. , Narula, K. , Sinha, A. , Ghosh, S. , Abdellatef, M. A. E. , Chakraborty, N. , & Chakraborty, S. (2017). Quantitative extracellular matrix proteomics suggests cell wall reprogramming in host‐specific immunity during vascular wilt caused by *Fusarium oxysporum* in chickpea. Proteomics, 17, 1600374. 10.1002/pmic.201600374 29144021

[pld3572-bib-0026] Fangel, J. U. , Ulvskov, P. , Knox, J. P. , Mikkelsen, M. D. , Harholt, J. , Popper, Z. A. , & Willats, W. G. T. (2012). Cell wall evolution and diversity. Frontiers in Plant Science, 3, 152. 10.3389/fpls.2012.00152 22783271 PMC3390603

[pld3572-bib-0027] Friso, G. , & van Wijk, K. J. (2015). Posttranslational protein modifications in plant metabolism. Plant Physiology, 169, 1469–1487. 10.1104/pp.15.01378 26338952 PMC4634103

[pld3572-bib-0028] Gaut, B. , Yang, L. , Takuno, S. , & Eguiarte, L. E. (2011). The patterns and causes of variation in plant nucleotide substitution rates. Annual Review of Ecology, Evolution, and Systematics, 42, 245–266. 10.1146/annurev-ecolsys-102710-145119

[pld3572-bib-0029] Gilbert, H. J. (2010). The biochemistry and structural biology of plant cell wall deconstruction. Plant Physiology, 153, 444–455. 10.1104/pp.110.156646 20406913 PMC2879781

[pld3572-bib-0030] Gossmann, T. I. , Song, B. H. , Windsor, A. J. , Mitchell‐Olds, T. , Dixon, C. J. , Kapralov, M. V. , Filatov, D. A. , & Eyre‐Walker, A. (2010). Genome wide analyses reveal little evidence for adaptive evolution in many plant species. Molecular Biology and Evolution, 27, 1822–1832. 10.1093/molbev/msq079 20299543 PMC2915642

[pld3572-bib-0031] Grosberg, R. K. , & Strathmann, R. R. (2007). The evolution of multicellularity: A minor major transition? Annual Review of Ecology, Evolution, and Systematics, 38, 621–654. 10.1146/annurev.ecolsys.36.102403.114735

[pld3572-bib-0032] Guan, Y. , & Nothnagel, E. A. (2004). Binding of arabinogalactan proteins by Yariv phenylglycoside triggers wound‐like responses in Arabidopsis cell cultures. Plant Physiology, 135, 1346–1366. 10.1104/pp.104.039370 15235117 PMC519053

[pld3572-bib-0033] Guerra‐Guimarães, L. , Pinheiro, C. , Chaves, I. , Barros, D. R. , & Ricardo, C. P. (2016). Protein dynamics in the plant extracellular space. Proteomes, 4, 22. 10.3390/proteomes4030022 28248232 PMC5217353

[pld3572-bib-0034] Hamann, T. , & Denness, L. (2011). Cell wall integrity maintenance in plants: Lessons to be learned from yeast? Plant Signaling & Behavior, 6, 1–5. 10.4161/psb.6.11.17782 22067998 PMC3329341

[pld3572-bib-0035] Hoffmann, N. , King, S. , Samuels, A. L. , & McFarlane, H. E. (2021). Subcellular coordination of plant cell wall synthesis. Developmental Cell, 56, 933–948. 10.1016/j.devcel.2021.03.004 33761322

[pld3572-bib-0036] Humphrey, T. V. , & Bonetta, D. T. (2007). Goring DR. Sentinels at the wall: Cell wall receptors and sensors. New Phytologist, 176, 7–21. 10.1111/j.1469-8137.2007.02192.x 17803638

[pld3572-bib-0037] Jamet, E. , Canut, H. , Boudart, G. , & Pont‐Lezica, R. F. (2006). Cell wall proteins: A new insight through proteomics. Trends in Plant Science, 11, 33–39. 10.1016/j.tplants.2005.11.006 16356755

[pld3572-bib-0038] Jolie, R. P. , Duvetter, T. , van Loey, A. M. , & Hendrickx, M. E. (2010). Pectin methylesterase and its proteinaceous inhibitor: A review. Carbohydrate Research, 345, 2583–2595. 10.1016/j.carres.2010.10.002 21047623

[pld3572-bib-0039] Kang, B. H. , Busse, J. S. , & Bednarek, S. Y. (2003). Members of the Arabidopsis dynamin‐like gene family, ADL1, are essential for plant cytokinesis and polarized cell growth. The Plant Cell, 15, 899–913. 10.1105/tpc.009670 12671086 PMC524700

[pld3572-bib-0040] Kenrick, P. , & Crane, P. R. (1997). The origin and early evolution of plants on land. Nature, 389, 33–39. 10.1038/37918

[pld3572-bib-0041] Kersting, A. R. , Mizrachi, E. , Bornberg‐Bauer, E. , & Myburg, A. A. (2015). Protein domain evolution is associated with reproductive diversification and adaptive radiation in the genus Eucalyptus. New Phytologist, 206, 1328–1336. 10.1111/nph.13211 25494981

[pld3572-bib-0042] Keyes, G. , Sorrells, M. E. , & Setter, T. L. (1990). Gibberellic acid regulates cell wall extensibility in wheat (*Triticum aestivum* L.). Plant Physiology, 92, 242–245. 10.1104/pp.92.1.242 16667254 PMC1062276

[pld3572-bib-0043] Kimura, M. , & Ohta, T. (1971). Protein polymorphism as a phase of molecular evolution. Nature, 229, 467–469. 10.1038/229467a0 4925204

[pld3572-bib-0044] Liu, Z. , Persson, S. , & Sánchez‐Rodríguez, C. (2015). At the border: The plasma membrane‐cell wall continuum. Journal of Experimental Botany, 66, 1553–1563. 10.1093/jxb/erv019 25697794

[pld3572-bib-0045] Lyons, D. M. , & Lauring, A. S. (2017). Evidence for the selective basis of transition‐to‐transversion substitution bias in two RNA viruses. Molecular Biology and Evolution, 34, 3205–3215. 10.1093/molbev/msx251 29029187 PMC5850290

[pld3572-bib-0046] Manfield, I. W. , Orfila, C. , McCartney, L. , Harholt, J. , Bernal, A. J. , Scheller, H. V. , Gilmartin, P. M. , Mikkelsen, J. D. , Paul Knox, J. , & Willats, W. G. T. (2004). Novel cell wall architecture of isoxaben‐habituated Arabidopsis suspension‐cultured cells: Global transcript profiling and cellular analysis. The Plant Journal, 40, 260–275. 10.1111/j.1365-313X.2004.02208.x 15447652

[pld3572-bib-0047] Manimaran, P. , Mangrauthia, S. K. , Sundaram, R. M. , & Balachandran, S. M. (2015). Constitutive expression and silencing of a novel seed specific calcium dependent protein kinase gene in rice reveals its role in grain filling. Journal of Plant Physiology, 174, 41–48. 10.1016/j.jplph.2014.09.005 25462965

[pld3572-bib-0048] Marín, M. J. , Flández, M. , Bermejo, C. , Arroyo, J. , Martín, H. , & Molina, M. (2009). Different modulation of the outputs of yeast MAPK mediated pathways by distinct stimuli and isoforms of the dual‐specificity phosphatase Msg5. Molecular Genetics and Genomics, 281, 345–359. 10.1007/s00438-008-0415-5 19123063

[pld3572-bib-0049] Martín, H. , Rodríguez‐Pachón, J. M. , Ruiz, C. , Nombela, C. , & Molina, M. (2000). Regulatory mechanisms for modulation of signaling through the cell integrity Slt2‐mediated pathway in Saccharomyces cerevisiae. Journal of Biological Chemistry, 275, 1511–1519. 10.1074/jbc.275.2.1511 10625705

[pld3572-bib-0050] Miyazawa, S. (2011). Selective constraints on amino acids estimated by a mechanistic codon substitution model with multiple nucleotide changes. PLoS ONE, 6(3), e17244. 10.1371/journal.pone.0017244 21445250 PMC3060808

[pld3572-bib-0051] Morcos, F. , Lamanna, C. , Sikora, M. , & Izaguirre, J. (2008). Cytoprophet: A Cytoscape plug‐in for protein and domain interaction networks inference. Bioinformatics, 24, 2265–2266. 10.1093/bioinformatics/btn380 18653520

[pld3572-bib-0052] Moscatiello, R. , Mariani, P. , Sanders, D. , & Maathuis, F. J. (2006). Transcriptional analysis of calcium‐dependent and calcium‐independent signalling pathways induced by oligogalacturonides. Journal of Experimental Botany, 57, 2847–2865. 10.1093/jxb/erl043 16868046

[pld3572-bib-0053] Ndimba, B. K. , Chivasa, S. , Hamilton, J. M. , Simon, W. J. , & Slabas, A. R. (2003). Proteomic analysis of changes in the extracellular matrix of Arabidopsis cell suspension cultures induced by fungal elicitors. Proteomics, 3, 1047–1059. 10.1002/pmic.200300413 12833529

[pld3572-bib-0054] Okumura, M. , & Kinoshita, T. (2016). Measurement of ATP hydrolytic activity of plasma membrane H+‐ATPase from arabidopsis thaliana leaves. Bio‐Protocol, 6, e2044. 10.21769/BioProtoc.2044

[pld3572-bib-0055] Podder, A. , Raju, A. , & Schork, N. J. (2021). Cross‐species and human inter‐tissue network analysis of genes implicated in longevity and aging reveal strong support for nutrient sensing. Frontiers in Genetics, 12, 719713. 10.3389/fgene.2021.719713 34512728 PMC8430347

[pld3572-bib-0056] Polko, J. K. , van Zanten, M. , van Rooij, J. A. , Marée, A. F. , Voesenek, L. A. , Peeters, A. J. , et al. (2012). Ethylene‐induced differential petiole growth in Arabidopsis thaliana involves local microtubule reorientation and cell expansion. New Phytologist, 193, 339–348. 10.1111/j.1469-8137.2011.03920.x 21973123

[pld3572-bib-0057] Ringli, C. (2005). The role of extracellular LRR‐extensin (LRX) proteins in cell wall formation. Plant Biosystems, 139, 32–35. 10.1080/11263500500059892

[pld3572-bib-0058] Rui, Y. , & Dinneny, J. R. (2020). A wall with integrity: Surveillance and maintenance of the plant cell wall under stress. The New Phytologist, 225, 1428–1439. 10.1111/nph.16166 31486535

[pld3572-bib-0059] Sarkar, P. , Bosneaga, E. , & Auer, M. (2009). Plant cell walls throughout evolution: Towards a molecular understanding of their design principles. Journal of Experimental Botany, 60, 3615–3635. 10.1093/jxb/erp245 19687127

[pld3572-bib-0060] Schröder, R. , Atkinson, R. G. , & Redgwell, R. J. (2009). Re‐interpreting the role of endo‐beta‐mannanases as mannan endotransglycosylase/hydrolases in the plant cell wall. Annals of Botany, 104, 197–204. 10.1093/aob/mcp120 19454593 PMC2710900

[pld3572-bib-0061] Seifert, G. J. , & Blaukopf, C. (2010). Irritable walls: The plant extracellular matrix and signaling. Plant Physiology, 153, 467–478. 10.1104/pp.110.153940 20154095 PMC2879813

[pld3572-bib-0062] Shimazaki, K. , & Kondo, N. (1987). Plasma membrane H+‐ATPase in guard‐cell protoplasts from Vicia faba L. Plant and Cell Physiology, 28, 893–900. 10.1093/oxfordjournals.pcp.a077371

[pld3572-bib-0063] Shin, Y. , Chane, A. , Jung, M. , & Lee, Y. (2021). Recent advances in understanding the roles of pectin as an active participant in plant signaling networks. Plants (Basel), 10, 1712. 10.3390/plants10081712 34451757 PMC8399534

[pld3572-bib-0064] Simcox, P. D. , Reid, E. E. , Canvin, D. T. , & Dennis, D. T. (1977). Enzymes of the glycolytic and pentose phosphate pathways in proplastids from the developing endosperm of Ricinus communis L. Plant Physiology, 59, 1128–1132. 10.1104/pp.59.6.1128 16660007 PMC542520

[pld3572-bib-0065] Sørensen, I. , Domozych, D. , & Willats, W. G. (2010). How have plant cell walls evolved? Plant Physiology, 153, 366–372. 10.1104/pp.110.154427 20431088 PMC2879805

[pld3572-bib-0066] Stolzer, M. , Lai, H. , Xu, M. , Sathaye, D. , Vernot, B. , & Durand, D. (2012). Inferring duplications, losses, transfers and incomplete lineage sorting with nonbinary species trees. Bioinformatics, 28, i409–i415. 10.1093/bioinformatics/bts386 22962460 PMC3436813

[pld3572-bib-0067] Sun, T. P. (2010). Gibberellin‐GID1‐DELLA: A pivotal regulatory module for plant growth and development. Plant Physiology, 154, 567–570. 10.1104/pp.110.161554 20921186 PMC2949019

[pld3572-bib-0068] Tamura, K. , Dudley, J. , Nei, M. , & Kumar, S. (2007). MEGA4: Molecular evolutionary genetics analysis (MEGA) software version 4.0. Molecular Biology and Evolution, 24, 1596–1599. 10.1093/molbev/msm092 17488738

[pld3572-bib-0069] Tang, W. , Deng, Z. , Oses‐Prieto, J. A. , Suzuki, N. , Zhu, S. , Zhang, X. , Burlingame, A. L. , & Wang, Z. Y. (2008). Proteomics studies of brassinosteroid signal transduction using prefractionation and two‐dimensional DIGE. Molecular and Cellular Proteomics, 7, 728–738. 10.1074/mcp.M700358-MCP200 18182375 PMC2401332

[pld3572-bib-0070] Tenhaken, R. (2014). Cell wall remodeling under abiotic stress. Frontiers in Plant Science, 5, 771. 25709610 10.3389/fpls.2014.00771 PMC4285730

[pld3572-bib-0071] Tian, H. , Lv, B. , Ding, T. , Bai, M. , & Ding, Z. (2018). Auxin‐BR interaction regulates plant growth and development. Frontiers in Plant Science, 8, 2256. 10.3389/fpls.2017.02256 29403511 PMC5778104

[pld3572-bib-0072] Tsang, D. L. , Edmond, C. , Harrington, J. L. , & Nuhse, T. S. (2011). Cell wall integrity controls root elongation via a general 1‐aminocyclopropane‐1‐carboxylic acid‐dependent, ethylene‐independent pathway. Plant Physiology, 156, 596–604. 10.1104/pp.111.175372 21508182 PMC3177261

[pld3572-bib-0073] Vaahtera, L. , Schulz, J. , & Hamann, T. (2019). Cell wall integrity maintenance during plant development and interaction with the environment. Nature Plants, 5, 924–932. 10.1038/s41477-019-0502-0 31506641

[pld3572-bib-0074] Verbančič, J. , Lunn, J. E. , Stitt, M. , & Persson, S. (2018). Carbon supply and the regulation of cell wall synthesis. Molecular Plant, 11, 75–94. 10.1016/j.molp.2017.10.004 29054565

[pld3572-bib-0075] Wang, D. , Zhang, Y. , Zhang, Z. , Zhu, J. , & Yu, J. (2010). KaKs_Calculator 2.0: A toolkit incorporating gamma—Series methods and sliding window strategies. Genomics, Proteomics & Bioinformatics, 8, 77–80. 10.1016/S1672-0229(10)60008-3 PMC5054116 20451164

[pld3572-bib-0076] Wilson, D. , Pethica, R. , Zhou, Y. , Talbot, C. , Vogel, C. , Madera, M. , Chothia, C. , & Gough, J. (2009). SUPERFAMILY—Sophisticated comparative genomics, data mining, visualization and phylogeny. Nucleic Acids Research, 37, D380–D386. 10.1093/nar/gkn762 19036790 PMC2686452

[pld3572-bib-0077] Wisecaver, J. H. , Slot, J. C. , & Rokas, A. (2014). The evolution of fungal metabolic pathways. PLoS Genetics, 10, e1004816. 10.1371/journal.pgen.1004816 25474404 PMC4256263

[pld3572-bib-0078] Yang, Z. , & Bielawski, J. P. (2000). Statistical methods for detecting molecular adaptation. Trends in Ecology & Evolution, 15, 496–503. 10.1016/S0169-5347(00)01994-7 11114436 PMC7134603

[pld3572-bib-0079] Zhang, B. , Gao, Y. , Zhang, L. , & Zhou, Y. (2021). The plant cell wall: Biosynthesis, construction, and functions. Journal of Integrative Plant Biology, 63, 251–272. 10.1111/jipb.13055 33325153

