# Supplementary material for: Combining extracellular matrix proteome and phosphoproteome of chickpea and meta‐analysis reveal novel proteoforms and evolutionary significance of clade‐specific wall‐associated events in plant
Source: Plant Direct. 2024 Mar 18;8(3):e572. doi: 10.1002/pld3.572 (PMC10945595; doi:10.1002/pld3.572)
Supplement: Supplementary file 7 — Table S7. Domain analysis of chickpea ( Cicer arietinum c.v. WR‐315) ECM proteome with unknown function. aSample origin based on technique used. The first letters (Ca) signify the source plant, Cicerarietinum, followed by WEP denotes WR 315 Extracellular Matrix Proteome. a denotes Sample origin based on technique used. The first letters (Ca) signify the source plant, Cicer arietinum , followed by WEP denotes WR 315 Extracellular Matrix Proteome. b shows gene identification number as in Uniprot. c denotes InterPro domain accession number. d depicts For each of the proteins identified as “unknown functions” in Table S1 the InterPro site was queried for domains in SMART, Panther, and Pfam databases to identify functional domains of each protein. N/F, not found; N/A, not applicable. [file PLD3-8-e572-s006.docx]

**Table S7.** Domain analysis of chickpea (*Cicer arietinum* c.v. WR-315) ECM proteome with unknown function

| **Sample origin** | **Protein Name** | **Accession No.** | **Interpro** | **Domain name** |
| --- | --- | --- | --- | --- |
| CaWEP-SCX 30mM-2, CaWEP-IGU-500, CaWEP-SCX 60mM-5 | Uncharacterized protein | XP_012569856.1 | IPR017853 | Glycoside hydrolase, superfamily |
|  |  |  | IPR001764 | Glycoside hydrolase, family 3, N-terminal |
|  |  |  | IPR002772 | Glycoside hydrolase family 3 C-terminal domain |
| CaWEP-IGU-578 | Uncharacterized protein | A0A1S2XIK8 | IPR036291 | NAD(P)-bd_dom_sf |
|  |  |  | IPR000683 | Oxidoreductase_N |
|  |  |  | PF01408 | GFO_IDH_MocA﻿, 1 hit |
| CaWEP-IGU-1175, CaWEP-IGU-1203 | Uncharacterized protein | XP_012569579.1 | NA |  |
|  |  |  |  |  |
| CaWEP-IGU-592 | Uncharacterized protein | A0A1S2XU24 | IPR036758 | At5g01610-like |
|  |  |  | IPR007493 | DUF538 |
| CaWEP-IGU-626 | Uncharacterized protein | A0A1S2YIE3 | IPR032675 | LRR_dom_sf |
|  |  |  | IPR013210 | LRR_N_plant-typ |
| CaWEP-SCX 60mM-17, CaWEP-IGU-25 | Uncharacterized protein | XP_027188384.1 | NF | NA |
|  |  |  |  |  |
| CaWEP-IGU-27 | Uncharacterized protein Putative | XP_004509072.1 | NF | NA |
| CaWEP-SCX 60mM-20, CaWEP-IGU-1, CaWEP-SCX 60mM-170 | Uncharacterized protein Putative | XP_027187506.1 | IPR017853 | Glycoside hydrolase, superfamily |
|  |  |  | IPR001764 | Glycoside hydrolase, family 3, N-terminal |
|  |  |  | IPR002772 | Glycoside hydrolase family 3 C-terminal domain |
|  |  |  |  |  |
| CaWEP-IGU-697, CaWEP-SEU-2, CaWEP-SEU-4, CaWEP-SCX 60mM-29, CaWEP-SEU-3, CaWEP-SCX 60mM-32 | Uncharacterized protein Putative | XP_004514101.1 | IPR013215 | Cobalamin-independent methionine synthase MetE, N-terminal |
|  |  |  | IPR002629 | Cobalamin-independent methionine synthase MetE, C-terminal/archaeal |
| CaWEP-SCX 60mM-27 | Uncharacterized protein | XP_004506800.1 | NF | NA |
| CaWEP-IGU-58 | Uncharacterized protein | NP_001266025.1 | IPR011992 | EF-hand domain pair |
|  |  |  | IPR002048 | EF-hand domain |
|  |  |  | IPR004360 | Glyoxalase/fosfomycin resistance/dioxygenase domain |
| CaWEP-SCX 60mM-42 | Uncharacterized protein Putative | XP_004504958.1 | NF | NA |
|  |  |  |  |  |
| CaWEP-IGU-85, CaWEP-SCX 60mM-53 | Uncharacterized protein | XP_027192838.1 | NF | NA |
|  |  |  |  |  |
| CaWEP-IGU-92 | Uncharacterized protein | C4J1B5 | NF | NA |
|  |  |  |  |  |
| CaWEP-SCX 60mM-67 | Uncharacterized protein Putative | XP_004486880.1 | IPR009057 | Homeodomain-like |
|  |  |  | IPR001005 | SANT/Myb domain |
|  |  |  |  |  |
| CaWEP-IGU-144 | Uncharacterized protein Putative | XP_027186730.1 | IPR009057 | Homeodomain-like |
|  |  |  | IPR001005 | SANT/Myb domain |
|  |  |  | IPR023633 | ATPase, F1 complex, gamma subunit domain |
| CaWEP-IGU-787 | Uncharacterized protein | XP_027191240.1 | IPR027417 | P-loop containing nucleoside triphosphate hydrolase |
|  |  |  | IPR000194 | ATPase, F1/V1/A1 complex, alpha/beta subunit, nucleotide-binding domain |
|  |  |  | IPR000793 | ATPase, F1/V1/A1 complex, alpha/beta subunit, C-terminal |
|  |  |  | IPR024034 | ATPase, F1 complex beta subunit/V1 complex, C-terminal |
|  |  |  | IPR020546 | ATPase, F1 complex, delta/epsilon subunit, N-terminal |
|  |  |  | IPR020547 | ATPase, F1 complex, delta/epsilon subunit, C-terminal domain |
| CaWEP-IGU-210 | Uncharacterized protein | XP_004507409.1 | NF | NA |
|  |  |  |  |  |
| CaWEP-IGU-219, CaWEP-SCX 60mM-126 | Uncharacterized protein | XP_004487696.1 | IPR012336 | Thioredoxin-like fold |
|  |  |  | IPR013766 | Thioredoxin domain |
|  |  |  |  |  |
| CaWEP-IGU-294, CaWEP-SCX 60mM-153, CaWEP-IGU-250, CaWEP-SCX 60mM-226, CaWEP-SCX 30mM-63, CaWEP-IGU-323 | Uncharacterized protein | XP_012571753.1 | IPR017853 | Glycoside hydrolase, superfamily |
|  |  |  | IPR001764 | Glycoside hydrolase, family 3, N-terminal |
|  |  |  | IPR002772 | Glycoside hydrolase family 3 C-terminal domain |
|  |  |  |  |  |
| CaWEP-IGU-483 | Uncharacterized protein | XP_004492691.1 | IPR011990 | Tetratricopeptide-like helical domain |
|  |  |  |  |  |
| CaWEP-IGU-500, CaWEP-SCX 60mM-5, CaWEP-SCX 30mM-2 | Uncharacterized protein | XP_012569856.1 | IPR017853 | Glycoside hydrolase, superfamily |
|  |  |  | IPR001764 | Glycoside hydrolase, family 3, N-terminal |
|  |  |  | IPR002772 | Glycoside hydrolase family 3 C-terminal domain |
|  |  |  |  |  |
| CaWEP-IGU-503 | Uncharacterized protein | YP_002149717.1 | IPR017443 | Ribulosebisphosphate carboxylase, large subunit, ferrodoxin-like N-terminal |
|  |  |  | IPR017444 | Ribulose bisphosphate carboxylase, large subunit, N-terminal |
|  |  |  | IPR000685 | Ribulose bisphosphate carboxylase, large subunit, C-terminal |
|  |  |  |  |  |
| CaWEP-IGU-507 | Uncharacterized protein | XP_027193036.1 | IPR008972 | Cupredoxin |
|  |  |  | IPR011707 | Multicopper oxidase, type 3 |
|  |  |  | IPR001117 | Multicopper oxidase, type 1 |
|  |  |  | IPR011706 | Multicopper oxidase, type 2 |
|  |  |  |  |  |
| CaWEP-IGU-512 | Uncharacterized protein | XP_004492663.1 | IPR002048 | EF-hand domain |
|  |  |  | IPR011992 | EF-hand domain pair |
|  |  |  |  |  |
| CaWEP-SCX 60mM-291 | Uncharacterized protein | XP_004492493.1 | NF | NA |
|  |  |  |  |  |
| CaWEP-SCX 30mM-89, CaWEP-SCX 60mM-133 | Uncharacterized protein | XP_004495434.1 | IPR014710 | RmlC-like jelly roll fold |
|  |  |  | IPR011051 | RmlC-like cupin domain |
|  |  |  | IPR008579 | Domain of unknown function DUF861, cupin-3 |
|  |  |  |  |  |
| CaWEP-SCX 30mM-90, CaWEP-SCX 60mM-309 | Uncharacterized protein | XP_004495638.1 | NF | NA |
|  |  |  |  |  |
| CaWEP-SCX 60mM-313 | Uncharacterized protein | XP_004496927.1 | NF | NA |
|  |  |  |  |  |
| CaWEP-IGU-974, CaWEP-SCX 60mM-340, CaWEP-SCX 30mM-101, CaWEP-IGU-974 | Uncharacterized protein, predicted | XP_004504566.1 | IPR012336 | Thioredoxin-like fold |
|  |  |  |  |  |
| CaWEP-SCX 60mM-343 | Uncharacterized protein | XP_004505146.1 | NF | NA |
|  |  |  |  |  |
| CaWEP-IGU-988, CaWEP-SCX 30mM-110, CaWEP-SCX 60mM-355 | Uncharacterized protein, predicted | XP_004509596.1 | IPR016140 | Bifunctional inhibitor/plant lipid transfer protein/seed storage helical domain |

^a^Sample origin based on technique used. The first letters (Ca) signify the source plant, *Cicer arietinum*, followed by WEP denotes WR 315 Extracellular Matrix Proteome.

^b^Gene identification number as in Uniprot

^c^InterPro domain accession number.

^d^For each of the proteins identified as “unknown functions” in Supplementary Table S1 the InterPro site was queried for domains in SMART, Panther, and Pfam databases to identify functional domains of each protein. N/F, not found; N/A, not applicable.
