# Supplementary material for: Combining extracellular matrix proteome and phosphoproteome of chickpea and meta‐analysis reveal novel proteoforms and evolutionary significance of clade‐specific wall‐associated events in plant
Source: Plant Direct. 2024 Mar 18;8(3):e572. doi: 10.1002/pld3.572 (PMC10945595; doi:10.1002/pld3.572)
Supplement: Supplementary file 8 — Table S8. Domain analysis of chickpea ( Cicer arietinum c.v. WR‐315) ECM phosphoproteins with unknown function. a denotes Sample origin based on technique used. The first letters (Ca) signify the source plant, Cicer arietinum , followed by WEP denotes WR 315 Extracellular Matrix phosphoproteome. b shows gene identification number as in Uniprot. c denotes InterPro domain accession number. d depicts For each of the phosphoproteins identified as “unknown functions” in Table S2 the InterPro site was queried for domains in SMART, Panther, and Pfam databases to identify functional domains of each protein. N/F, not found; N/A, not applicable. [file PLD3-8-e572-s005.docx]

**Table S8.**Domain analysis of chickpea (*Cicer arietinum* c.v. WR-315) ECM phosphoproteins with unknown function

| **Sample origin^a^** | **Protein Name** | **Accession No^b^** | **Interpro^c^** | **Domain name^d^** |
| --- | --- | --- | --- | --- |
| CaWEPP-IMAC-1DE1-477 | Uncharacterized protein | A0A022RAQ8 | IPR008972 | Cupredoxin |
|  |  |  | IPR011707 | Multicopper oxidase, type 3 |
|  |  |  | IPR001117 | Multicopper oxidase, type 1 |
|  |  |  | IPR011706 | Multicopper oxidase, type 2 |
| CaWEPP-IMAC-1DE1-217 | VITISV_028749 Putative uncharacterized protein | A5BNX1 | IPR001117. | Cu-oxidase. |
|  |  |  | IPR011706. | Cu-oxidase_2 |
|  |  |  | IPR011707. | Cu-oxidase_3 |
|  |  |  | IPR008972. | Cupredoxin. |
| CaWEPP-1DE-TIO2-239 | Uncharacterized protein Putative | D8SMH9 | IPR000941. | Enolase |
|  |  |  | IPR020810. | Enolase_C |
|  |  |  | IPR029065. | Enolase_C-like |
|  |  |  | IPR020809. | Enolase_CS |
|  |  |  | IPR020811. | Enolase_N |
|  |  |  | IPR029017. | Enolase_N_like |
| CaWEPP-IMAC-1DE1-190 | Uncharacterized protein Putative | E0CQT6 | IPR001117. | Cu-oxidase |
|  |  |  | IPR011706. | Cu-oxidase_2 |
|  |  |  | IPR011707. | Cu-oxidase_3 |
|  |  |  | IPR008972. | Cupredoxin |
| CaWEPP-IMAC-1DE1-317 | Uncharacterized protein | I1ILG3 | IPR011013. | Gal_mutarotase_SF_dom |
|  |  |  | IPR011330. | Glyco_hydro/deAcase_b/a-brl |
|  |  |  | IPR013780. | Glyco_hydro_13_b |
|  |  |  | IPR027291. | Glyco_hydro_38/57_N |
|  |  |  | IPR011682. | Glyco_hydro_38_C |
|  |  |  | IPR015341. | Glyco_hydro_38_cen |
|  |  |  | IPR000602. | Glyco_hydro_38_N |
|  |  |  | IPR028995. | Glyco_hydro_57/38_cen |
| CaWEPP-IMAC-1DE1-197 | Uncharacterized protein | I1JBG9 | IPR026891. | Fn3-like |
|  |  |  | IPR026892. | Glyco_hydro_3 |
|  |  |  | IPR002772. | Glyco_hydro_3_C |
|  |  |  | IPR001764. | Glyco_hydro_3_N |
|  |  |  | IPR017853. | Glycoside_hydrolase_SF |
| CaWEPP-1DE-TIO2-331 | Uncharacterized protein | I1K795 | IPR025661. | Pept_asp_AS |
|  |  |  | IPR000169. | Pept_cys_AS |
|  |  |  | IPR025660. | Pept_his_AS |
|  |  |  | IPR013128. | Peptidase_C1A |
|  |  |  | IPR000668. | Peptidase_C1A_C |
|  |  |  | IPR013201. | Prot_inhib_I29 |
| CaWEPP-1DE-TIO2-359 | Uncharacterized protein | I1KPN5 | IPR018181. | Heat_shock_70_CS. |
|  |  |  | IPR029048. | HSP70_C. |
|  |  |  | IPR029047. | HSP70_peptide-bd. |
|  |  |  | IPR013126. | Hsp_70_fam |
| CaWEPP-IMAC-1DE1-199 | Uncharacterized protein | I1LK37 | IPR001938. | Thaumatin |
| CaWEPP-IMAC-1DE1-53 | Uncharacterized protein | I1MY65 | IPR001117. | Cu-oxidase. |
|  |  |  | IPR011706. | Cu-oxidase_2. |
|  |  |  | IPR011707. | Cu-oxidase_3. |
|  |  |  | IPR008972. | Cupredoxin |
| CaWEPP-1DE-TIO2-360 | Uncharacterized protein | I1NWK0 | IPR018181. | Heat_shock_70_CS. |
|  |  |  | IPR029048. | HSP70_C. |
|  |  |  | IPR029047. | HSP70_peptide-bd. |
|  |  |  | IPR013126. | Hsp_70_fam |
| CaWEPP-IMAC-1DE1-396 | Uncharacterized protein | I3STK8 | IPR001480. | Bulb-type_lectin_dom |
| CaWEPP-IMAC-1DE1-496 | Uncharacterized protein | M0TDI7 | IPR029044. | Nucleotide-diphossugar_trans. |
|  |  |  | IPR004901. | RGP_fam |
| CaWEPP-IMAC-1DE1-336 | Uncharacterized protein | M0UTH0 | IPR008758. | Peptidase_S28 |
| CaWEPP-1DE-TIO2-186 | Uncharacterized protein | M0Z6C2 | IPR001557. | L-lactate/malate_DH. |
|  |  |  | IPR022383. | Lactate/malate_DH_C. |
|  |  |  | IPR001236. | Lactate/malate_DH_N. |
|  |  |  | IPR015955. | Lactate_DH/Glyco_Ohase_4_C. |
|  |  |  | IPR010097. | Malate_DH_type1. |
|  |  |  | IPR016040. | NAD(P)-bd_dom |
| CaWEPP-1DE-TIO2-235 | Uncharacterized protein | M0ZHT9 | IPR018181. | Heat_shock_70_CS. |
|  |  |  | IPR029048. | HSP70_C. |
|  |  |  | IPR029047. | HSP70_peptide-bd. |
|  |  |  | IPR013126. | Hsp_70_fam |
| CaWEPP-1DE-TIO2-232 | Uncharacterized protein | M0ZI19 | IPR018181. | Heat_shock_70_CS. |
|  |  |  | IPR029048. | HSP70_C. |
|  |  |  | IPR029047. | HSP70_peptide-bd. |
|  |  |  | IPR013126. | Hsp_70_fam |
| CaWEPP-1DE-TIO2-315 | Uncharacterized protein | M4C8V0 | IPR029044. | Nucleotide-diphossugar_trans. |
|  |  |  | IPR004901. | RGP_fam |
| CaWEPP-IMAC-1DE1-340 | Uncharacterized protein | M4F1J7 | IPR001117. | Cu-oxidase. |
|  |  |  | IPR011706. | Cu-oxidase_2. |
|  |  |  | IPR011707. | Cu-oxidase_3. |
|  |  |  | IPR008972. | Cupredoxin |
| CaWEPP-IMAC-1DE1-445 | Uncharacterized protein | M4F448 | IPR001487. | Bromodomain. |
|  |  |  | IPR027353. | NET_dom. |
|  |  |  | IPR001564. | Nucleoside_diP_kinase. |
|  |  |  | IPR023005. | Nucleoside_diP_kinase_AS |
| CaWEPP-1DE-TIO2-357 | Uncharacterized protein | M5WES8 | IPR018181. | Heat_shock_70_CS. |
|  |  |  | IPR029048. | HSP70_C. |
|  |  |  | IPR029047. | HSP70_peptide-bd. |
|  |  |  | IPR013126. | Hsp_70_fam |
| CaWEPP-IMAC-1DE3-90 | Uncharacterized protein | M5XBY3 | IPR001360. | Glyco_hydro_1. |
|  |  |  | IPR018120. | Glyco_hydro_1_AS. |
|  |  |  | IPR013781. | Glyco_hydro_catalytic_dom. |
|  |  |  | IPR017853. | Glycoside_hydrolase_SF |
| CaWEPP-1DE-TIO2-302 | Uncharacterized protein | R0I2R4 | IPR029044. | Nucleotide-diphossugar_trans. |
|  |  |  | IPR004901. | RGP_fam |
| CaWEPP-IMAC-1DE1-338 | Uncharacterized protein | R0IFZ2 | IPR001117. | Cu-oxidase. |
|  |  |  | IPR011706. | Cu-oxidase_2. |
|  |  |  | IPR011707. | Cu-oxidase_3. |
|  |  |  | IPR008972. | Cupredoxin |
| CaWEPP-IMAC-1DE1-307 | Uncharacterized protein | U5DB03 | IPR009410. | Allene_ox_cyc |
| CaWEPP-IMAC-1DE1-411 | Uncharacterized protein | V4KXG2 | IPR001117. | Cu-oxidase. |
|  |  |  | IPR011706. | Cu-oxidase_2. |
|  |  |  | IPR011707. | Cu-oxidase_3. |
|  |  |  | IPR008972. | Cupredoxin |
| CaWEPP-IMAC-1DE1-118 | Uncharacterized protein | V4L4D3 | IPR001117. | Cu-oxidase. |
|  |  |  | IPR011706. | Cu-oxidase_2. |
|  |  |  | IPR011707. | Cu-oxidase_3. |
|  |  |  | IPR008972. | Cupredoxin |
| CaWEPP-IMAC-1DE1-71 | Uncharacterized protein | V4SL94 | IPR001117. | Cu-oxidase. |
|  |  |  | IPR011706. | Cu-oxidase_2. |
|  |  |  | IPR011707. | Cu-oxidase_3. |
|  |  |  | IPR008972. | Cupredoxin |
| CaWEPP-1DE-TIO2-129 | Uncharacterized protein | V4UFP7 | IPR001360. | Glyco_hydro_1. |
|  |  |  | IPR013781. | Glyco_hydro_catalytic_dom. |
|  |  |  | IPR017853. | Glycoside_hydrolase_SF |
|  |  |  | IPR019793. | Peroxidases_heam-ligand_BS |
| CaWEPP-IMAC-1DE1-126 | Uncharacterized protein | V7CUM3 | IPR001117. | Cu-oxidase. |
|  |  |  | IPR011706. | Cu-oxidase_2. |
|  |  |  | IPR011707. | Cu-oxidase_3. |
|  |  |  | IPR008972. | Cupredoxin. |
| CaWEPP-IMAC-1DE1-327 | Uncharacterized protein | W1PAM9 | IPR001360. | Glyco_hydro_1. |
|  |  |  | IPR013781. | Glyco_hydro_catalytic_dom. |
|  |  |  | IPR017853. | Glycoside_hydrolase_SF. |
| CaWEPP-IMAC-1DE1-259 | Uncharacterized protein | W1PJH3 | IPR001938. | Thaumatin |
| CaWEPP-IMAC-1DE1-370 | Uncharacterized protein | W1PNY8 | IPR001611. | Leu-rich_rpt. |
|  |  |  | IPR013210. | LRR-contain_N2 |
| CaWEPP-1DE-TIO2-293 | Uncharacterized protein | W5GCN1 | IPR001344. | Chloro_AB-bd_pln. |
|  |  |  | IPR022796. | Chloroa_b-bind. |
|  |  |  | IPR023329. | Chlorophyll_a/b-bd_dom |

^a^Sample origin based on technique used. The first letters (Ca) signify the source plant, *Cicer arietinum*, followed by WEPP denotes WR 315 Extracellular Matrix Phosphoproteome.

^b^Gene identification number as in GenBank.

^c^InterPro domain accession number.

For each of the proteins identified as “unknown functions” in Supplementary TableS2 the InterPro site was queried for domains in SMART, Panther, and Pfam databases to identify functional domains of each protein. N/F, not found; N/A, not applicable.
