# Supplementary material for: Combining extracellular matrix proteome and phosphoproteome of chickpea and meta‐analysis reveal novel proteoforms and evolutionary significance of clade‐specific wall‐associated events in plant
Source: Plant Direct. 2024 Mar 18;8(3):e572. doi: 10.1002/pld3.572 (PMC10945595; doi:10.1002/pld3.572)
Supplement: Supplementary file 11 — Figure S1. Purity assessment of isolated ECM protein from chickpea seedlings. (a). Transmission electron micrographs of purified ECM. (b) Glucose 6 phosphate dehydrogenase activity Bars indicate activity in crude and ECM protein extract. (c) Catalase assay in ECM protein and crude fractions (d) Vanadate sensitive H+ ATPase activity in ECM protein, crude extract and plasma membrane protein. Figure S2. Detailed workflow of (a) Comparative proteomics and phosphoproteomics study. ECM proteins were extracted from chickpea seedlings. Number of seedlings is indicated by n. Non‐redundant set of proteins was catalogued according to gene ontology using Blast2GO program for (b) Biological process and (c) molecular function. Pie chart showing functional categorization (d), ECM protein and (e) ECM phosphoprotein. Figure S3. Expansion of plant ECM protein families. (a) CW‐known unknown proteins from phanerogamia and crypogamia B proteins related to lipid metabolism. Each row represents a taxonomic species and each column represents a organ/combination of organs showing expansion. Names of organs are given in red, whereas categories of ECM protein families are in different colored corrplot. The colour intensity and size of the circles are proportional to the number of protein falling under the given protein family. (b) Proteins related to lipid metabolism. Abbreviation: L, leaf; S, stem; R, root; CS, cell suspension; H, hypocotyl; F, fruit; I, inflorescence; SD, seed; SDN, seedling; L‐S‐R(V), vegetative. Figure S4. Phylogenetic tree analysis of orthologous and paralogous ECM cluster proteins. Figure S5. dN/dS (ka/Ks) tree of (a) Berberine; (b) CaZY; (c) Class 1 fascilin; (d) Class 2 fascilin; (e), Class 3 fascilin; (f), Glycine rich protein. Figure S6. dN/dS (Ka/Ks) tree of (a), Direngent; (b) Germin; (c) Expansin; (d) Phi starvation protein; (e) Extensin; (f) Phosphatase. Figure S7. dN/dS (ka/Ks) tree of (a) Lectin; (b), Proteinase inhibitor (c) LRR protein; (d) Purple acid phosp [file PLD3-8-e572-s007.pdf]

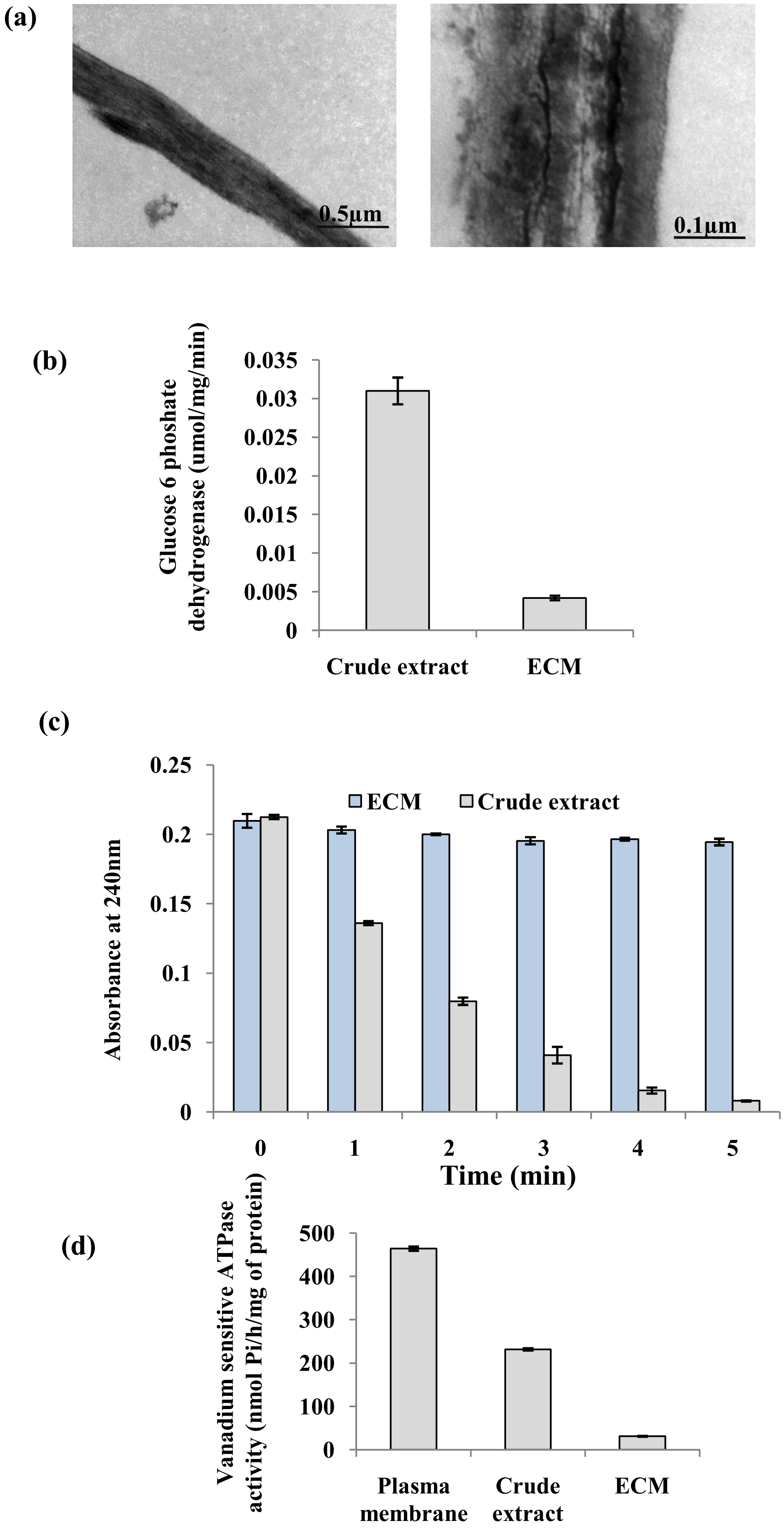

FIGURE S1. Purity assessment of isolated ECM protein from chickpea seedlings. (a). Transmission electron micrographs of purified ECM. (b) Glucose 6 phoshate dehydrogenase activity Bars indicate activity in crude and ECM protein extract. (c) Catalase assay in ECM protein and crude fractions (d) Vanadate sensitive H+ ATPase activity in ECM protein , crude extract and plasma membrane protein.

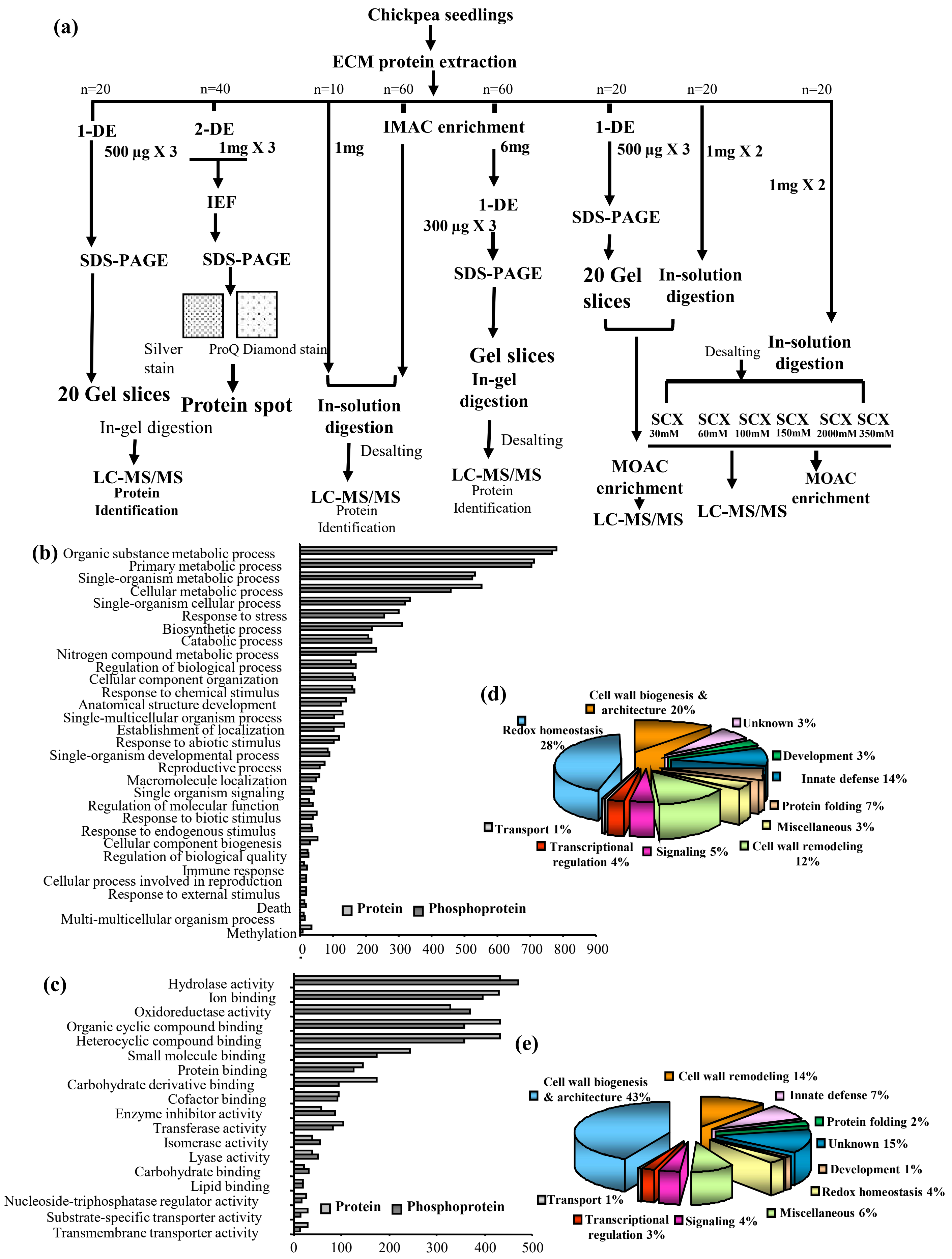

FIGURE S2. Detailed workflow of (a) Comparative proteomics and phosphoproteomics study. ECM proteins were extracted from chickpea seedlings. Number of seedlings is indicated by n. Non-redundant set of proteins was catalogued according to gene ontology using Blast2GO program for (b) Biological process and (c) molecular function. Pie chart showing functional categorization (d), ECM protein and (e) ECM phosphoprotein.

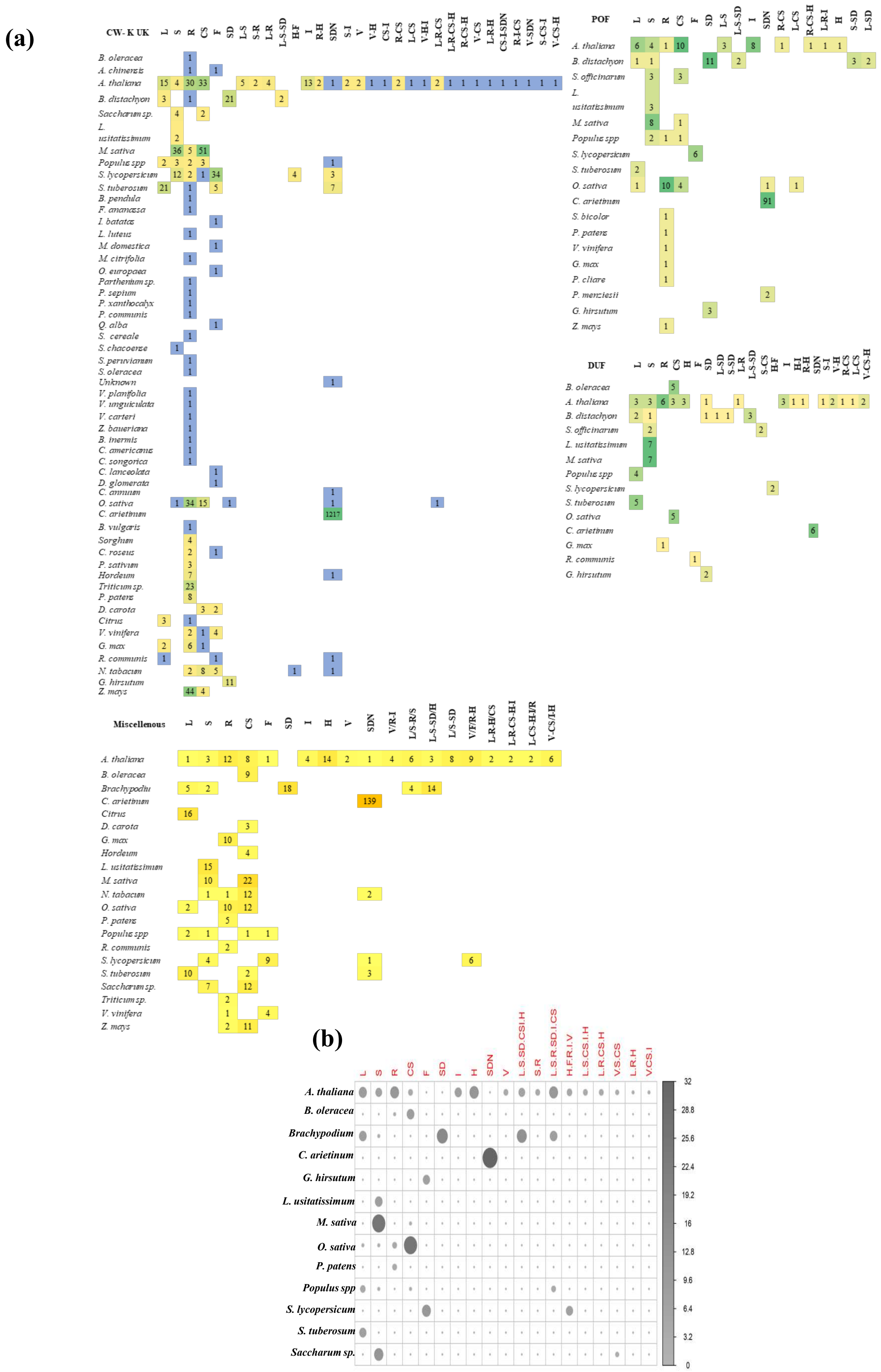

FIGURE S3. Expansion of plant ECM protein families. (a) CW-known unknown proteins from phanerogamia and crypogamia B proteins related to lipid metabolism. Each row represents a taxonomic species and each column represents a organ/combination of organs showing expansion. Names of organs are given in red, whereas categories of ECM protein families are in different colored corrplot. The colour intensity and size of the circles are proportional to the number of protein falling under the given protein family. (b) Proteins related to lipid metabolism. Abbreviation: L, leaf; S, stem; R, root; CS, cell suspension; H, hypocotyl; F, fruit; I, inflorescence; SD, seed; SDN, seedling; L-S-R(V), vegetative.

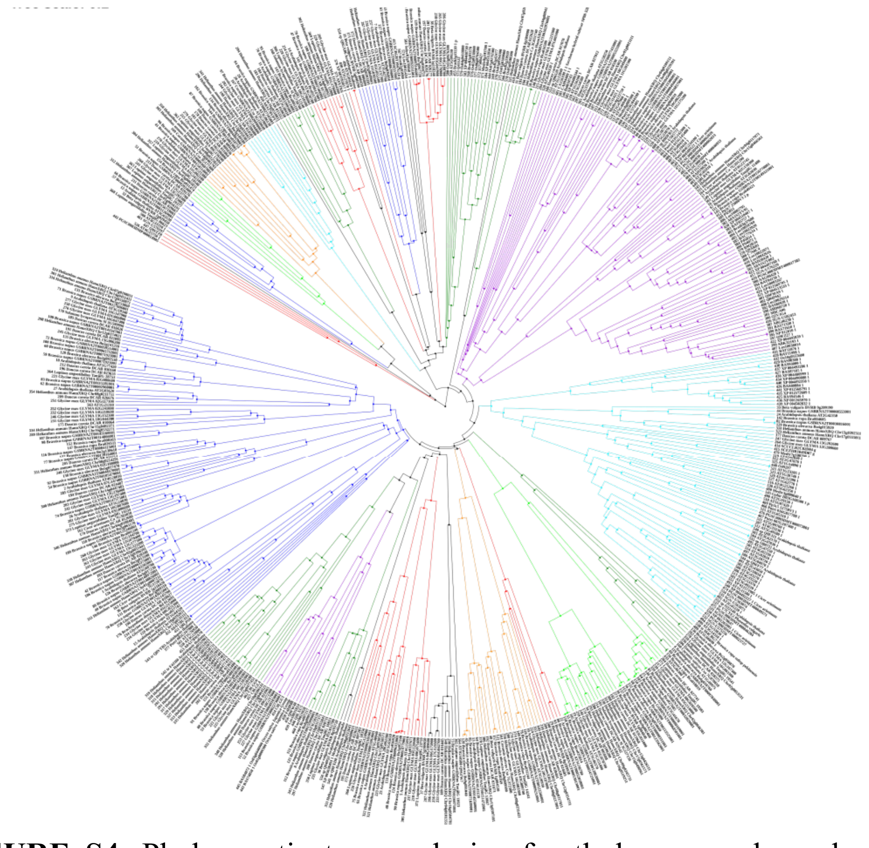

FIGURE S4. Phylogenetic tree analysis of orthologous and paralogous ECM cluster proteins.

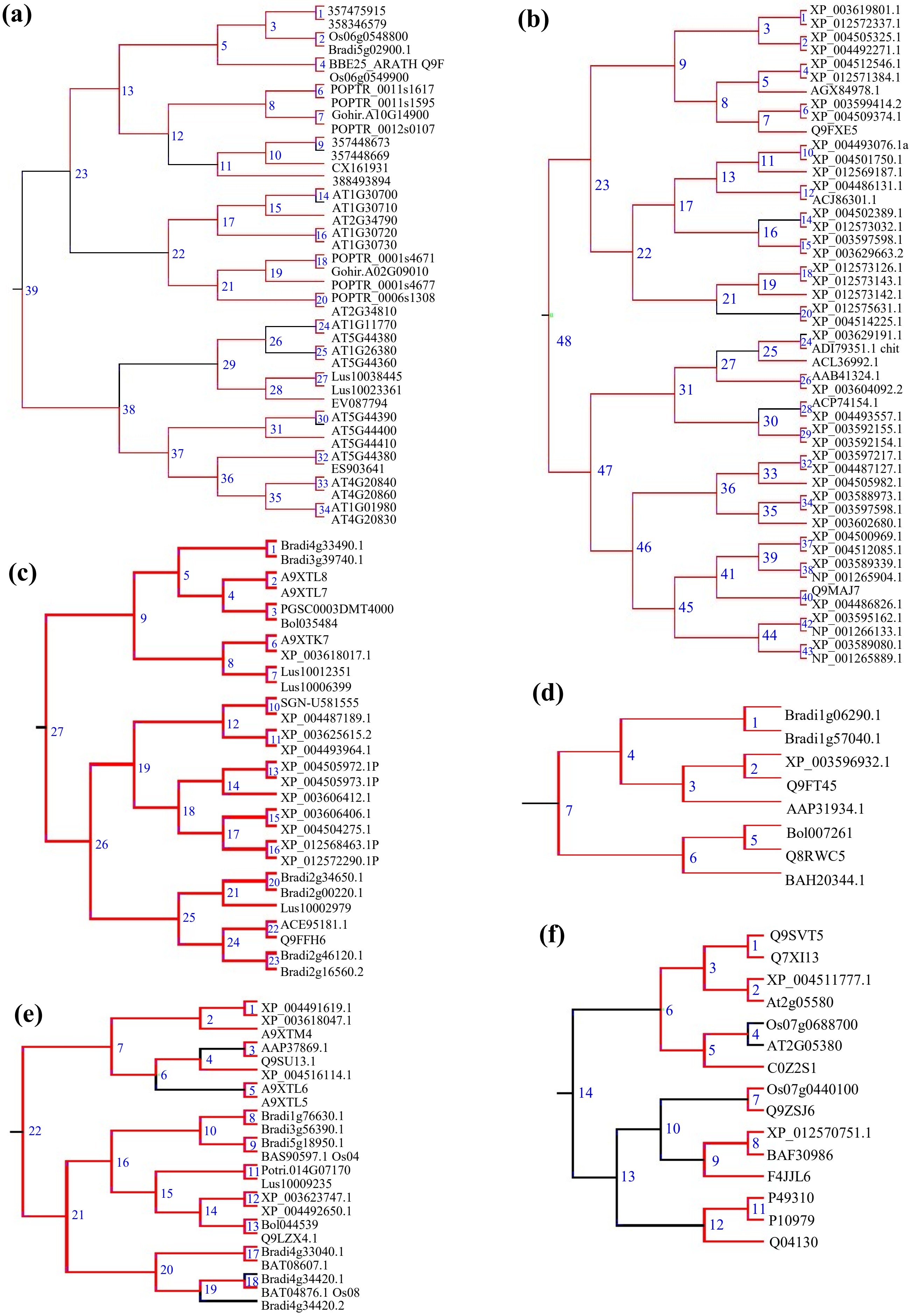

FIGURE S5. dN/dS (ka/Ks) tree of (a) Berberine; (b) CaZY; (c) Class 1 fascilin; (d) Class 2 fascilin; (e), Class 3 fascilin ; (f), Glycine rich protein.

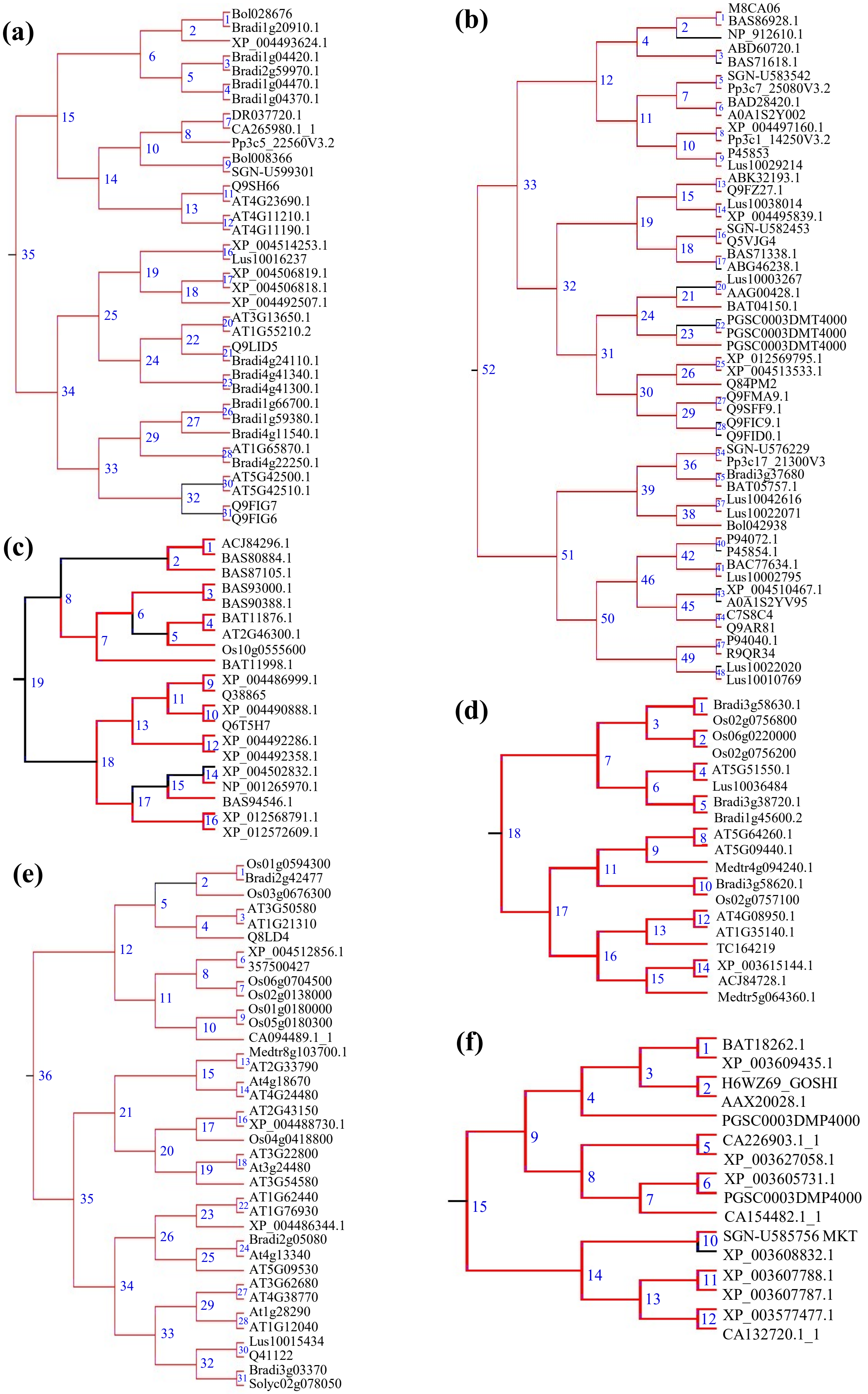

FIGURE S6. dN/dS (Ka/Ks) tree of (a), Direngent; (b) Germin; (c) Expansin; (d) Phi starvation protein; (e) Extensin; (f) Phosphatase.

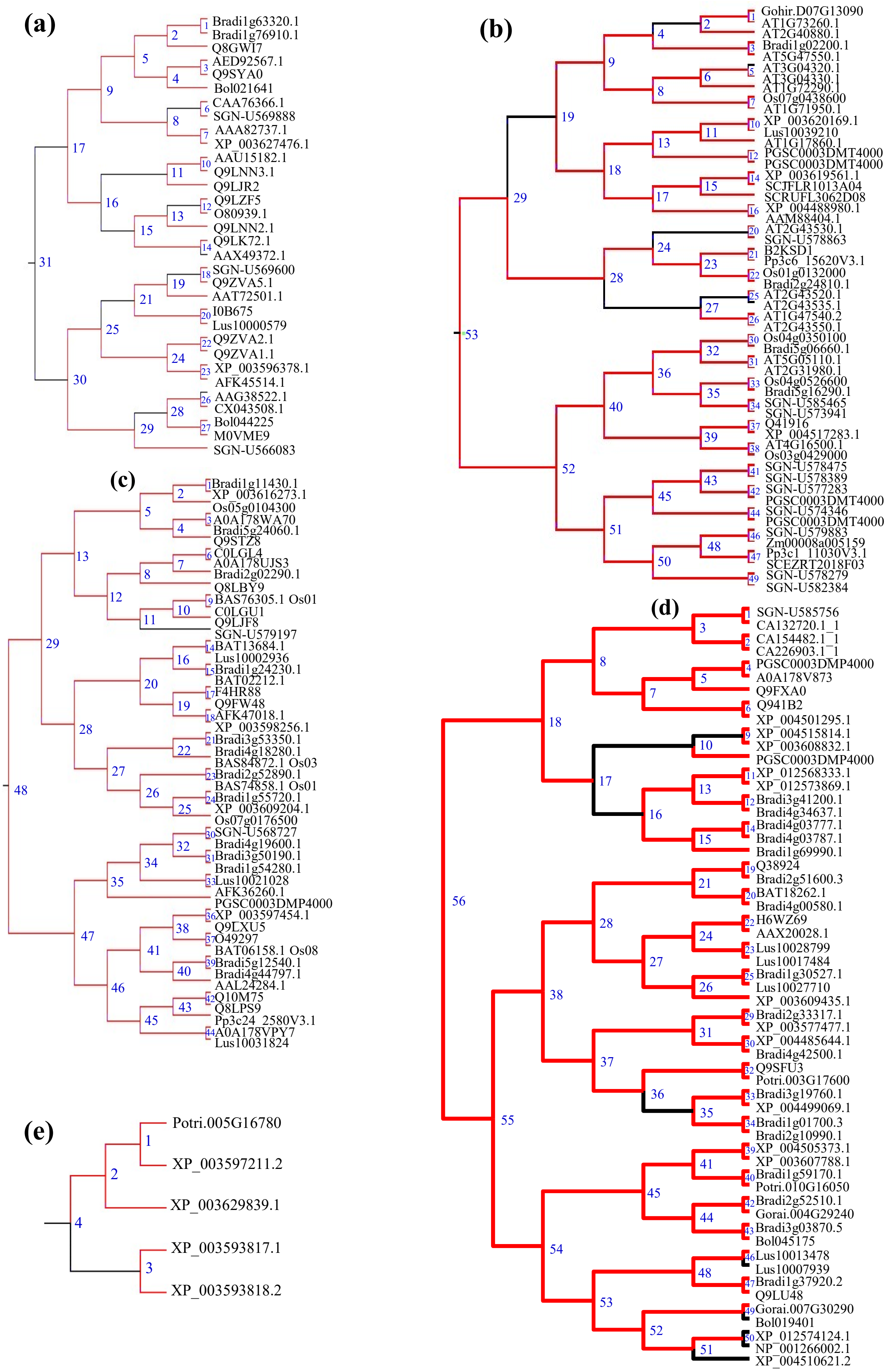

FIGURE S7. dN/dS (ka/Ks) tree of (a) Lectin; (b), Proteinase inhibitor (c) LRR protein; (d) Purple acid phosphatase; (e) Fascilin

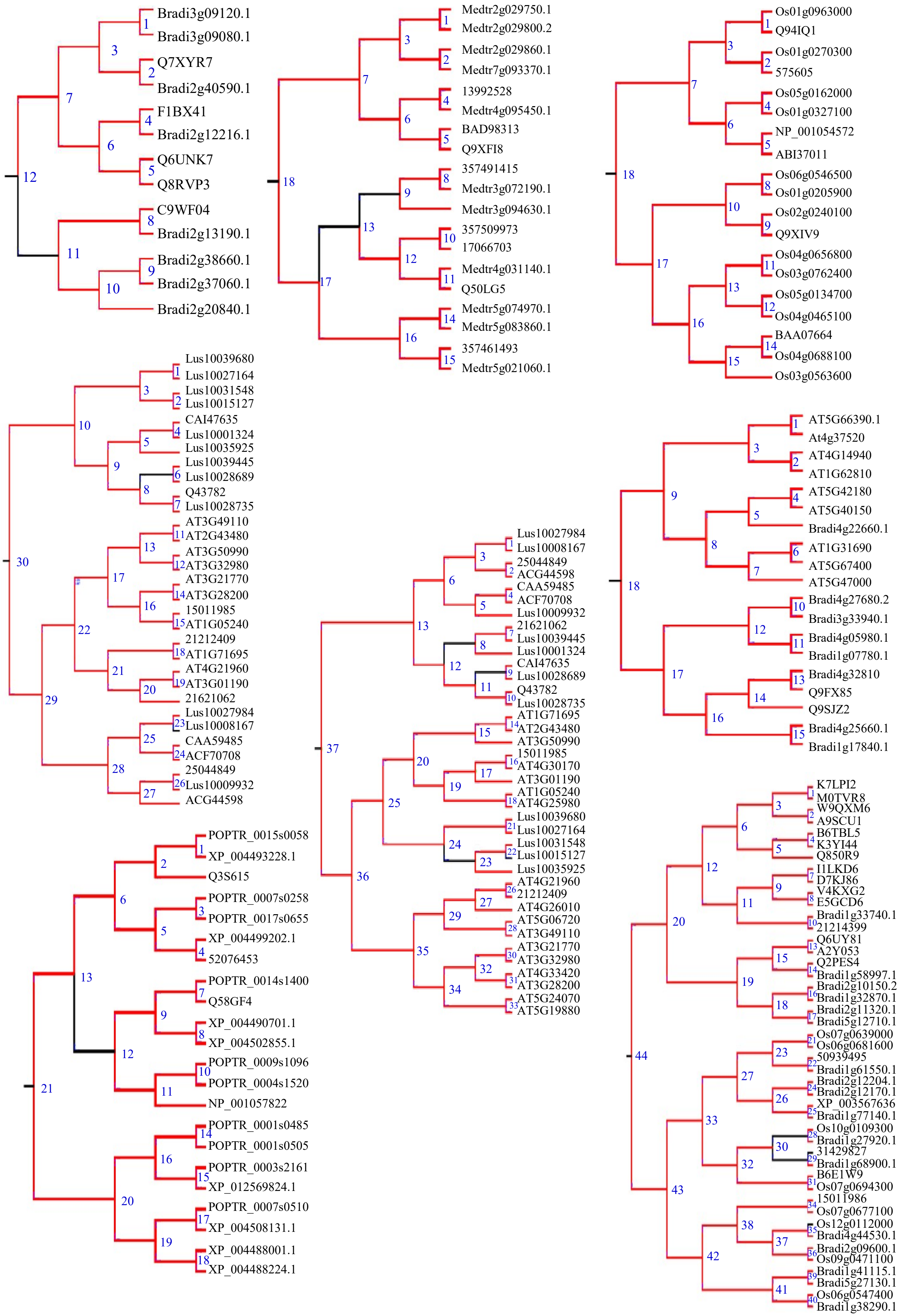

FIGURE S8. dN/dS (ka/Ks) tree of Peroxidase

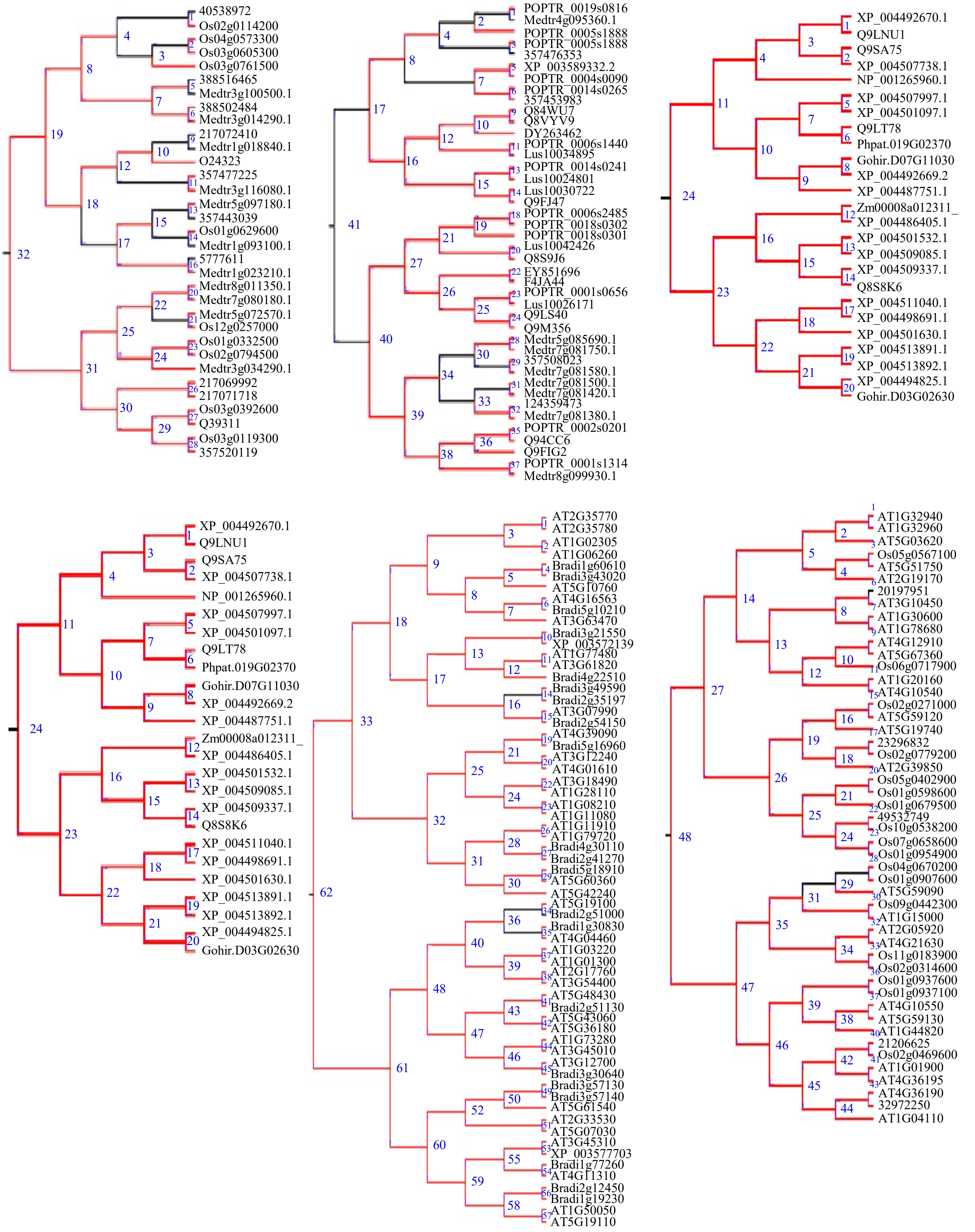

FIGURE S9. dN/dS (ka/Ks) tree of Protease

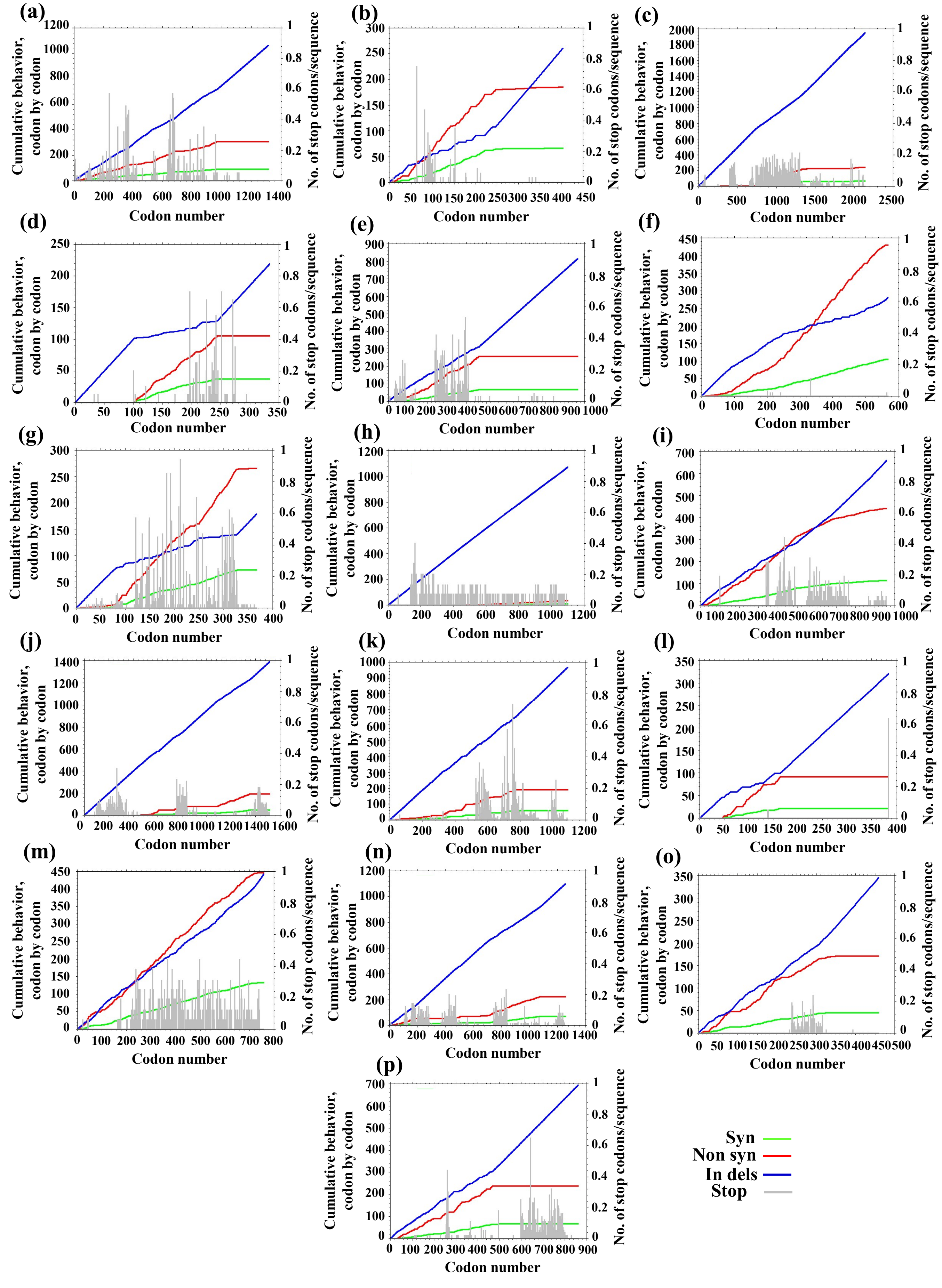

FIGURE S10. Analysis of synonymous, non-synonymous and indels in ECM proteins (a) Berberine. (b) Direngent protein. (c) CaZY. (d) Expansin. (e) Extensin. (f) Fascilin. (g) Germin. (h) Glycine rich protein. (i) Lectin. (j) LRR protein. (k) Peroxidase. (l) Phi starvation protein. (m) Phosphatase. (n) Protease. (o) Proteinase inhibitor. (p) Purple acid phosphatase.

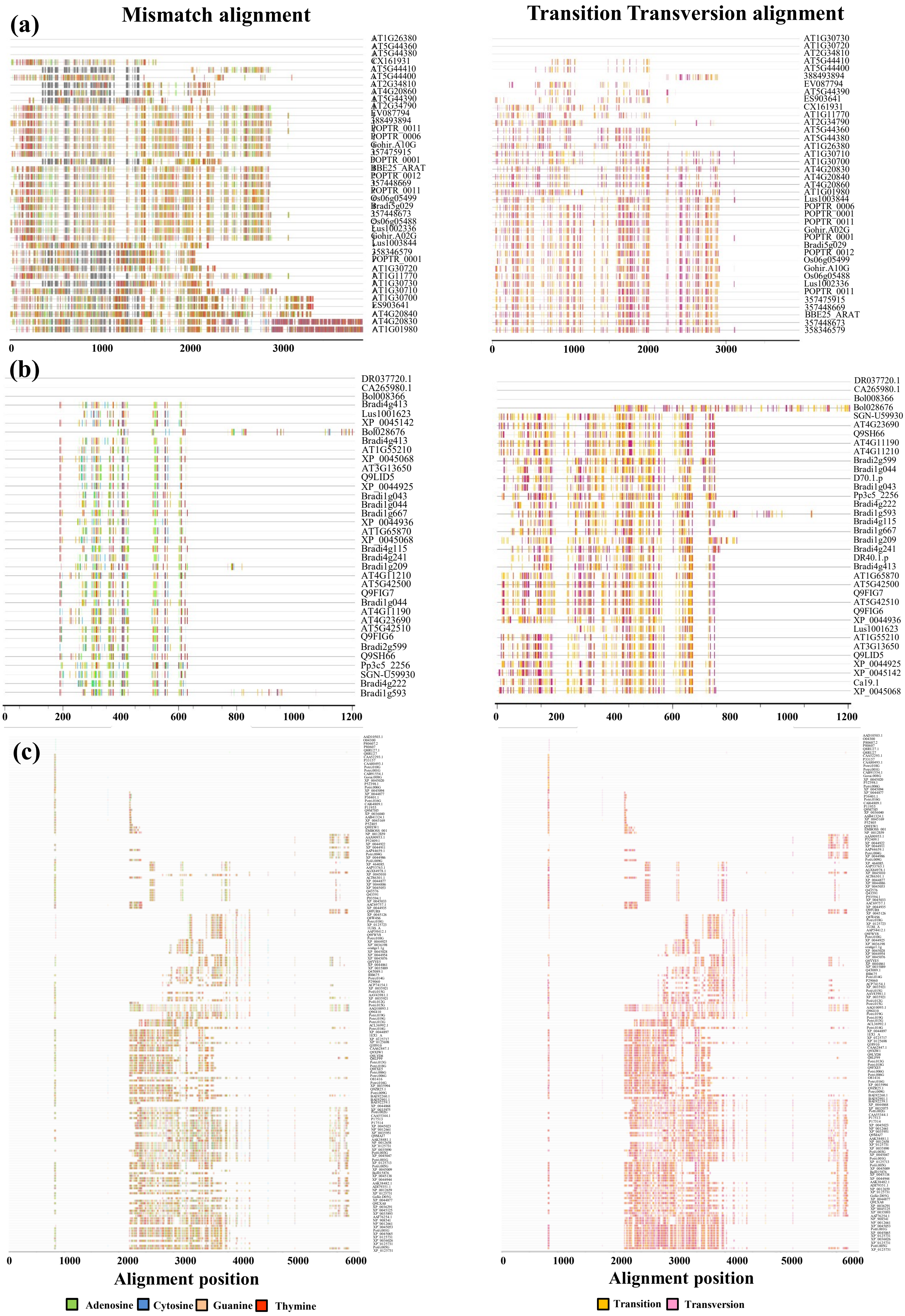

FIGURE S11. Mismatch and transition-transversion alingnment of (a) Berberine, (b) Direngent protein and (c) CaZY.

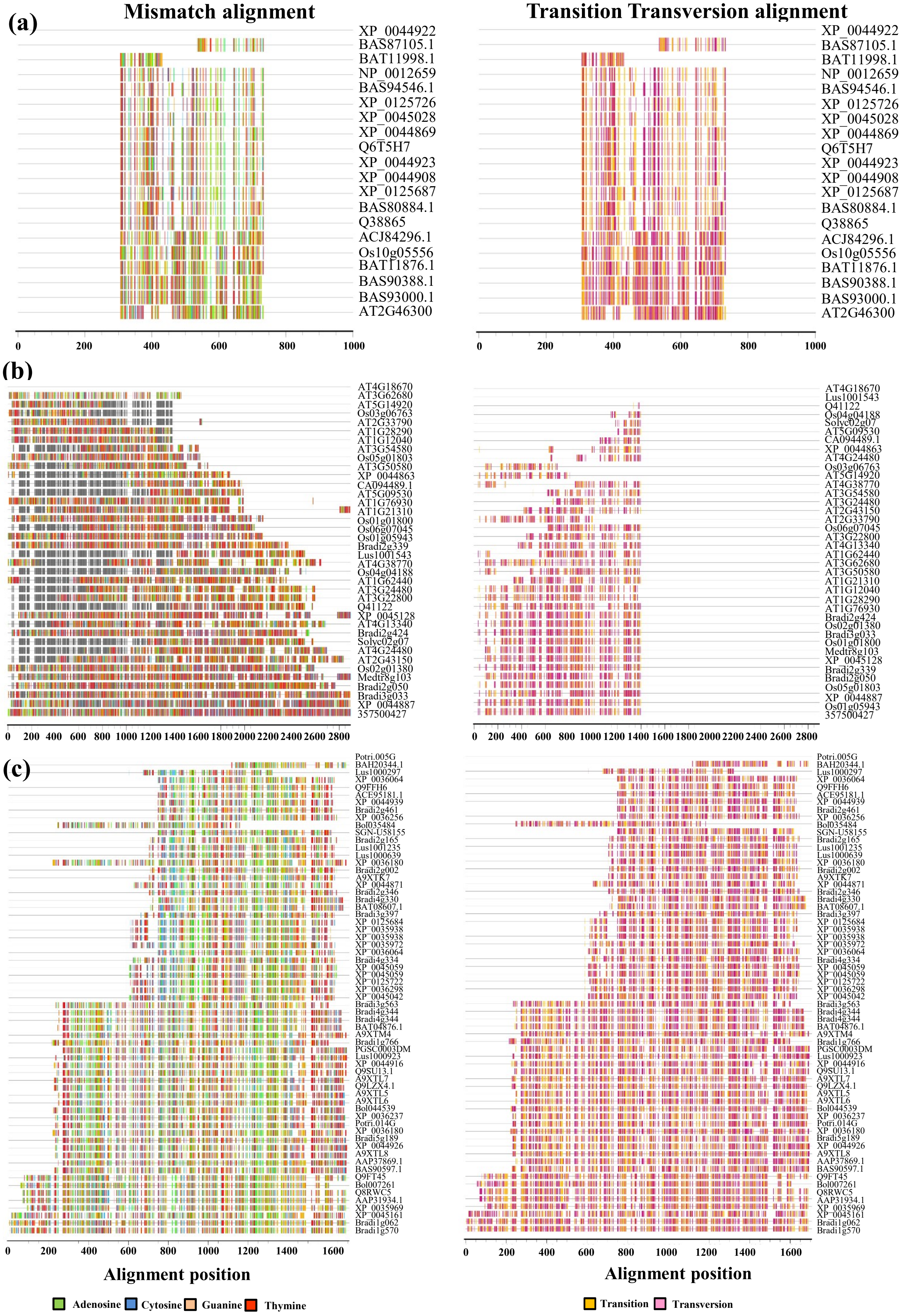

FIGURE S12. Mismatch and transition-transversion alingnment of (a) Expansin, (b) Extensin and © Fascilin arabinogalactan protein

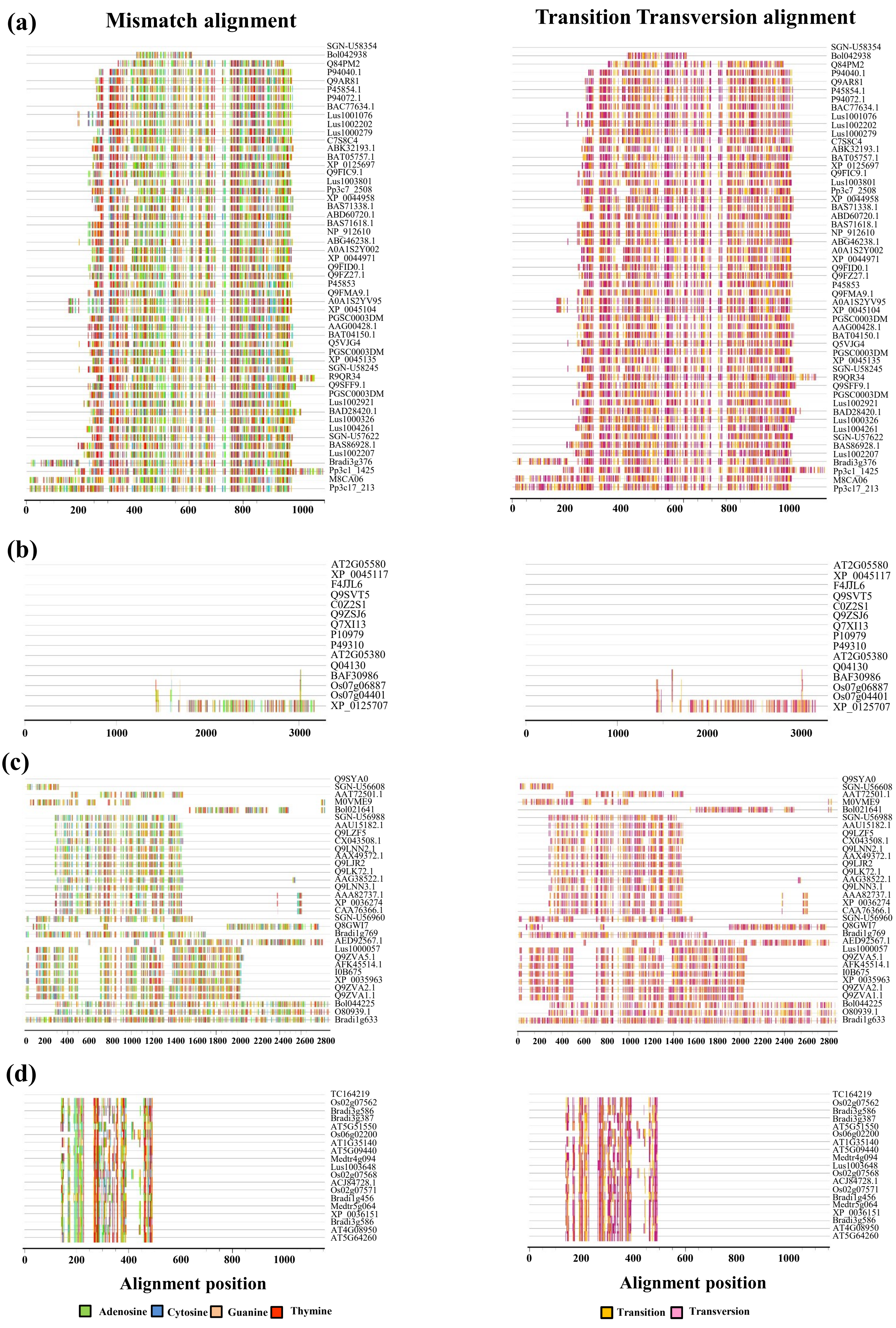

FIGURE S13. Mismatch and transition-transversion alingnment of (a) Germin, (b) Glycine rich, (c) Lectin, and (d) Phi starvation protein.

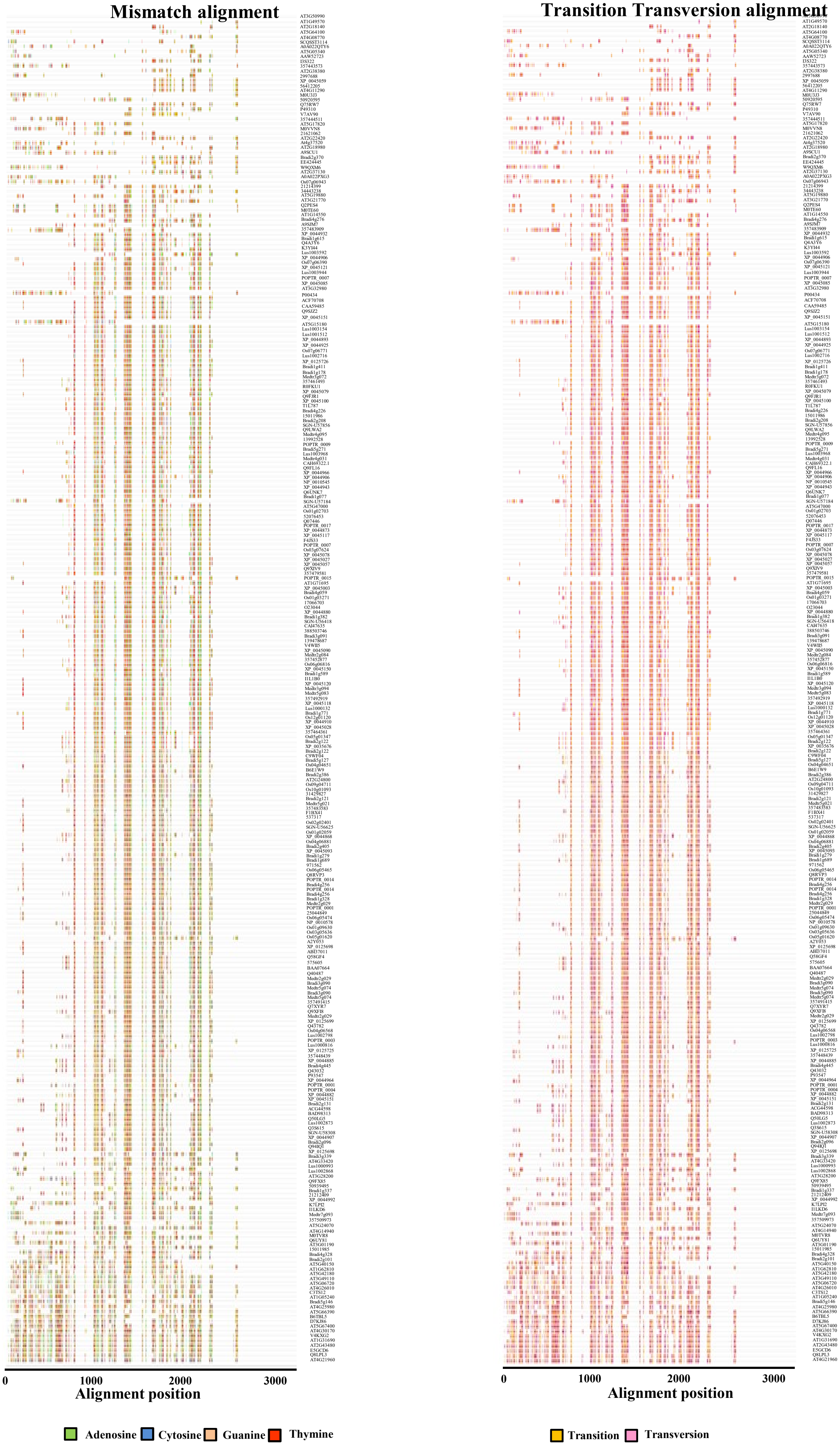

FIGURE S14. Mismatch and transition-transversion alingnment of peroxidase

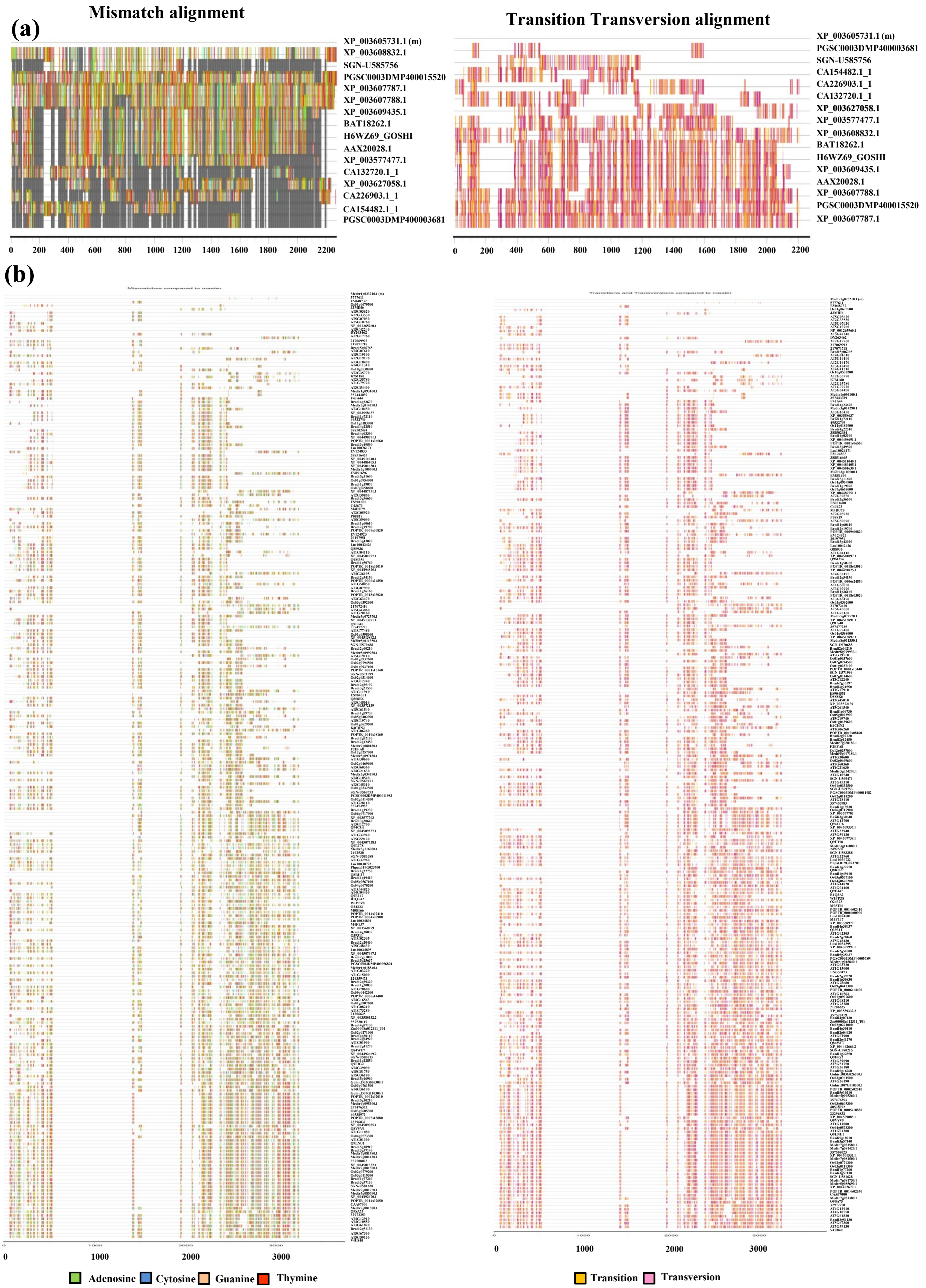

FIGURE S15. Mismatch and transition-transversion alingnment of (a) phosphatase, (b) protease.

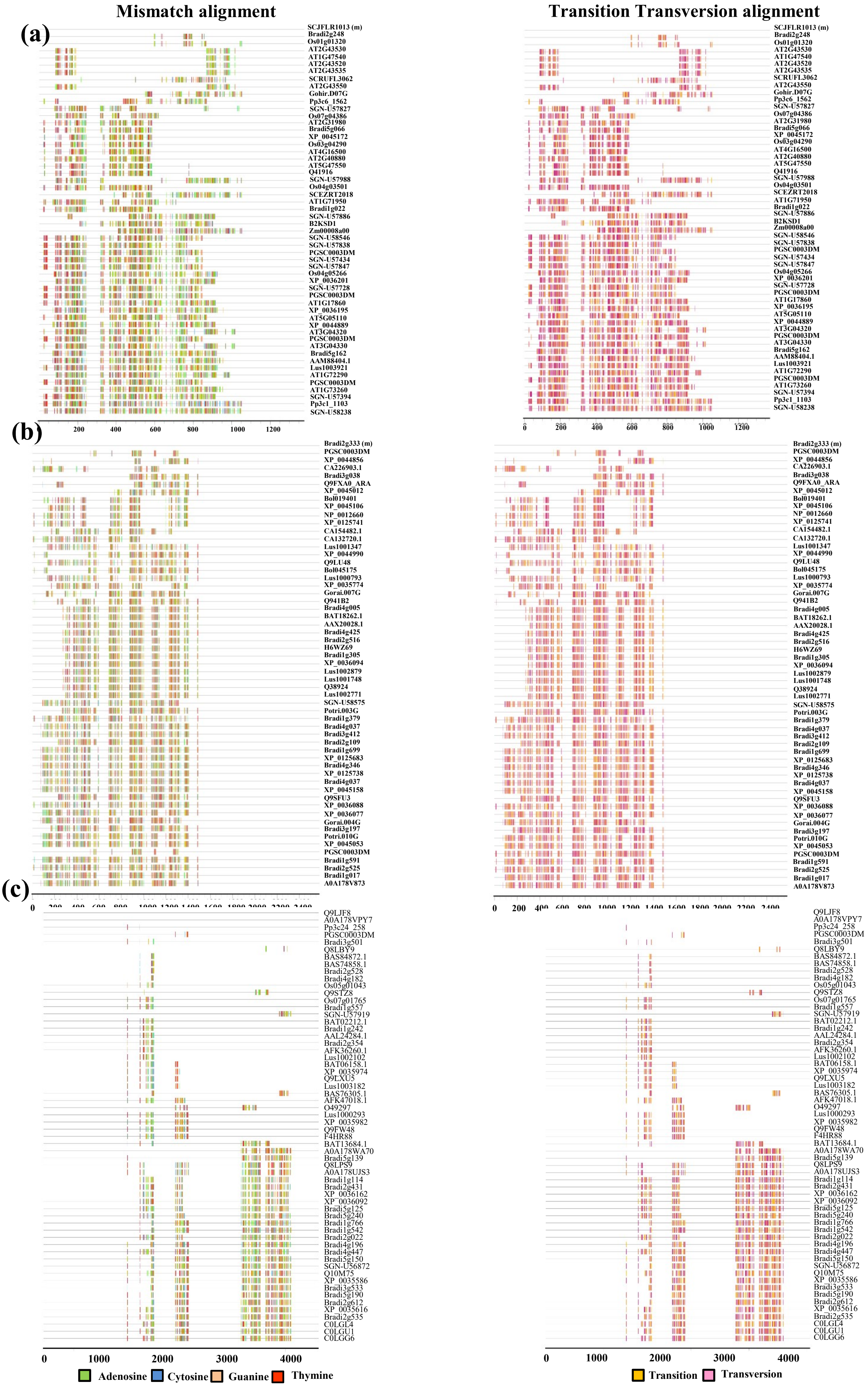

FIGURE S16. Mismatch and transition-transversion alignment of (a) proteinase inhibitors, (b) purple acid phosphatase, (c) LRR

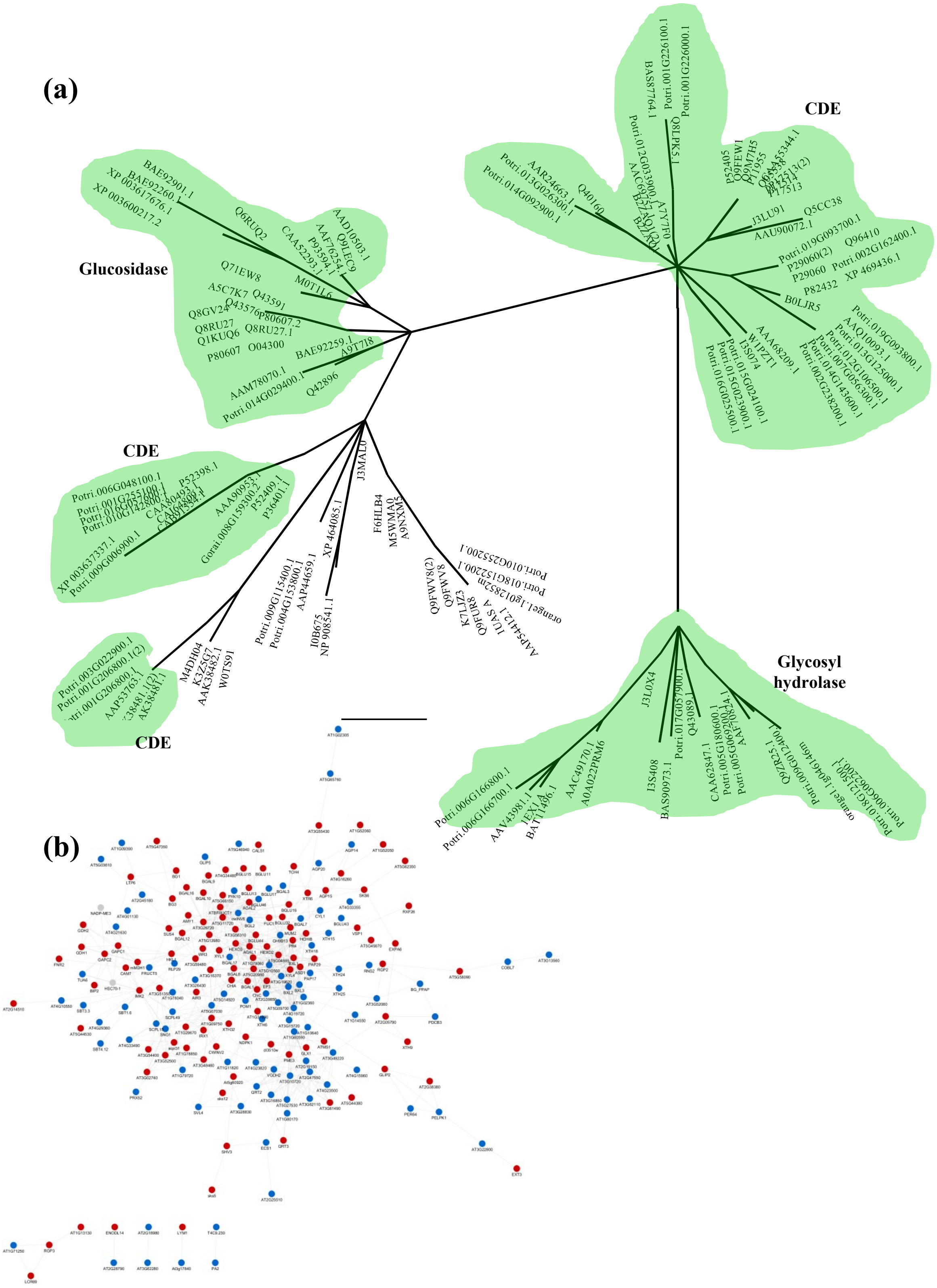

FIGURE S17. Evolution and interaction of wall enzyme machinery based on plant matrisome. Evolutionary relationship of identified (a) wall enzyme machinery. (b) Network analysis depicting correlation among identified wall enzymes. Each node represents a given ECM protein associated based on top BLAST hit against SwissProt and an edge denotes a probability of two given proteins (nodes) potentially interacting based on the cytoprophet algorithm. Nodes and edges represent ECM proteins and correlation between proteins, respectively. Abbreviation: CDE, carbohydrade degrading enzyme

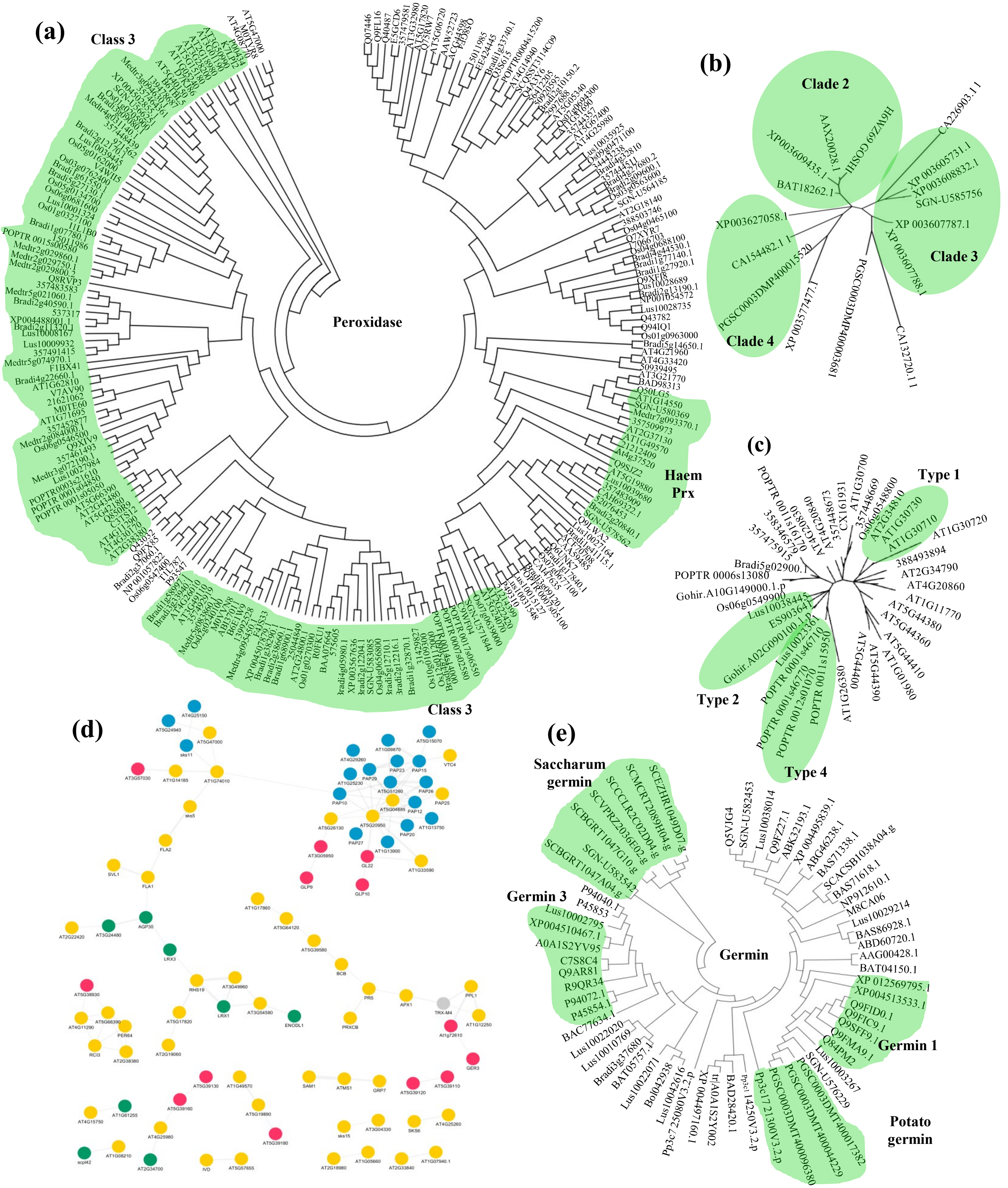

FIGURE S18. Evolution and interaction of wall organization component based on plant matrisome. Evolutionary relationship of identified (a) Peroxidase, (b) Purple acid phosphatase, (c) Berberine, (d) Germin, (e) Network analysis depicting correlation among identified wall enzymes. Nodes and edges represent ECM proteins and correlation between proteins, respectively.

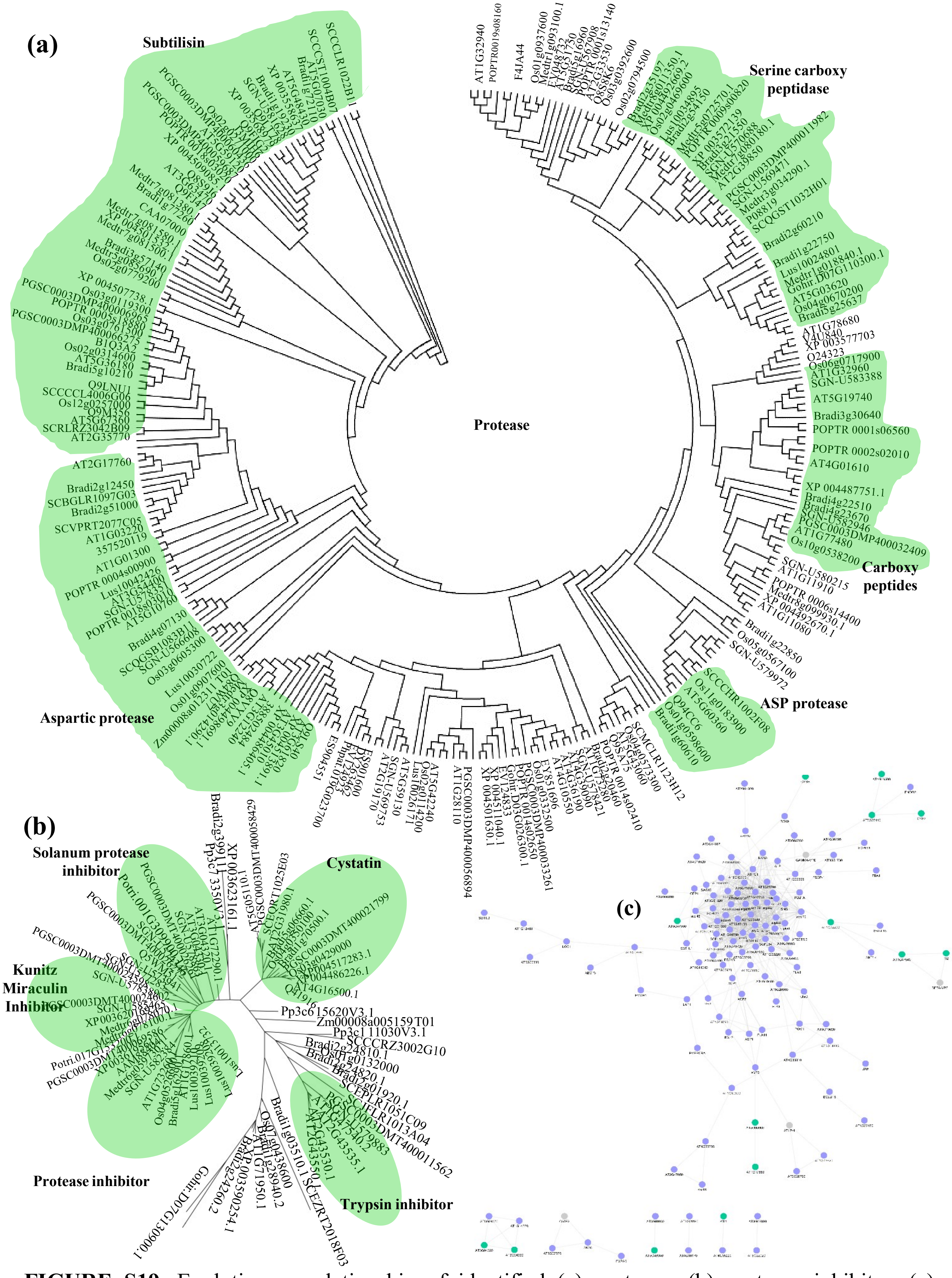

FIGURE S19. Evolutionary relationship of identified (a) protease, (b) protease inhibitor, (c) Network analysis depicting correlation among identified wall protein homeostasis machinery. Nodes and edges represent ECM proteins and correlation between proteins, respectively.

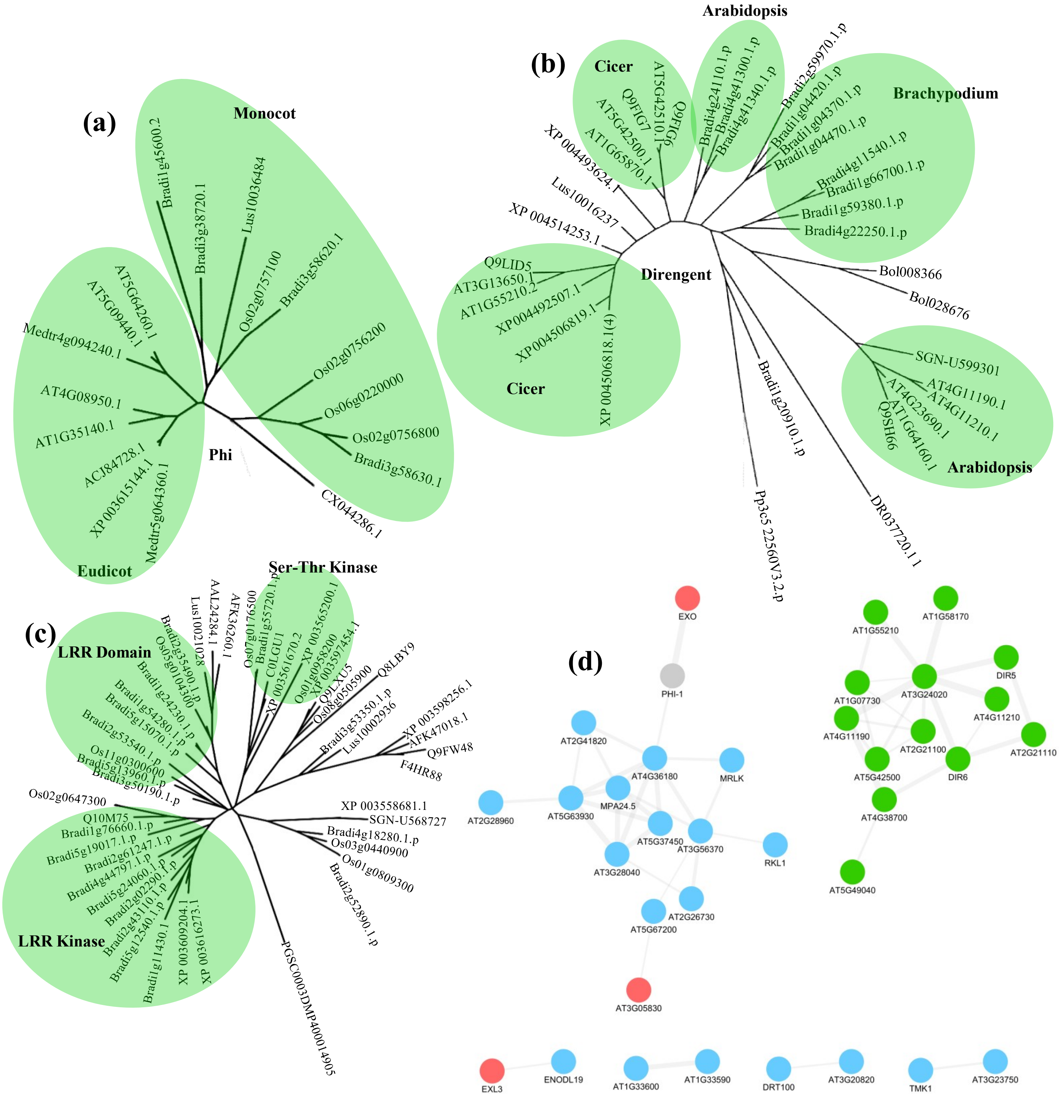

FIGURE S20. Evolutionary relationship of identified (a) Phi, (b) LRR, (c) Direngent protein, (d) Network analysis depicting correlation among identified miscellaneous protein. Nodes and edges represent ECM proteins and correlation between proteins, respectively.

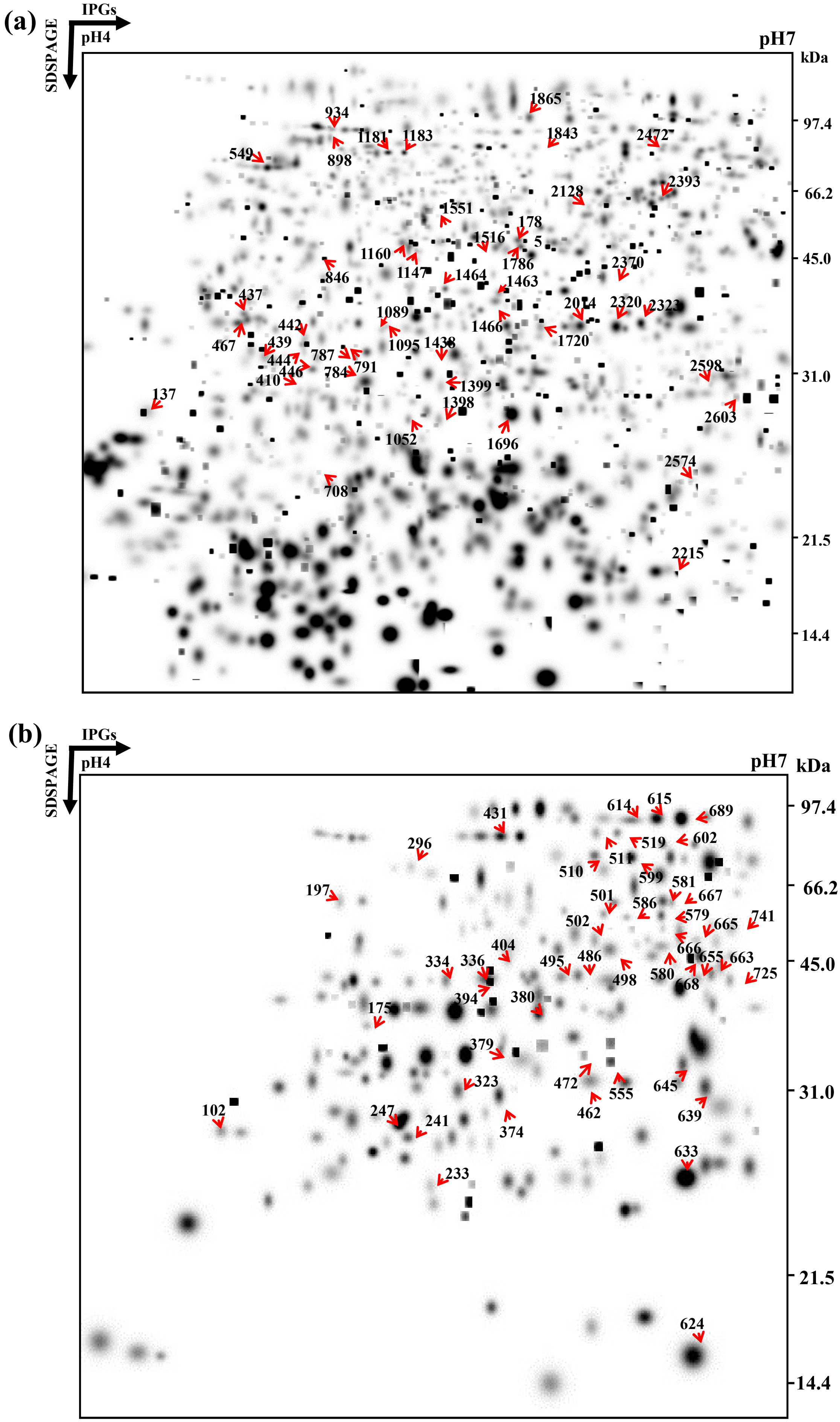

FIGURE S21. 2-DE analysis and spot detection. (a) ECM proteins and (b) ECM phosphoproteins were zoomed onto 24 cm IPG strip (pH 4-7) and second dimension was performed on 12.5% (w/v) SDS-PAGE.
