# Supplementary material for: Combining extracellular matrix proteome and phosphoproteome of chickpea and meta‐analysis reveal novel proteoforms and evolutionary significance of clade‐specific wall‐associated events in plant
Source: Plant Direct. 2024 Mar 18;8(3):e572. doi: 10.1002/pld3.572 (PMC10945595; doi:10.1002/pld3.572)
Supplement: Supplementary file 13 — Data S2 Supporting Information [file PLD3-8-e572-s012.pdf]

Supplementary Material Reference

Basu, U., Francis, J.L., Whittal, R.W., Stephens, J.L., Wang, Y. & Zaiane, O.R. et al. (2006) Extracellular proteomes of Arabidopsis thaliana and Brassica napus roots: analysis and comparison by MUdPIT and LC-MS/MS. *Plant and Soil,* 286, 357-376.

Bayer, E.M., Bottrill, A.R., Walshaw, J., Vigouroux, M., Naldrett, M.J. & Thomas, C.L. et al. (2006) Arabidopsis cell wall proteome defined using multidimensional protein identification technology. *Proteomics,* 6, 301-311.

Bengtsson, T., Weighill, D., Proux-Wéra, E., Levander F. & Resjo, S. et al. (2014) Proteomics and transcriptomics of the BABA-induced resistance response in potato using a novel functional annotation approach. *BMC Genomics* 15, 315.

Bhushan, D., Jaiswal, D.K., Ray, D., Basu, D., Datta, A. & Chakraborty S. et al. (2011) Dehydration-responsive reversible and irreversible changes in the extracellular matrix: comparative proteomics of chickpea genotypes with contrasting tolerance. *Journal of Proteome Research,* 10, 2027-2046.

Bhushan, D., Pandey, A., Chattopadhyay, A., Choudhary, M.K., Chakraborty, S. & Datta, A. et al. (2006). Extracellular Matrix Proteome of Chickpea (Cicerarietinum L.) Illustrates Pathway Abundance, Novel Protein Functions and Evolutionary Perspect. *Journal of Proteome Research,* 5, 1711-1720.

Bhushan, D., Pandey, A., Choudhary, M.K., Datta, A., Chakraborty, S. & Chakraborty, N. (2007) Comparative proteomics analysis of differentially expressed proteins in chickpea extracellular matrix during dehydration stress. *Molecular and Cellular Proteomics,* 6, 1868 -1884.

Blee, K.A., Wheatley, E.R., Bonham, V.A., Mitchell, G.P., Robertson, D. & Slabas, A.R. et al. (2001) Proteomic analysis reveals a novel set of cell wall proteins in a transformed tobacco cell culture that synthesises secondary walls as determined by biochemical and morphological parameters. *Planta,* 212, 404-415.

Borderies, G., Jamet, E., Lafitte, C., Rossignol, M., Jauneau, A. & Boudart, G. et al. (2003) Proteomics of loosely bound cell wall proteins of Arabidopsis thaliana cell suspension cultures: A critical analysis. *Electrophoresis,* 24, 3421-3432.

Borner, G.H., Lilley, K.S., Stevens, T.J. & Dupree, P. (2003) Identification of glycosylphosphatidylinositol-anchored proteins in Arabidopsis. A proteomic and genomic analysis. *Plant Physiology,*132, 568-577.

Boudart, G., Jamet, E., Rossignol, M., Lafitte, C., Borderies, G. & Jauneau, A. et al. (2005) Cell wall proteins in apoplastic fluids of Arabidopsis thaliana rosettes: Identification by mass spectrometry and bioinformatics. *Proteomics,* 5, 212-221.

Calderan-Rodrigues, M.J., Jamet, E., Bonassi, M.B., Guidetti-Gonzalez, S., Begossi, A.C. & Setem, L.V. et al. (2014) Cell wall proteomics of sugarcane cell suspension cultures. *Proteomics,* 14, 738-749.

Calderan-Rodrigues, M.J., Jamet, E., Douché, T., Bonassi, M.B., Cataldi, T.R. & Fonseca, J.G. et al. (2016) Cell wall proteome of sugarcane stems: comparison of a destructive and a non-destructive extraction method showed differences in glycoside hydrolases and peroxidases. *BMC Plant Biology,* 16, 14.

Casasoli, M., Spadoni, S., Lilley, K.S., Cervone, F., De Lorenzo, G. & Mattei, B. (2008) Identification by 2-D DIGE of apoplastic proteins regulated by oligogalacturonides in *Arabidopsis thaliana*. *Proteomics,* 8, 1042-1054.

Catalá, C., Howe, K.J., Hucko, S., Rose, J.K. & Thannhauser, T.W. (2011) Towards characterization of the glycoproteome of tomato (*Solanum lycopersicum*) fruit using Concanavalin A lectin affinity chromatography and LC-MALDI-MS/MS analysis. *Proteomics,* 11, 1530-1544.

Chabi, M., Goulas, E., Leclercq, C.C., de Waele, I., Rihouey, C. & Cenci, U. et al. (2017) A cell wall proteome and targeted cell wall analyses provide novel information on hemicellulose metabolism in flax. *Molecular and Cellular Proteomics,* 16, 1634-1651.

Charmont, S., Jamet, E., Pont-Lezica, R. & Canut, H. (2005) Proteomic analysis of secreted proteins from Arabidopsis thaliana seedlings: improved recovery following removal of phenolic compounds. *Phytochemistry,* 66, 453-461.

Chen, X.Y., Kim, S.T., Cho, W.K., Rim, Y., Kim, S. & Kim, S.W. et al. (2009) Proteomics of weakly bound cell wall proteins in rice calli. *Journal of Plant Physiology,* 166, 665-685.

Chen, Y., Ye, D., Held, M.A., Cannon, M.C., Ray, T. & Saha, P. et al. (2015) Identification of the abundant hydroxyproline-rich glycoproteins in the root walls of wild-type Arabidopsis, an ext3 mutant line, and its phenotypic revertant. *Plants* 4, 85-111.

Chivasa, S., Ndimba, B.K., Simon, W.J., Robertson, D., Yu, X.L. & Knox, J.P. et al. (2002) Proteomic analysis of the Arabidopsis thaliana cell wall. *Electrophoresis,* 23, 1754-1765.

Chivasa, S., Simon, W.J., Yu, X.L., Yalpani, N. & Slabas, A.R. (2005) Pathogen elicitor-induced changes in the maize extracellular matrix proteome. *Proteomics,* 5, 4894-904.

Cho, W.K., Chen, X.Y., Chu, H., Rim, Y., Kim, S. & Kim, S.T. et al. (2009) The proteomic analysis of the secretome of rice calli. *Physiologia Plantarum,* 135, 331-341.

Dahal, D., Pich, A., Braun, H.P. & Wydra, K. (2010) Analysis of cell wall proteins regulated in stem of susceptible and resistant tomato species after inoculation with Ralstoniasolanacearum: a proteomic approach. *Plant Molecular Biology,* 73, 643–658.

Dani, V., Simon, W.J., Duranti, M. & Croy, R.R. (2005) Changes in the tobacco leaf apoplast proteome in response to salt stress. *Proteomics,* 5, 737-745.

Day, A., Fénart, S., Neutelings, G., Hawkins, S., Rolando, C. & Tokarski, C. (2013) Identification of cell wall proteins in the flax (*Linum usitatissimum*) stem. *Proteomics,* 13, 812-825.

Douché, T., San, C.H., Burlat, V., Roujol, D., Valot, B. & Zivy, M., et al. (2013) Brachypodiumdistachyon as a model plant towards improved biofuel crops: Search for secreted proteins involved in polysaccharide synthesis and disassembly. *Proteomics,* 13, 2438-2454.

Duruflé, H., Clemente, H.S., Balliau, T., Zivy, M., Dunand, C. & Jamet, E. (2016) Cell wall proteome analysis of Arabidopsis thaliana mature stems. *Proteomics,* 17, 10.1002/pmic.201600449.

Elagamey, E., Narula, K., Sinha, A., Aggarwal, P.R., Ghosh, S. & Chakraborty, N., et al. (2016) Extracellular Matrix Proteome and Phosphoproteome of Potato Reveals Functionally Distinct and Diverse Canonical and Non-Canonical Proteoforms. *Proteomes,* 4, 20.

Elagamey, E., Narula, K., Sinha, A., Ghosh, S., Abdellatef, M.A.E. & Chakraborty, N., et al. (2017) Quantitative extracellular matrix proteomics suggests cell wall reprogramming in host-specific immunity during vascular wilt caused by *Fusarium oxysporum* in Chickpea. *Proteomics,* 17, 23-24.

Elagamey, E., Sinha, A., Narula, K., Abdellatef, M.A.E. & Chakraborty, N. et al. (2017) Molecular dissection of extracellular matrix proteome reveals discrete mechanism regulating Verticilliumdahliae triggered vascular wilt disease in potato. *Proteomics,* 17, 1600373.

Elortza, F., Mohammed, S., Bunkenborg, J., Foster, L.J., Nühse, T.S. & Brodbeck, U. et al. (2006) Modification-specific proteomics of plasma membrane proteins: Identification and characterization of glycosylphosphatidylinositol-anchored proteins released upon phospholipase D treatment. *Journal of Proteome Research,* 5, 935-943.

Elortza, F., Nühse, T.S., Foster, L.J., Stensballe, A., Peck, S.C. & Jensen, O.N. (2003) Proteomic analysis of lycosylphosphatidylinositol-anchored membrane proteins. *Molecular and Cellular Proteomics,* 2, 1261-1270.

Feiz, L., Irshad, M., Pont-Lezica, R.F., Canut, H. & Jamet, E. (2006) Evaluation of cell wall preparations for proteomics: a new procedure for purifying cell walls from Arabidopsis hypocotyls. *Plant Methods,* 2, 10.

Francin-Allami, M., Lollier, V., Pavlovic, M., San, C.H., Rogniaux, H. & Jamet, E. et al. (2015) Understanding the remodeling of cell walls during *Brachypodium distachyon*

grain development through a sub-cellular quantitative proteomic approach. *Proteomes,* 4, 21.

Francin-Allami, M., Merah, K., Albenne, C., Rogniaux, H., Pavlovic, M. & Lollier, V., et al. (2015) Cell wall proteomic of Brachypodiumdistachyon grains: A focus on cell wall remodeling proteins. *Proteomics,* 15, 2296-2306.

Gokulakannan, G.G. & Niehaus, K. (2010) Characterization of the Medicagotruncatula cell wall proteome in cell suspension culture upon elicitation and suppression of plant defense. *Journal of Plant Physiology,* 167, 1533-1541.

Haslam, R.P., Downie, A.L., Raventon, M., Gallardo, K., Job, D. & Pallett, K.E. et al. (2003) The assessment of enriched apoplastic extracts using proteomic approaches. *Annals of Applied Biology,*143, 81-91.

Hervé, V., Duruflé, H., San Clemente, H., Albenne, C., Balliau, T. & Zivy, M. et al. (2016) An enlarged cell wall proteome of Arabidopsis thaliana rosettes. *Proteomics,* 16, 3183-3187.

Irshad, M., Canut, H., Borderies, G., Pont-Lezica, R. & Jamet, E. (2008) A new picture of cell wall protein dynamics in elongating cells of Arabidopsis thaliana: confirmed actors and newcomers. *BMC Plant Biology,* 8, 94.

Jamet, E., Roujol, D., San-Clemente, H., Irshad, M., Soubigou-Taconnat, L. & Renou, J.P. et al. (2009) Cell wall biogenesis of Arabidopsis thaliana elongating cells: transcriptomics complements proteomics. *BMC Genomics,* 10, 505.

Jung, Y.H., Jeong, S.H., Kim, S.H., Singh, R., Lee, J.E. & Cho, Y.S. et al. (2008) Systematic secretome analyses of rice leaf and seed callus suspension-cultured cells: Workflow development and establishment of high-density two-dimensional gel reference maps. *Journal of Proteome Research,* 7, 5187-5210.

Komatsu, S., Kobayashi, Y., Nishizawa, K., Nanjo, Y. & Furukawa, K. (2010) Comparative proteomics analysis of differentially expressed proteins in soybean cell wall during flooding stress. *Amino Acids,* 39, 1435-49.

Kong, F.J., Oyanagi, A. & Komatsu, S. (2010) Cell wall proteome of wheat roots under flooding stress using gel-based and LC MS/MS-based proteomics approaches. *Biochimica et Biophysica Acta,* 1804, 124-136.

Kumar, S., Kumar, K., Pandey, P., Rajamani, V., Padmalatha, K.V. & Dhandapani, G. et al. (2013) Glycoproteome of elongating cotton fiber cells. *Molecular and Cellular Proteomics,* 12, 3677-3689.

Kwon, H.K., Yokoyama, R. & Nishitani, K.A. (2005) Proteomic approach to apoplastic proteins involved in cell wall regeneration in protoplasts of Arabidopsis suspension-cultured cells. *Plant and Cell Physiology*, 46, 843-857.

Lim, S., Chisholm, K., Coffin, R.H., Peters, R.D., Al-Mughrabi, K.I. & Wang-Pruski, G. et al. (2012) Protein profiling in potato (Solanumtuberosum L.) leaf tissues by differential centrifugation. *Journal of Proteome Research,* 11, 2594-2601.

Martínez-Cortés, T., Pomar, F., Merino, F. & Novo-Uzal, E. (2014) A proteomic approach to Physcomitrella patens rhizoid exudates. *Journal of Plant Physiology,* 171, 1671-1678.

Martinez-Esteso, M.J., Sellés-Marchart, S., Vera-Urbina, J.C., Pedreño, M.A. & Bru-Martinez, R. (2009) Changes of defense proteins in the extracellular proteome of grapevine (Vitisvinifera cv. Gamay) cell cultures in response to elicitors. *Journal of Proteomics,* 3, 331-341.

Millar, D.J., Whitelegge, J.P., Bindschedler, L.V., Rayon, C., Boudet, A.M. & Rossignol, M. et al. (2009) The cell wall and secretory proteome of a tobacco cell line synthesising secondary wall. *Proteomics,* 9, 2355-2372.

Minic, Z., Jamet, E., Négroni, L., Arsene der Garabedian, P., Zivy, M. & Jouanin, L. (2007) A sub-proteome of Arabidopsis thaliana trapped on Concanavalin A is enriched in cell wall glycoside hydrolases. *Journal of Experimental Botany,* 58, 2503-2512.

Ndimba, B.K., Chivasa, S., Hamilton, J.M., Simon, W.J. & Slabas, A.R. (2003) Proteomic analysis of changes in the extracellular matrix of Arabidopsis cell suspension cultures induced by fungal elicitors. *Proteomics,* 3, 1047-59.

Nguyen-Kim, H., San, C.H., Balliau, T., Zivy, M., Dunand, C. & Albenne, C. et al. (2016) Arabidopsis thaliana root cell wall proteomics: increasing the proteome coverage using a combinatorial peptide ligand library and description of unexpected Hyp in peroxidase amino acid sequences. *Proteomics,* 16, 491-503.

Pandey, A., Rajamani, U., Verma, J., Subba, P., Chakraborty, N. & Datta, A. et al. (2010) Identification of extracellular matrix proteins of rice (*Oryza sativa* L.) involved in dehydration-responsive network: a proteomic approach. *Journal of Proteome Research,* 9, 3443-3464.

Pechanova, O., Hsu, C.Y., Adams, J.P., Pechan, T., Vandervelde, L. & Drnevich, J. et al. (2010) Apoplast proteome reveals that extracellular matrix contributes to multistress response in poplar. *BMC Genomics,* 11, 674.

Printz, B., Dos Santos Morais, R., Wienkoop, S., Sergeant, K., Lutts, S. & Hausman, J.F. et al. (2015) An improved protocol to study the plant cell wall proteome. *Frontiers in Plant Science,* 6, 237.

Robertson, D., Mitchell, G.P., Gilroy, J.S., Gerrish, C., Bolwell, G.P. & Slabas, A.R. (1997) Differential extraction and protein sequencing reveals major differences in patterns of primary cell wall proteins from plants. *Journal of Biological Chemistry*, 272, 15841-15848.

Schultz, C.J., Ferguson, K.L., Lahnstein, J. & Bacic, A. (2004) Post-translational modifications of arabinogalactan-peptides of Arabidopsis thaliana. Endoplasmic reticulum and glycosylphosphatidylinositol-anchor signal cleavage sites and hydroxylation of proline. *Journal of Biological Chemistry,* 279, 45503-45511.

Sergeant, K., Printz, B., Guerriero, G., Renaut, J., Lutts, S. & Hausman, J.F. (2019) The dynamics of the cell wall proteome of developing alfalfa stems. *Biology (Basel),* 8, pii: E60.

Sultana, N., Florance, H.V., Johns, A. & Smirnoff, N. (2015) Ascorbate deficiency influences the leaf cell wall glycoproteome in Arabidopsis thaliana. *Plant, Cell & Environent,* 38, 375-384.

Swaroopa Rani, T. & Podile, A.R. (2014) Extracellular matrix-associated proteome changes during non-host resistance in citrus–Xanthomonas interactions. *Plant Physiology,* 150, 565-579.

Thannhauser, T.W., Shen, M., Sherwood, R., Howe, K., Fish, T. & Yang, Y. et al. (2013) A workflow for large-scale empirical identification of cell wall N-linked glycoproteins of tomato (*Solanum lycopersicum*) fruit by tandem mass spectrometry. *Electrophoresis,* 34, 2417-2431.

Trentin, A.R., Pivato, M., Mehdi, S.M., Barnabas, L.E., Giaretta, S. & Fabrega-Prats, et al. (2015) Proteome readjustments in the apoplastic space of Arabidopsis thaliana ggt1 mutant leaves exposed to UV-B radiation. *Frontiers in Plant Science,* 6, 128.

Verdonk, J.C., Hatfield, R.D. & Sullivan, M.L. (2012) Proteomic analysis of cell walls of two developmental stages of alfalfa stems. *Frontiers in Plant Science,* 3, 279.

Watson, B.S., Lei, Z., Dixon, R.A. & Sumner, L.W. (2004) Proteomics of *Medicago sativa* cell walls. *Phytochemistry,* 65, 1709-1720.

Wu, Y., Sharp, R.E., Durachko, D.M. & Cosgrove, D.J. (1996) Growth maintenance of the maize primary root at low water potentials involves increases in cell-wall extension properties, expansin activity, and wall susceptibility to expansins. *Plant Physiology,* 111, 765-772.

Xu, S.L., Medzihradszky, K.F., Wang, Z.Y., Burlingame, A.L. & Chalkley, R.J. (2016) N-Glycopeptide profiling in Arabidopsis inflorescence. *Molecular and Cellular Proteomics,* 15, 2048-2054.

Yeats, T.H., Howe, K.J., Matas, A.J., Buda, G.J., Thannhauser, T.W. & Rose, J.K. (2010) Mining the surface proteome of tomato (*Solanum lycopersicum*) fruit for proteins associated with cuticle biogenesis. *Journal of Experimental Botany,* 61, 3759-3771.

Zhang, M., Chen, G.X., Lv, D.W., Li, X.H. & Yan, Y.M. (2015) N-linked glycoproteome profiling of seedling leaf in Brachypodiumdistachyon L. *Journal of Proteome Research,* 14, 1727-1738.

Zhang, Y., Giboulot, A., Zivy, M., Valot, B., Jamet, E. & Albenne, C. (2010) Combining various strategies to increase the coverage of the plant cell wall glycoproteome. *Phytochemistry,* 72, 1109-1123.

Zhou, L., Bokhari, S.A., Dong, C.J. & Liu, J.Y. (2011) Comparative proteomics analysis of the root apoplasts of rice seedlings in response to hydrogen peroxide. *PLoS ONE* 6, e16723.

Zhu, J., Alvarez, S., Marsh, E.L., Lenoble, M.E., Cho, I.J., Sivaguru, M., (2007) Cell wall proteome in the maize primary root elongation zone. II. Region-specific changes in water soluble and lightly ionically bound proteins under water deficit1[W][OA]. *Plant Physiology,*145, 1533-1548.

Zhu, J., Chen, S., Alvarez, S., Asirvatham, V.S., Schachtman, D.P., Wu, Y., et al. (2006) Cell wall proteome in the maize primary root elongation zone. I. Extraction and identification of water-soluble and lightly ionically bound proteins. *Plant Physiology,* 140, 311-325.
