# Supplementary material for: Combining extracellular matrix proteome and phosphoproteome of chickpea and meta‐analysis reveal novel proteoforms and evolutionary significance of clade‐specific wall‐associated events in plant
Source: Plant Direct. 2024 Mar 18;8(3):e572. doi: 10.1002/pld3.572 (PMC10945595; doi:10.1002/pld3.572)
Supplement: Supplementary file 12 — Data S1. Peer Review. [file PLD3-8-e572-s001.pdf]

Comment 1. The revised manuscript answers some of the concerns raised by the reviewers. However, in this particular case reviewers were somewhat vague in some of the criticism during the first round of revisions, and thus I decided to provide my own review with a little more detail in areas in which some detail is still missing, or where more clarity is needed in order to make the manuscript easier to follow and interpret.

I have attached a word document with my revisions and comments. I also took the opportunity to provide some minor editing in places where it could contribute to readability. The main comments related to the need for additional details in some of the Material and Methods, and to passages in the Results section that do not seem to correspond to the figure that is referred in the paragraph.

Please provide a revised version that takes into account my comments, and I will make a decision without further rounds of reviews.

Thank you for submitting your manuscript to Plant Direct.

Reply 1: We sincerely appreciate editor's concern and carefully editing the manuscript with his thoughtful remarks. Further, we are thankful to the editor for providing minor editing and improve readability of the manuscript. We have revised the manuscript as advised.

All the mirror editing's are incorporated in the revised version of the manuscript. The comments along with our replies are provided here.

Editor's Comment

Comment 1. A qualifier was missing here. Is this what you wanted to say?

Reply 1: Yes. We agree with the editor.

Comment 2. This addition was mentioned in the response to reviewers but was missing here.

Reply 2: This was an inadvertent mistake. In the abstract section Page 2 Line 65 **"**This is the first report on comprehensive matrisome proteoform network illustrating presence of CWI signaling proteins in early land plants" has been changed to "This is the first report on comprehensive matrisome proteoform network illustrating presence of CWI signaling proteins in land plants".

Comment 3: Not needed. Since plants have only been reported in Planet Earth so far, there is no need for location here

Reply 3: Yes, we agree. The location name has now been removed in the revised manuscript.

Comment 4: Why is the term "ECM" used instead of "cell wall"? General plant science audience is more familiar with the concept of "cell wall". Whether ECM is used to differentiate specific parts of the cell wall, or as a term equivalent to cell wall should be clarified.

Reply 4: Extracellular molecules secreted by animal and plant cells are termed as Extracellular Matrix (ECM). It is composed of polysaccharides and proteins, having important functions in different tissues, regulating intercellular adhesion and communication. It is dynamic, serves as physical scaffold to the cell and remodelled by physiological cell conditions including homeostasis response to diseases. With the exception of animal and protozoal, the majority of cell types are covered by a cell wall (CW). The name CW describes the characteristics of

rigidity, support, and actual shape conferred by this particular ECM to plant cells and thus ECM and CW is synonymous in plant (**Guerriero et al., 2014; Stavolone and Lionetti, 2017**).

In our future studies, we will be comparing cross-kingdom matrisome, including plant matrisome (reported in this study), animal, fungal and protozoal matrisome to identify unique and shared features of different matrisome. With this aspect in mind, we have used the term ECM in this study and request to allow us retain the same nomenclature.

References

1. Stavolone, L& Lionetti, V. (2017) Extracellular Matrix in Plants and Animals: Hooks and Locks for Viruses. *Frontiers in Microbiology*, 8:1760.
2. Guerriero, G., Hausman, J.F. & Cai, G. (2014). No stress! Relax! Mechanisms governing growth and shape in plant cells. *International Journal of Molecular Sciences,* 15, 5094-5114.

Comment 5: Is this what you wanted to say?

Reply 5: Yes. The sentence in Page 5, Line 126-128 has been revised accordingly.

Comment 6: Other phosphoproteome analyses of cell wall already exist. This seems like an exaggeration.

Reply 6: Several reports are available for animal and fungal ECM phosphoproteme analysis. In plant, phosphoproteome analysis have been studied for fibers only till date. However, no report is available which described isolation of ECM fraction followed by phosphoprotein enrichment and LC-MS/MS analysis. Therefore, we mentioned this study as a first report that analyse matrix specific S/T/Y phosphorylation events in plants.

Comment 7: The manuscript has a significant number of supplemental tables (this is appreciated, and it adds value to the manuscript). As described in the journal's Author Guidelines, there should be a list with the titles of each piece of material at the end of Methods. The title should be descriptive, so readers can know what is in each table. Additionally, I recommend to include a title/description in each excel file to help readers understand the content.

Reply 7: As suggested, we have provided list of supplementary materials after Materials and Methods section and provided description of each table in the revised version of the manuscript.

Comment 8: How much sample? The figure also shows crude extract, please describe

Reply 8: As suggested, we have added the amount of sample in the Materials and Methods subsection 2.4 "In brief, glucose-6 phoshate dehydrogenase activity was determined in 10 µg each of ECM protein extract and crude protein extract by spectrophotometer." at Page 7 Line 192 of the revised manuscript.

Comment 9: I think there is no doubt that Fabaceae are cosmopolitan and adapted to land

Reply 9: Yes. We agree with the editor. The manuscript has been revised accordingly.

Comment 10: Please fix (is it 1,221,324?)

Reply 10: We have fixed the issue in the revised manuscript.

Comment 11: This is difficult to follow. Please identify each category using letters. For example, "…into five categories: a) cell wall biogenesis, architecture, development and remodeling; b) redox homeostasis…"

Reply 11: As suggested, we have identified each functional category with a letter in the Results subsection 3.1, Page 19, Line 492-495 of the revised manuscript.

Comment 12: This figure only shows taxonomic families, but not functional categories. Please refer to correct figure or elaborate on how the extra categories were inferred

Reply 12: It was an inadvertent mistake. Figure 2c represents Taxonomic family-based matrisome network. We placed Figure 2c at Page 21 Line 546 in the revised manuscript. Further, Page 21, Line 546-550 describe ECM proteins in taxonomic family network and its correlation to physicochemical properties in the revised manuscript. Likewise, Page 21, Line 546-550 has been changed to "Matrix proteins mainly existed in hubs, which included carbohydrate degrading enzymes, proteases and structural proteins. Commonality in segregated hubs is multidomain and complex structural attributes of proteoforms and diversity existed in aminoacid sequences, and physicochemical properties (Figure 2a-c)" in the revised manuscript.

Comment 13: Again, how is this inferred, if the figure only shows grouping by organ?

Reply 13: It was an inadvertent mistake. Figure 2d represents relationship between matrix integrity proteins reported from different organ/cell suspension. We have now placed Figure 2d at Page 21 Line 552 in the revised manuscript.

Comment 14: Is this information presented in a table, supplemental table or figure? The figures mentioned in the text do not seem to correspond to the functional information described.

Reply 14: As suggested, we have incorporated a column in Table S4 "List of ECM proteins reported in available ECM proteomes" having information of organ from which the particular ECM protein was identified in respective organism. Likewise, Table S4 was updated in the revised manuscript.

Comment 15: Please rephrase. The sentence is unclear. May be describing the network in Fig 2e first, and then explaining the grouping into families in Fig 2f would make it more clear. Also, there is no indication of what the colors mean in Fig 2f, please add to the legend.

Reply 15: As suggested, we have rephrased **"**we segregated matrisome into 8 ECM protein families network identified from somatic organ, reproductive organ and both (Figure 2e-f)" to **"**we developed ECM protein family network from eight families, which were identified in the matrisome from somatic organ, reproductive organ and both (Figure 2e) in the revised manuscript. Further, we have added results related to Figure 2e in the Results subsection 3.3 "Systematic prioritization of CWI proteins in organ and taxonomic clade network and their diversification across plant lineages" in Page 22, Line 572-579.

Since the eight protein families were studied for their interactions as well as for physicochemical properties, such as, whether protein family members were labile, salt-extracted or strongly bound ECM proteins, these results were discussed together for each family to provide better insight into interaction and physicochemical properties of each family

in Figure 2e-f. As suggested, we have provided the color code for Figure 2f in the revised manuscript.

Comment 16: Where is this presented? There is no "ion flux" category, or any category that could be interpreted as primarily ion flux

Reply 16: We have edited "ion flux" to "oxidoreductases" in the Results subsection 3.3 "Systematic prioritization of CWI proteins in organ and taxonomic clade network and their diversification across plant lineages" in Page 22, Line 579 of the revised manuscript.

Comment 17: I don't understand this section. It seems to say that carbohydrate modifying enzymes acting on glucose and arabinose were specific to some families and at the same time common to all families. The statements contradict each other. Please clarify.

Reply 17: As suggested, we have clarified these statements by providing names of carbohydrate modifying enzymes acting on glucose and arabinose, such as, UDP-glucose:protein transglucosylase, UDP-arabinopyranose mutase and glycosyl transferase family specific to Brassicaceae, Fabaceae, Rosids and Poaceae. However, carbohydrate modifying enzymes acting on glucose, xylose, xylulose, galactose, ribose and arabinose, such as, arabinofuranosidase beta, Glucan Exohydrolase beta, 1 4-glucan-protein synthase alpha, xylanase, polygalacturonase-inhibiting protein and Ribose-5-phosphate isomerase were common in Funarales, Rosids, Asterids and Poales. Furthermore, we have mentioned the related information in Table S4 in the revised manuscript.

Comment 18: Some of the symbols in Fig 4a, Fig 4b and Fig 4c are not placed over the lines in the phylogenetic trees. Can this be fixed?

Reply 18: We have corrected and placed the symbols over the line in Figure 4a-c in the revise manuscript.

Comment 19: It is not clear that the lower part of Fig 4b corresponds to Papilionioideae. Can a label be added to improve clarity?

Reply 19: As suggested, we have mentioned Papilionioideae in the lower part of Figure 4b in the revised manuscript.

Comment 20: This was already mentioned, as cellulose was found to be ubiquitous. It would be more important to mention that it is apparent that there is low coverage for many of the plants included, and that ECM components that probably exist are missing in the analysis (it is important to indicate the limitations of the study, otherwise it loses credibility).

Reply 20: As suggested, we have added a sentence **"**Till date very few members of cellulose metabolism (4 proteins) have been identified in many of the plant ECM proteomes, indicating the low coverage of plant matrisome". In the Results subsection 3.4 "Relationship between evolution of plant kingdom, matrix diversity and annotation of orthologous clusters across plant families at the proteome scale" in Page 24, Line 637-639 in the revised manuscript.

Comment 21: I don't think this is supported (the sentence is not clear, so I may be missing the intended message).

Reply 21: We have rephrased the sentence **"**Another class included protein with interacting domain, 31% of which was from reproductive organs and might act as developmental determinants across lineages" to **"**Protein with interacting domain is another important functional class in the matrisome having 356 ECM proteins, 31% of which was from reproductive organs and might involve in plant development across lineages". In the Results subsection 3.4 "Relationship between evolution of plant kingdom, matrix diversity and annotation of orthologous clusters across plant families at the proteome scale" at Page 25, Line 647-649 in the revised manuscript.

Comment 22: What is the meaning of "lack discrete functionality" here? The list of proteins included in the category seem to have functions.

Reply 22: Proteins which were not predicted to be identified in all the cell wall proteomes characterized so far were named non-canonical CWPs (Clemente et al., 2022). The functions of these proteins are not described in the extracellular matrix, therefore, we have used the term "lack discrete functionality". However, for better understanding, we have changed "Miscellaneous ECM proteins that lack discrete functionality" to "Miscellaneous ECM proteins that are non-canonical" In the Results subsection 3.4 "Relationship between evolution of plant kingdom, matrix diversity and annotation of orthologous clusters across plant families at the proteome scale" at Page 25, Line 651 in the revised manuscript.

Reference

San Clemente, H., Kolkas, H., Canut, H. & Jamet, E. (2022) Plant Cell Wall Proteomes: The Core of Conserved Protein Families and the Case of Non-Canonical Proteins. *International Journal of Molecular Sciences*, 23(8):4273.

Comment 23: "Unknown proteins" usually means proteins for which the function is unknown. Thus, they cannot have "known functionality". Did you intend to say that are known to be in the CW? In that case, you can explicitly say that they are in CW but their function in this organ is not known.

Reply 23: We would like to clarify that according to Mewalal et al., 2014, cell wall protein-protein of unknown function (CW-PUFs) refer to any functionally unannotated proteins which have some evidence linking them to cell wall biology. CW-PUFs has two categories: (i) proteins of known function; and (ii) proteins of unknown function (PUF) those do not have experimental evidence demonstrating function in cell wall biology yet are implicated in this process based on co-expression analysis. Such proteins may even have validated roles in other biological processes. They refer to this group as CW-known unknown.

Based on the abovementioned classification, we have edited "Finally, matrisome included CW-known unknown proteins (28%) with known functionality but lacking experimental evidence was distributed in 54 species from Rosids, Asterids and Poales" to "Finally, matrisome included CW-known unknown proteins (28%) with known functionality in biological processes but lacking experimental evidence demonstrating function in cell wall biology was distributed in 54 species from Rosids, Asterids and Poales." In the Results subsection 3.4 "Relationship between evolution of plant kingdom, matrix diversity and annotation of orthologous clusters across plant families at the proteome scale" at Page 25, Line 652-655 in the revised manuscript.

Reference

Mewalal, R., Mizrachi, E., Mansfield, S.D. & Myburg, A.A. (2014) Cell wall-related proteins of unknown function: missing links in plant cell wall development. Plant Cell Physiology, 55,1031-43.

Comment 24: It is not clear what scenario or hypothesis is being referred to here

Reply 24: We have edited the sentence "This scenario is not the most parsimonious hypothesis and subject to periodic revision in light of future discoveries." to "The presence of cell wall-unknown protein repertoire is not the most parsimonious hypothesis and should be subjected to periodic revision in light of future discoveries". In the Results subsection 3.4 "Relationship between evolution of plant kingdom, matrix diversity and annotation of orthologous clusters across plant families at the proteome scale" Page 25, Line 660-662 in the revised manuscript.

Comment 25: What is represented in the first panel of Fig5a? Please make the legend more descriptive.

Reply 25: As suggested, we have elaborated Figure 5a in the revised manuscript.

Comment 26: Please define

Reply 26: As suggested, we have defined CAZy as Carbohydrate-Active enzymes in the Results subsection 3.5 "Analysis of the sequence divergence variation, patterns of gene gains and loss through gene duplications, gene expansion and inference of proportion of sites under selection" at Page 27, Line 699 in the revised manuscript.

Comment 27: This is a sugar motif, not a protein. Please include full protein name

Reply 27: We have provided the full protein name as (Hex)4 (HexNAc)2-folate binding protein in the Results subsection 3.7 "Functional characterization of ECM proteins across Cryptogamia, Eudicot and Monocot" at Page 32, Line 846 in the revised manuscript.

Comment 28: Reference?

Reply 28: We have provided references for the statement "The proteoforms, included isoforms of lipase, peroxidase, dismutase, pectinesterase, pectinmethylesterase and glycoside hydrolase family 19 found to be involved in recognition, wall carbohydrate rearrangement and degradation" enzymes in the Results subsection 3.7 "Functional characterization of ECM proteins across Cryptogamia, Eudicot and Monocot" at Page 32, Line 855-856 in the revised manuscript.

References:

Bradley, E.L., Ökmen, B., Doehlemann, G., Henrissat, B., Bradshaw, R.E. & Mesarich, C.H. (2022) Secreted Glycoside Hydrolase Proteins as Effectors and Invasion Patterns of Plant-Associated Fungi and Oomycetes. *Frontiers in Plant Science*, 13:853106.

Jolie, R.P., Duvetter, T., Van Loey, A.M., Hendrickx, M.E. (2010) Pectin methylesterase and its proteinaceous inhibitor: a review. *Carbohydrate Research*, 345, 2583-2595.

Guerra-Guimarães, L., Pinheiro, C., Chaves, I., Barros, D.R. & Ricardo, C.P. (2016) Protein dynamics in the plant extracellular space. *Proteomes,* 4, 22.

Comment 29: Plants do not have canonical fibronectin, although a protein with a fibronectin-like domain has been reported. Please modify accordingly.

Reply 29: We have edited "fibronectin" to "fibronectin-like domain containing protein" in the Discussion section at Page 36-37, Line 972-973 in the revised manuscript.
